# Oat bran fiber protects against radiation-induced disruption of gut barrier dynamics and mucosal damage

**DOI:** 10.1038/s41522-025-00759-x

**Published:** 2025-07-04

**Authors:** Piyush Patel, Chunsheng Jin, Intawat Nookaew, Michael Robeson, Dilip K. Malipatlolla, Sravani Devarakonda, Ana Rascón, Margareta Nyman, Niclas G. Karlsson, Agnes E. Wold, Fei Sjöberg, Cecilia Bull

**Affiliations:** 1 Division of Clinical Cancer Epidemiology, Department of Oncology, Institute of Clinical Sciences, Sahlgrenska Academy, University of Gothenburg, Gothenburg, Sweden; 2 Department of Infectious Diseases, Institute of Biomedicine, Sahlgrenska Academy, University of Gothenburg, Gothenburg, Sweden; 3 Department of Medical Biochemistry and Cell Biology, Institute of Biomedicine, Sahlgrenska Academy, University of Gothenburg, Gothenburg, Sweden; 4 Proteomics Core Facility at Sahlgrenska Academy, University of Gothenburg, Gothenburg, Sweden; 5 Department of Biomedical Informatics, College of Medicine, University of Arkansas for Medical Sciences, Little Rock, AR 72205 USA; 6 Department of Process and Life Science Engineering, Division of Food and Pharma, Lund University, Lund, Sweden; 7 Department of Life Sciences and Health, Faculty of Health Sciences, Oslo Metropolitan University, 0130 Oslo, Norway

**Keywords:** Biological techniques, Microbiology, Health care

## Abstract

Dietary fibers are recognized for their health benefits, yet cancer patients undergoing pelvic radiotherapy are often advised to reduce fiber intake. This may negatively impact their bowel health. To evaluate the effects of dietary fibers on bowel health post-irradiation, male C57BL/6 mice were fed diets containing either 0 or 15% fiber with varying proportions of readily fermentable bioprocessed oat bran fiber and/or poorly fermentable microcrystalline cellulose, and either irradiated or sham-irradiated. Irradiation triggered mucus degradation and depletion of short-chain fatty acids, and a fiber-free diet exacerbated radiation-induced mucosal damage. In contrast, mice fed oat bran fiber exhibited less mucosal damage, fewer dysbiotic and mucus-degrading bacteria, higher production of short-chain fatty acids, and improved bactericidal activity. These benefits were dose-dependent, with 15% oat bran fiber providing greater protection. Our findings suggest that fiber deprivation exacerbates radiation-induced intestinal damage, while supplementation with 15% highly fermentable oat bran fiber supports mucosal integrity and protects against radiation-induced injury.

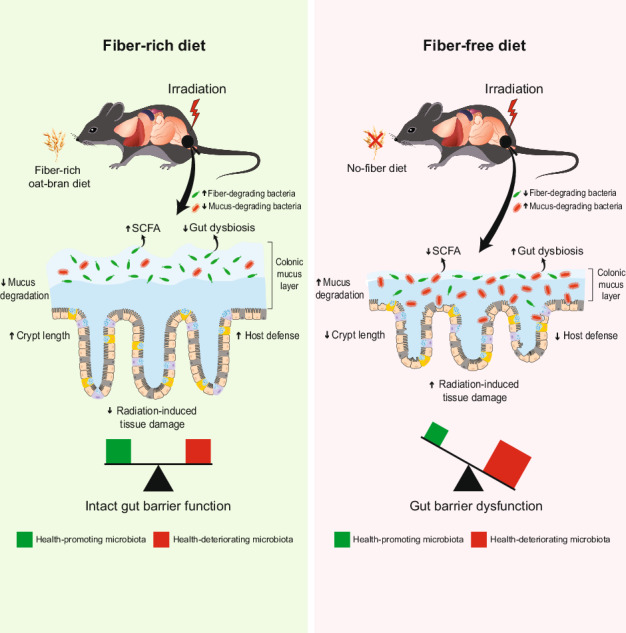

## Introduction

Worldwide, the impact of pelvic radiotherapy on bowel function affects millions of cancer survivors^1^. Unwanted radiation exposure to the distal bowels during treatment for gynecological, prostate, anal, and rectal cancers can lead to lifelong dysfunctions, including diarrhea, fecal urgency, incontinence, tenesmus, and rectal bleeding.^2,3^.

Radiation-induced mucosal injury includes inflammation, ischemia and fibrosis, with limited treatment options beyond symptom-relieving strategies^4^. A common recommendation is to limit fiber intake during and after radiotherapy. The advice aims to alleviate additional bowel discomfort produced by the fermentation of dietary fiber, but there is limited understanding of how this dietary restriction may affect bowel health post-radiotherapy. Diet shapes the gut ecosystem, and dietary fiber, in particular, has a significant impact^5^. The interaction between the microbes colonizing the mucus on the mucosal surface and the colonocytes of the mucosa is crucial for maintaining gut health. Bacteria ferment complex carbohydrates present in dietary fiber into short-chain fatty acids (SCFAs), including propionate, acetate and butyrate. These SCFAs, especially butyrate, serve as a major energy source for the colonocytes. Importantly, it allows the goblet cells to produce mucus that protects the host from toxic luminal contents and invasive pathogens.

When dietary fibers are scarce, certain bacterial species, such as *Bacteroides thetaiotaomicron*, switch from fermenting dietary fibers to degrading host mucus glycans as an energy source, thereby eroding the colonic mucus barrier^6^. A diet low in fiber may also promote the expansion of primarily mucus-degrading bacteria, such as *Akkermansia muciniphila*, further exacerbating mucus barrier erosion^7^. The intestinal mucus barrier is the first line of defense against both commensal bacteria and invading pathogens^8^. Mucin-2 (Muc2), the primary gel-forming mucin in the colon, is heavily O-glycosylated with carbohydrates accounting for up to 80% of the total mucin mass^9^. The degradation of Muc2 can lead to the invasion of bacteria into the mucosa, triggering an inflammatory response^10^.

We recently showed that cancer survivors who have undergone pelvic radiotherapy exhibit permanent damage to the protective mucus layers with bacterial infiltration into the underlying mucosa and low-grade, chronic mucosal inflammation^11^. Additionally, using a mouse model of pelvic radiotherapy, we showed that intake of dietary oat bran fiber completely blocked radiation-induced bacterial infiltration^12^. Therefore, the advice to reduce dietary fiber intake may be harmful, especially for pelvic cancer patients undergoing or recovering from radiotherapy. However, there is currently insufficient scientific evidence to develop specific dietary guidelines for this patient group. This study aims to evaluate how dietary fiber intake and dietary fibers with different levels of fermentability modify radiation-induced intestinal injury, with a focus on factors supporting mucosal barrier integrity and gut homeostasis.

## Results

### Experimental diets

The experimental design and diets are described in detail in the methods section and in Fig. 9. In brief, five experimental diets were used. One diet was fiber-free, while the other four contained bioprocessed oat bran and/or microcrystalline cellulose (MCC) to provide a total fiber content of 15%, with counterbalanced ratios of the respective fibers. Mice were exposed to irradiation (irr) two weeks after the introduction of the experimental diets. By this time, major diet-related changes in the microbiota and mucus barrier composition were expected to have stabilized, and any stress from the diet shift had likely subsided as the mice acclimated. The five irradiated groups that received diets with different fiber compositions were as follows:

- **High-oat irr:** A diet containing 15% fiber, consisting entirely of bioprocessed oat bran fiber.
- **Medium-oat irr:** A diet containing 15% fiber, consisting of 10% bioprocessed oat bran fiber and 5% MCC fiber.
- **Low-oat irr:** A diet containing 15% fiber, consisting of 5% bioprocessed oat bran fiber and 10% MCC fiber.
- **No-oat irr:** A diet containing 15% fiber, consisting entirely of MCC fiber.
- **No-fiber irr:** A diet containing 0% fiber (fiber-free).

Additionally, two control groups, high-oat cntl and no-fiber cntl, were fed the same diets as the high-oat irr and no-fiber irr groups but were sham-irradiated. The diets were administered until six weeks post-irradiation.

### Fiber-deprived diet causes severe intestinal tissue damage with decreased crypt depth

**Fig. 1:**
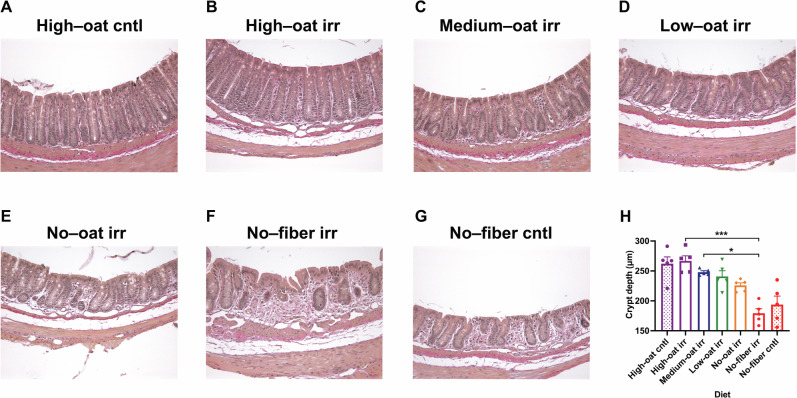
Dietary fiber deficiency damages intestinal morphology. Colorectal tissues were stained with Verhoeff-Van Gieson stain to visualize the mucosal tissue histology (40× magnification) (**A–G**). Colon crypt depths were measured under a Leica DM6000B microscope (**H**), *n* = 5 mice/group; *p ≤ 0.05, ***p ≤ 0.001.

To evaluate the effects of the experimental diets on the mucosal tissue damage post-irradiation, mice were sacrificed 6 weeks after irradiation, and histological changes in colorectal tissues were examined. The detrimental effect of irradiation was most pronounced in the No-fiber irr group, which exhibited reduced crypt depth, an indicator of intestinal tissue damage, compared to the other irradiated groups (Fig. 1B–F). In addition, significantly shorter crypts were observed in the colorectal tissues of the No-fiber irr mice compared to the High-oat irr and Medium-oat irr groups (*p* < 0.001 and *p* < 0.05, respectively) (Fig. 1H). Despite not being irradiated, the no-fiber cntl mice also showed increased tissue damage with very short crypts, similar to the no-fiber irr group (Fig. 1G, H). In contrast, the High-oat irr and High-oat cntl mice had longer crypts than mice from other groups, with fewer signs of radiation-induced intestinal damage in the High-oat irr group. These results indicate that fermentable oat bran fiber protects the mucosal architecture from known hallmarks of radiation-induced intestinal damage, while fiber deprivation alone leads to pronounced radiation-induced injury.

### Dietary fiber intake dominates over irradiation in shaping gut microbial composition

**Fig. 2:**
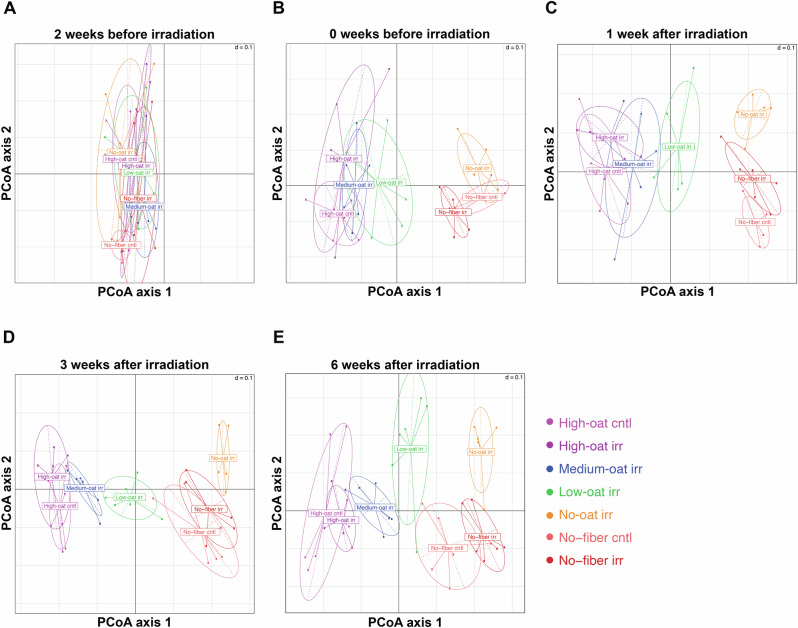
PCoA plot using the Bray-Curtis dissimilarity index illustrating differences in gut microbiota composition between groups over time. Fecal samples were analyzed at 2 weeks (**A**) and 0 weeks (**B**) before irradiation, and at 1 week (**C**), 3 weeks (**D**), and 6 weeks (**E**) after irradiation; *n* = 6 mice/group at each time point, for a total of 210 samples.

Dysbiosis in the colon can lead to mucus degradation and chronic inflammation. Therefore, we examined the gut microbiota of the mice at five distinct time points. At baseline, all seven groups (excluding the no-fiber cntl group) clustered together in the principal coordinates analysis (PCoA) plot, confirming that the groups had a similar gut microbial composition before the introduction of the various diets (Fig. 2A). After 2 weeks on the experimental diets and before irradiation, the microbial composition of the fiber-deprived group (no-fiber irr) differed markedly from those of the groups receiving a fiber-rich diet (high-, medium-, low- and no-oat irr) (Fig. 2B). The microbiota composition of the No-fiber irr group was statistically different from that of the high-, medium-, low-, and no-oat irr groups (adjusted *p* values = 0.006 for all comparisons) (Supplementary Table 1). At 1 week after irradiation, mice that consumed medium to high amounts of fermentable oat bran fiber exhibited a similar microbiota composition, which differed from that of the other groups (Fig. 2C). However, at 3 and 6 weeks after irradiation, all the irradiated groups had distinct microbiota compositions compared to each other (Fig. 2D, E). At 6 weeks after irradiation, the no-fiber irr group had a distinct microbiota composition compared to the high-, medium-, low-, and no-oat irr groups (adjusted *p* values = 0.0063, 0.0063, 0.0112, and 0.0063, respectively) (Supplementary Table 1). Notably, irradiation itself appeared to have limited influence on the overall gut microbiota composition of the mice compared to the diet, with no statistically significant differences in microbiota composition between the High-oat irr and High-oat cntl groups (adjusted *p* value = 0.282) or between the no-fiber irr and no-fiber cntl groups (adjusted *p* value = 0.1533) (Fig. 2E).

### Fiber-deprived diet causes increase in the *Firmicutes/Bacteroidetes* ratio

**Fig. 3:**
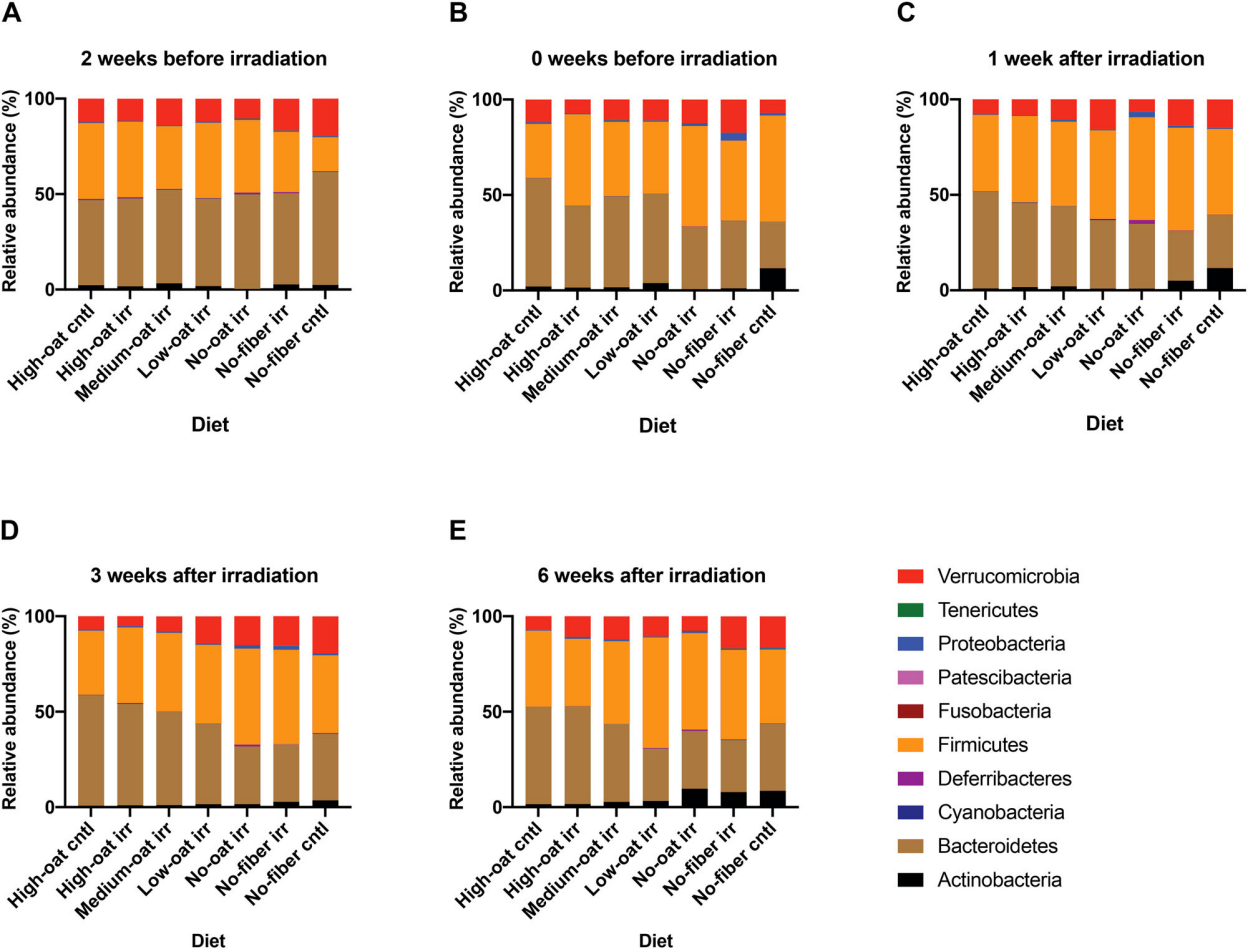
Changes in relative bacterial abundances over time at the phylum level. The mean relative abundances (%) of the bacterial taxa were determined at the phylum level for the groups, at 2 weeks (**A**) and 0 weeks (**B**) before irradiation, and at 1 week (**C**), 3 weeks (**D**), and 6 weeks (**E**) after irradiation; *n* = 6 mice/group at each time point.

The impact of diet and irradiation on bacterial phyla and OTUs in fecal samples were analyzed across all dietary groups at five time points. Ten phyla were detected. Two weeks before irradiation, phyla abundances were similar across groups, except for the no-fiber cntl group, which had a lower abundance of *Firmicutes* and higher abundance of *Bacteroidetes* and *Verrucomicrobia* (Fig. 3A). Two weeks after the introduction of the experimental diets but before irradiation (0 weeks before irradiation), *Firmicutes* increased in groups consuming oat bran-deprived diets (no-oat and no-fiber diets), at the expense of the *Bacteroidetes* (Fig. 3B). This pattern persisted post-irradiation. At 1, 3, and 6 weeks after irradiation, the groups consuming a high amount of fermentable oat bran fiber (high-oat irr and high-oat cntl) continued to have a high abundance of *Bacteroidetes*, while the groups consuming high amounts of poorly fermentable microcrystalline cellulose or no fiber (low-oat, no-oat, and no-fiber) showed increased *Firmicutes, Verrucomicrobia, and Actinobacteria*. (Fig. 3C–E).

### Fiber-deprived diet causes the expansion of mucus-degrading bacteria

**Fig. 4:**
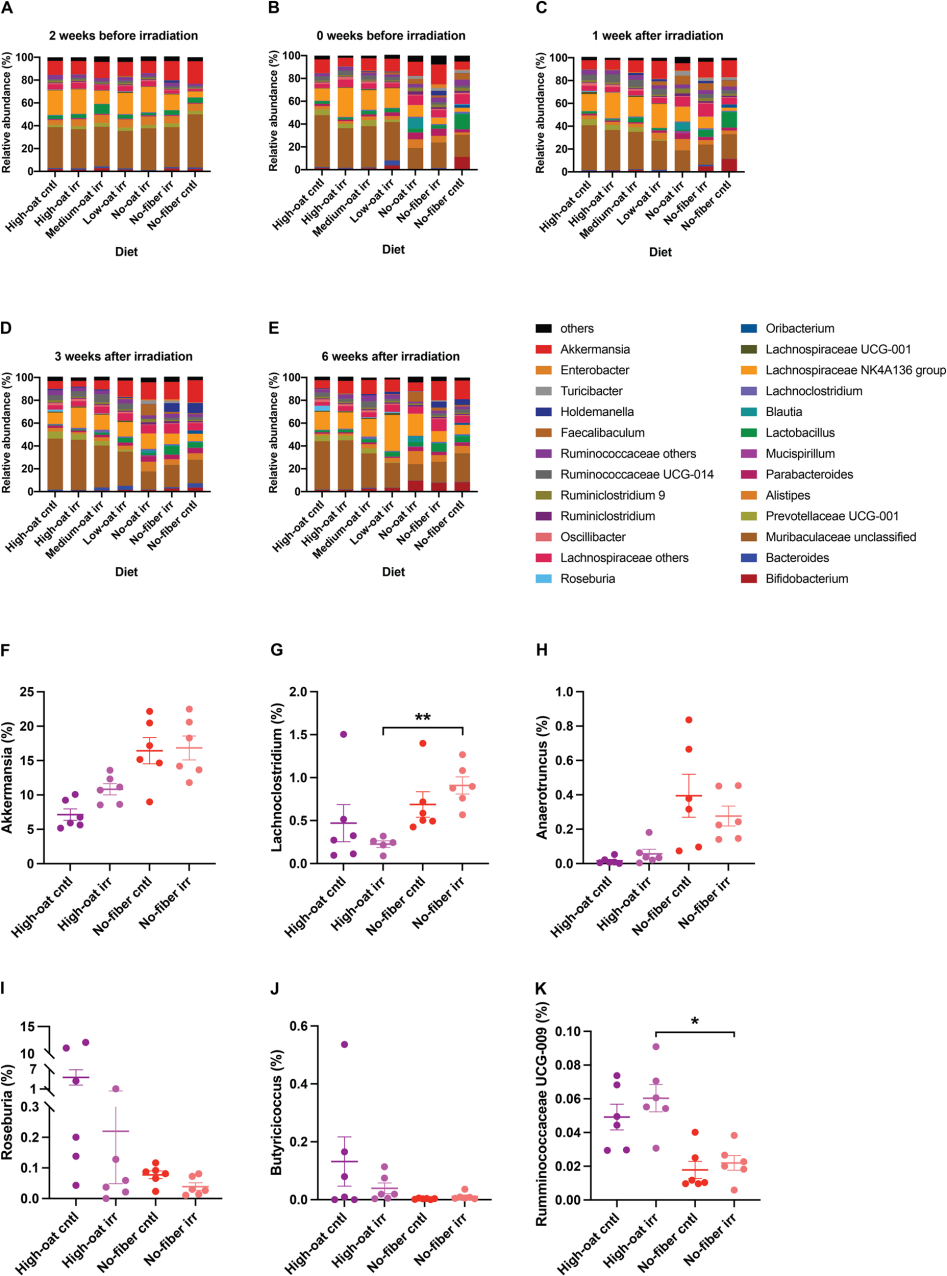
Changes in the relative bacterial abundances over time between the different groups at the OTU level. The mean relative abundances (%) of the bacterial taxa were determined at the OTU level in the different groups, at 2 weeks (**A**) and 0 weeks (**B**) before irradiation, and at 1 week (**C**), 3 weeks (**D**), and 6 weeks (**E**) after irradiation; *n* = 6 mice/group at each time point. The relative abundance of mucus-degrading bacteria, including *Akkermansia* (**F**)*, Lachnoclostridium* (**G**), and *Anaerotruncus* (**H**), was compared between the High-oat and No-fiber groups at 6 weeks after irradiation. The relative abundance of butyrate-producing bacteria, including *Roseburia* (**I**), *Butyricicoccus* (**J**), and *Ruminococcaceae UCG-009* (**K**), was compared between the high-oat and no-fiber groups at 6 weeks after irradiation.

The OTU profiles were similar between the different groups at the start of the experiment (2 weeks before irradiation), except for the Medium-oat irr group, which had a high abundance of *Lactobacillus*, and the No-fiber cntl group, which had a high abundance of *Muribaculaceae*, a dominant bacterial group in the mouse gut (Fig. 4A and Supplementary Fig. 1). At 2 weeks after starting the diets (0 weeks before irradiation), mice fed oat bran-containing diets (high-, medium-, and low-oat) showed fewer microbiota changes than those on oat bran-deprived diets (no-oat and no-fiber). Fermentable oat bran diets promoted expansion of commensal gut bacteria, such as *Muribaculaceae*, and fiber-degrading bacteria, *Prevotellaceae UCG-001*, while oat bran-deprived diets led to lower abundances of these bacteria and increased abundance of *Parabacteroides* and *Lachnospiraceae* (Fig. 4B and Supplementary Fig. 1). At 1, 3, and 6 weeks after irradiation, mice on oat bran-deprived diets exhibited an expansion of *Bifidobacterium* and *Alistipes*, along with a reduction in *Ruminococcaceae*, compared to those on oat bran-containing diets (Fig. 4C–E). Mice on a no-fiber diet had higher levels of mucus-degrading bacteria, including *Akkermansia, Lachnoclostridium*, and *Anaerotruncus*, than those on a high-oat diet (Fig. 4F–H).

### Irradiation may prevent the expansion of SCFA-producing bacteria

Mice on the high-oat diet had a higher abundance of butyrate-producing bacteria, such as *Roseburia*, *Butyricicoccus*, and *Ruminococcaceae UCG-009*, than those on a No-fiber diet (Fig. 4I–K). Irradiation appeared to prevent the expansion of *Roseburia*, however, the sample size was too small to confirm a definitive effect.

### Fiber-deprived diet promotes bacteria associated with radiation injury

**Fig. 5:**
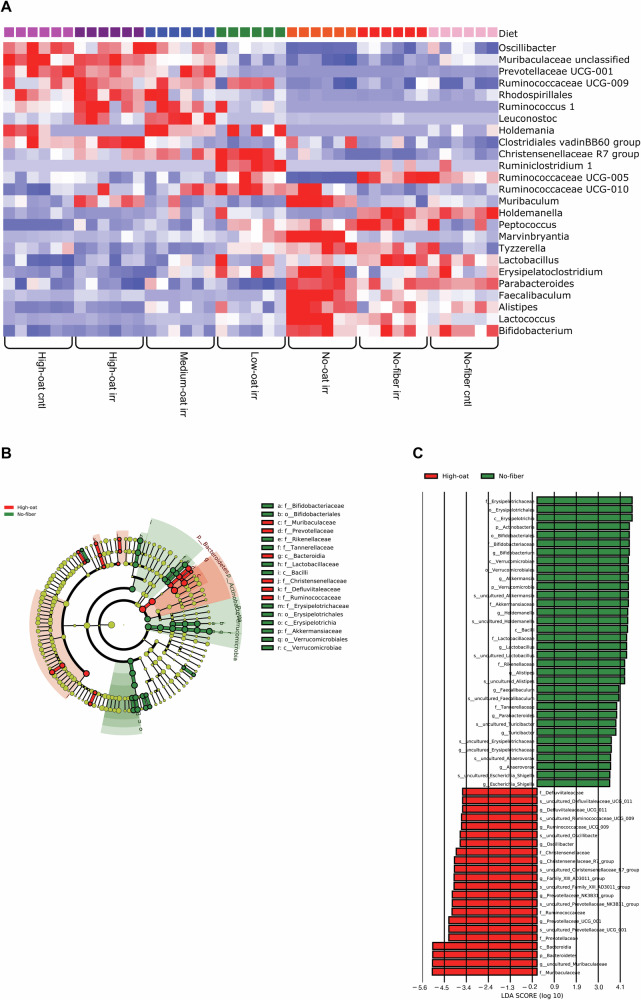
Relative bacterial abundances at 6 weeks after irradiation. A heat map showing OTU abundances in fecal samples from different diet groups was generated using Qlucore Omics Explorer (**A**). OTUs were compared using one-way ANOVA, filtered by a *q* value ≤0.001, and displayed with red (high) and blue (low) abundance; *n* = 6 mice/group. A LEfSe taxonomic cladogram highlighting differentially abundant taxa between the high-oat irr (red) and no-fiber irr (green) groups is shown (**B**). An LDA histogram was generated using LEfSe analysis to identify differentially abundant taxa between the high-oat irr and no-fiber irr groups (**C**); *n* = 6 mice/group.

The differentially abundant bacteria within different groups were identified at 6 weeks after irradiation. Qlucore omics explorer software was used to generate a heat map containing statistically significant bacteria between the different groups (Fig. 5A). Mice on fermentable oat bran diets had significantly higher levels of butyrate-producing bacteria *Ruminococcaceae UCG-009*, while oat bran-deprived diets had significantly higher levels of *Erysipelatoclostridium*, which is linked to radiation-induced intestinal injury^13^. Additionally, *Parabacteroides* and *Alistipes*, associated with harmful effects of irradiation, were more abundant in the oat bran-deprived groups^14^.

### Fiber-deprived diet causes the expansion of pathobionts

To compare gut microbiota between the most contrasting dietary groups at 6 weeks post-irradiation, an LEfSe cladogram and LDA score histogram were generated (Fig. 5B, C). The High-oat irr mice had a higher abundance of commensal bacteria, such as *Muribaculaceae* (LDA = 5.096, *p* = 0.003), *Prevotellaceae UCG-001* (LDA = 4.294, *p* = 0.003), *Christensenellaceae R7 group* (LDA = 4.021, *p* = 0.003), and *Prevotellaceae NK3B31 group* (LDA = 4.127, *p* = 0.002). In contrast, the no-fiber irr mice had a high abundance of potential pathobionts associated with gut dysbiosis, such as *Escherichia* (LDA = 3.531, *p* = 0.022), *Parabacteroides* (LDA = 3.872, *p* = 0.006), and *Turicibacter* (LDA = 3.832, *p* = 0.013), as well as higher levels of mucus-degrading bacteria *Akkermansia* (LDA = 4.442, *p* = 0.01). These results indicate that *Escherichia*, *Parabacteroides*, *Turicibacter*, and *Akkermansia* might contribute to the tissue damage seen in the fiber-deprived mucosa following radiation exposure.

Species diversity was measured by Shannon and Simpson alpha-diversity indices. For both these indexes, after starting the dietary regimen, mice fed the High-oat diet had lower alpha-diversity values than the mice fed the No-fiber diet (Supplementary Fig. 2A, B).

### Irradiation causes decreased production of SCFAs

**Fig. 6:**
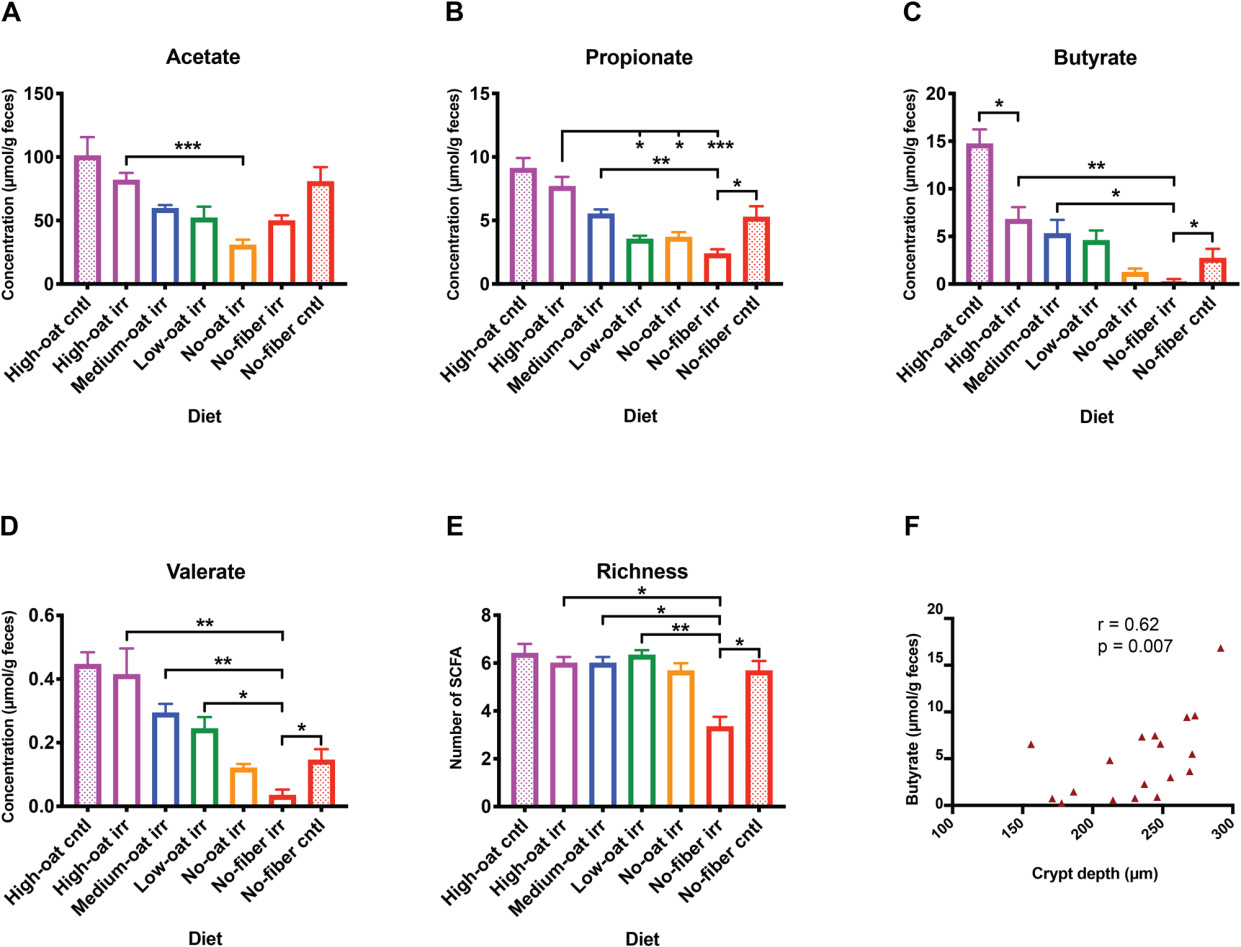
Short-chain fatty acids (SCFAs) analyzed 6 weeks after irradiation using gas-liquid chromatography. SCFAs such as acetate (**A**), propionate (**B**), butyrate (**C**), and valerate (**D**) were measured using gas-liquid chromatography. SCFA richness was quantified by counting the different SCFAs observed in each animal (**E**); *n* = 6 mice/group, except for *n* = 5 in the high-oat cntl group; *p ≤ 0.05, **p ≤ 0.01, ***p ≤ 0.001. Correlation analysis between crypt depth and butyrate at 6 weeks after irradiation (**F**).

The concentrations of SCFAs decreased with decreasing proportions of fermentable oat bran fiber (Fig. 6A–D). Irradiation reduced propionate levels in the No-fiber irr group compared to the No-fiber cntl group (*p* < 0.05) (Fig. 6B). It also lowered butyrate levels in both High-oat irr and No-fiber irr groups compared to their controls (*p* < 0.05) (Fig. 6C). Additionally, valerate levels were significantly lower in the no-fiber irr group than in the no-fiber cntl group (*p* < 0.05) (Fig. 6D).

SCFA richness was determined by quantifying the number of different SCFAs in fecal samples collected from each mouse. The no-fiber irr group showed significantly lower SCFA richness than the high-oat irr, medium-oat irr, and low-oat irr groups (*p* < 0.05, *p* < 0.05, and *p* < 0.01, respectively) and also compared to the no-fiber cntl group (*p* < 0.05) (Fig. 6E). A correlation analysis showed a positive association between crypt depth and butyrate levels (*r* = 0.62, *p* = 0.007) (Fig. 6F).

Taken together, these findings demonstrate that irradiation reduces SCFA production and the diversity of SCFAs produced by the gut microbiota in a diet-dependent manner. Promoting SCFA production through oat bran intake appears to provide radioprotection to mucosal architecture.

### Irradiation alters the balance from mucus production to its degradation

**Fig. 7:**
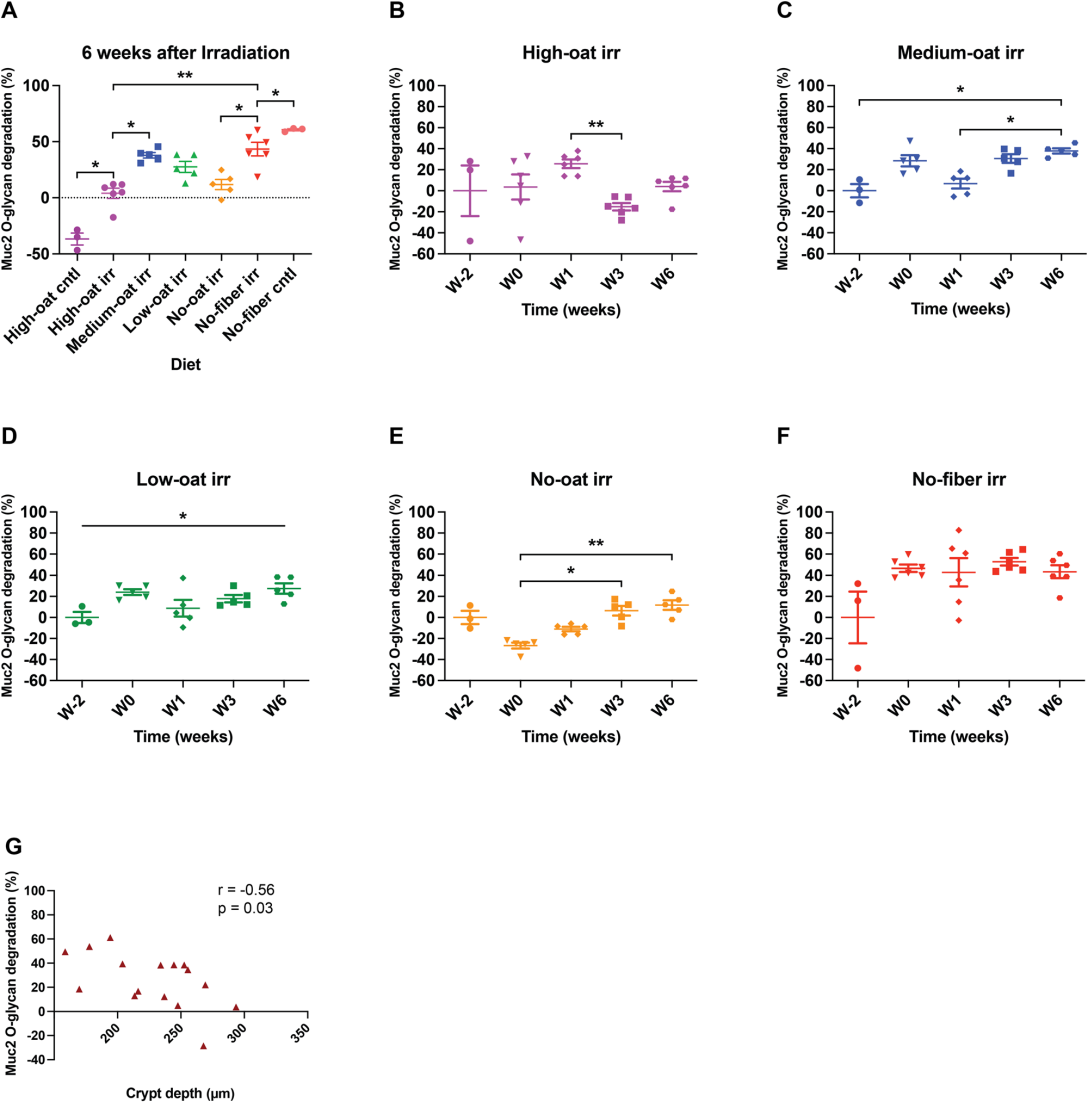
Muc2 O-glycan degradation over time in various dietary groups. The percentage of Muc2 O-glycan degradation at 6 weeks post-irradiation was measured using LC-ESI-MS/MS (**A**), based on the 23 most abundant Muc2 O-glycan structures (shown in Supplementary Fig. 4) in each treatment group versus their baseline. The percentages of Muc2 O-glycan degradation over time for the irradiated groups – high-oat irr (**B**), medium-oat irr (**C**), low-oat irr (**D**), no-oat irr (**E**), and no-fiber irr (**F**) are shown. *p ≤ 0.05, **p ≤ 0.01. Correlation analysis between crypt depth and mucus degradation at 6 weeks after irradiation (**G**).

Diet modifies the gut microbiota composition and activity, directly influencing the extent to which bacteria degrade mucus in their quest for nutrients. To quantify mucus degradation across groups, fecal Muc2 *O*-glycan degradation activity was analyzed at 6 weeks post-irradiation and compared to baseline levels before the experimental diets (Fig. 7A). Mice on the No-fiber diet exhibited the highest Muc2 degradation activity, with No-fiber cntl mice showing a 60% increase in Muc2 *O*-glycan degradation compared to baseline, with a slightly smaller increase in the no-fiber irr mice. In contrast, high-oat cntl mice experienced a 37% decrease in Muc2 *O*-glycan degradation activity from baseline. Irradiation completely blocked this reduction; instead, high-oat irr mice showed a 4% increase in Muc2 *O*-glycan degradation. However, the High-oat irr group still exhibited significantly lower Muc2 *O*-glycan degradation activity compared to the medium-oat irr and no-fiber irr groups (*p* < 0.05 and *p* < 0.01, respectively).

To get a deeper insight into how irradiation affected Muc2 O-glycan degradation, the irradiated groups were followed over time (Fig. 7B–F). One week post-irradiation, the high-oat irr group showed high Muc2 *O*-glycan degradation compared to baseline, which transiently decreased at week 3 (*p* < 0.01), before returning to baseline level by week 6 (Fig. 7B). This suggests that irradiation blocks the shift from mucus degradation to mucus production triggered by fermentable fiber intake, rather than directly increasing degradation. In contrast, the medium-, low-, and no-oat irr groups showed rising Muc2 *O*-glycan degradation over time (Fig. 7C; medium-oat irr, *p* < 0.05 for both comparisons, Fig. 7D; low-oat irr, *p* < 0.05, Fig. 7E; no-oat irr, *p* < 0.05 and *p* < 0.01, respectively). The No-fiber irr group had consistently high Muc2 degradation, with a ∼45% increase two weeks into the diet (Fig. 7F).

Muc2 is heavily glycosylated and has terminal modifications such as sialylation, fucosylation, and sulfation, which create a barrier against degradation by the microbiota. To investigate why the High-oat irr and No-oat irr groups had the lowest mucus degradation activity among irradiated groups at 6 weeks after irradiation, we analyzed nine sialylated Muc2 *O*-glycan structures with one or two *N*-acetylneuraminic acid (Neu5Ac) residues (Supplementary Fig. 4). It was found that these groups had the lowest levels of desialylation (loss of sialic acid residue; 15 and 9%, respectively) compared to other irradiated groups, which lost about 25% of their sialylated structures (Supplementary Fig. 5). The No-fiber irradiated mice had an expansion of sialic acid-catabolizing bacteria, such as *Lactobacillus* and *Escherichia* compared to the High-oat irradiated mice (Fig. 5A, C).

A correlation analysis revealed a negative association between crypt depth and mucus degradation (*r* = −0.5551, *p* = 0.0256) (Fig. 7G). Combined with the positive correlation observed between butyrate concentration and crypt depth (Fig. 6F), these data support the hypothesis that balancing mucus production and degradation through dietary interventions may offer radioprotection.

### Oat bran diet stimulates bacterial host defense

**Fig. 8:**
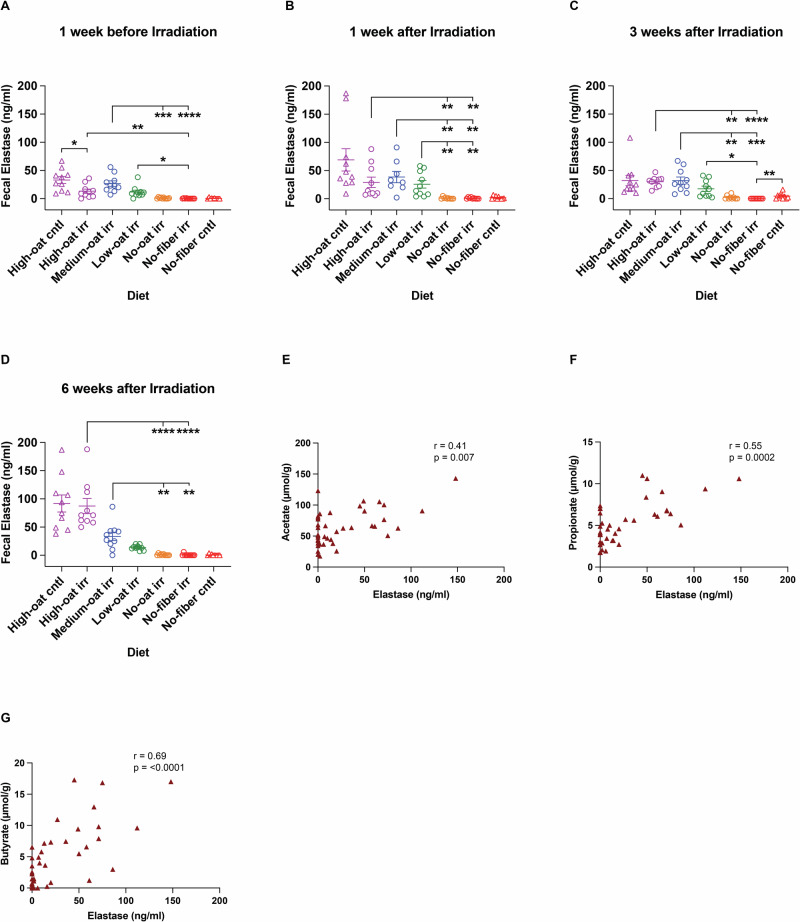
Fecal neutrophil elastase concentrations detected in mice fed the different diets over time. The concentrations of neutrophil elastase in the fecal samples were measured at 1 week before irradiation (**A**) and at 1 week (**B**), 3 weeks (**C**), and 6 weeks (**D**) after irradiation. Correlation analysis between elastase and the SCFAs acetate (**E**), propionate (**F**), and butyrate (**G**) was performed at 6 weeks after irradiation; *n* = 10 mice/group; *p ≤ 0.05, **p ≤ 0.01, ***p ≤ 0.001, ****p ≤ 0.0001.

We previously showed in mice that fermentable oat bran fiber blocks irradiation-induced bacterial infiltration^12^. While this effect is likely attributable to the prebiotic effects of oat bran fiber on mucus barrier dynamics as shown in this study, additional mechanisms may also contribute. An effective complement to the mucus barrier is the production of antimicrobials by immune cells. Neutrophil elastase has strong antimicrobial activity, through which it increases the host defense against invading pathogens^15^. We therefore sought to investigate whether diet and/or irradiation influence neutrophil elastase levels. After one week on the experimental diets, the High-oat irr group showed a slightly lower baseline level than its control group (*p* < 0.05); however, this difference diminished over time. The oat bran-containing diets led to significantly higher elastase levels compared to the oat bran-deprived diets, with levels increasing over time (*p* < 0.05 for all the analysis) (Fig. 8A–D). These findings suggest that oat bran enhances neutrophil elastase production in a dose-dependent manner, potentially boosting host defense. We also observed that neutrophil elastase was positively associated with SCFAs, such as acetate (*r* = 0.41, *p* = 0.007), propionate (*r* = 0.55, *p* = 0.0002), and butyrate (*r* = 0.69, *p* < 0.0001) (Fig. 8E–G). These results suggest that fermentation of dietary oat bran fiber increases SCFAs levels in the gut, which may subsequently enhance antimicrobial activity by recruiting neutrophils to the colonic lamina propria and promoting the release of neutrophil elastase.

### Fiber-deprived diet causes increased body weight

Mice body weights were measured at the start (2 weeks pre-irradiation) and end (6 weeks post-irradiation) of the experiment. At 2 weeks before irradiation, mice in all groups were consuming a standard chow diet and had similar body weights (~23 g) (Supplementary Fig. 6A). At 6 weeks post-irradiation, the high-oat irr group had significantly lower body weight than the medium-oat irr, low-oat irr, no-oat irr, and no-fiber irr groups (*p* < 0.01 and *p* < 0.0001 for the last three comparisons, respectively). An effect of irradiation was observed in the high-oat irr group, having significantly lower body weight than the high-oat cntl group (*p* < 0.0001) (Supplementary Fig. 6B). Over the 8-week study, no-fiber diet mice gained the most weight, while high-oat diet mice gained the least (*p* < 0.0001) (Supplementary Fig. 6C).

## Discussion

The health benefits of dietary fiber have been recognized for decades, yet individuals undergoing pelvic radiotherapy are commonly advised to reduce their fiber intake. The advice aims to alleviate additional bowel discomfort produced by the fermentation of dietary fiber, but there is limited knowledge of how this advice may impact long-term bowel health in irradiated cancer survivors. Currently, there is insufficient scientific evidence to support the development of specific dietary guidelines for cancer patients undergoing pelvic radiotherapy, yet such guidelines are greatly needed. In this study, we used a mouse model of pelvic radiotherapy to assess how dietary fiber intake and dietary fibers of varying fermentability affect key factors in barrier defense dynamics and radiation-induced mucosal injury. Two different dietary fibers—oat bran and microcrystalline cellulose (MCC)—were used in varying proportions. Oat bran, which is readily fermentable, was enzymatically bioprocessed to enhance its palatability and digestibility. In contrast, MCC is poorly fermentable in both humans and rodents and exhibits limited prebiotic activity^16,17^. Our findings show that radiation exposure disrupts the balance between mucus production and degradation, and that the type of dietary fiber consumed during and after irradiation profoundly influences the magnitude of this disruption. Radiation exposure resulted in decreased levels of SCFAs, especially butyrate, while simultaneously increasing mucus degradation activity. A high intake of oat bran fiber alleviated radiation-induced toxicity by increasing the production of SCFAs, reducing the abundance of dysbiotic bacteria, decreasing mucus degradation, and enhancing host defense. Together, these likely contributed to the radioresilience of the mucosa in mice consuming fermentable oat bran fiber. In contrast, a fiber-deprived diet led to a decreased production of SCFAs, a high abundance of dysbiotic bacteria, increased mucus degradation and decreased host defense. Additionally, a fiber-deprived diet disrupted gut homeostasis and was accompanied by profound intestinal tissue damage in both irradiated and sham-irradiated mice.

Diet can rapidly alter the gut microbiota composition in both humans and mice^18,19^, making dietary fiber a powerful tool for rapidly targeting the microbiota in various disease contexts. Dietary fiber alters the niche environment in the gut by providing the substrates required by microbial species for their growth^20^. The concentrated oat bran used in our study contains 52% fiber, of which 28% corresponds to β-glucan. β-glucan, a water-soluble fiber in oats, is known to lower insulin resistance, reduce cholesterol, and decrease heart disease risk by influencing gut microbiota and increasing SCFA production^21–23^. Additionally, oat bran contains bioactive compounds like phenolic acids and avenanthramides, which have anti-inflammatory and antioxidant properties^24–26^. Enzymatic bioprocessing of cereals has been shown to improve its bioavailability and nutritional properties^27^. In this study, the bioprocessing results in a drinkable porridge with enhanced palatability, which could facilitate intake during cancer treatment.

A large variety of bacteria can ferment β-glucan from oats, leading to their expansion. On the level of bacterial phyla, we found that mice fed high proportions of the oat bran (high-oat diet) had a higher abundance of *Bacteroidetes* compared to those on a fiber-deprived diet (no-fiber diet), and a slight reduction of the phylum *Firmicutes*. The expansion of *Bacteroidetes* following a high-fiber diet is not unique to mice; children in rural Burkina Faso who consumed a high-fiber diet had more *Bacteroidetes* compared to urban children in Italy, who had more of the phyla *Firmicutes*. The increase in Bacteroidetes was likely related to their possession of numerous genes encoding carbohydrate-active enzymes (CAZymes), which are essential for breaking down dietary plant polysaccharides^28^. Moreover, children in Burkina Faso had a high abundance of the genus *Prevotella*, which also efficiently breaks down dietary fibers, and a low abundance of the potentially highly dysbiotic genus *Enterobacteriaceae*. Our mice on a fiber-rich diet showed a similar pattern, suggesting that several of the diet-induced changes in our mouse model are relevant to humans as well^29^.

Dysbiosis may significantly contribute to radiation enteropathy, and individuals undergoing radiotherapy often show a progressively increasing dysbiotic pattern that persists post-treatment^30^. The irradiated mice on a fiber-deprived diet exhibited an expansion of *Firmicutes* and a reduction in *Bacteriodetes*. A high *Firmicutes* to *Bacteroidetes* (F/B) ratio is often considered a marker of gut microbiota dysbiosis or imbalance, although this interpretation is not universally applicable. The fiber-deprived mice also had higher levels of dysbiotic bacteria, such as *Escherichia* and *Parabacteroides*, compared to the high-oat irradiated mice. Notably, these bacteria are also significantly more abundant in the gut microbiota of individuals with colorectal cancer^31^.

We found that the type and proportions of dietary fiber intake were much stronger predictors of microbial composition than irradiation. Both irradiated and sham-irradiated animals fed either high amounts of poorly fermentable microcrystalline cellulose or a no-fiber diet exhibited profound disruptions in fecal bacterial composition over the course of the experiment. In contrast, the relative abundance of various phyla in groups consuming high proportions of fermentable oat bran fiber remained relatively constant over time, showing only a slight increase in Bacteroidetes and stable colonization of Firmicutes, regardless of irradiation. This supports the idea that an adequate intake of fermentable fiber seems to be needed to stabilize the microbiota against potential disruptions, shaping it into a profile that enhances barrier function and promotes overall bowel health after radiotherapy.

Irradiation reduced SCFA production and the diversity of SCFAs produced by the gut microbiota in a diet-dependent manner. SCFAs, and in particular butyrate, serve as an energy source for colonocytes and play a key role in regulating energy homeostasis in the colon^32^. As expected, mice consuming a diet with a higher proportion of fermentable oat bran fiber exhibited higher fecal butyrate levels compared to those on a fiber-deprived diet. Butyrate is known for its anti-inflammatory and anti-carcinogenic properties, as well as its ability to restore intestinal barrier function, regulate colonic mucosal homeostasis, and modulate intestinal motility. Moreover, butyrate enhances intestinal barrier function by reducing epithelial permeability and increasing transepithelial resistance through the regulation of tight junction assembly; it also stimulates Muc2 expression in intestinal epithelial cells^33–35^. The increase in butyrate in the mice consuming fermentable oat bran fiber was associated with an expansion of butyrate-producing bacteria, such as *Roseburia*, *Butyricicoccus* and *Ruminococcaceae UCG-009*. *Roseburia* is linked to gut health and is less abundant in individuals with colorectal cancer^31^. In a study by Ferreira et al., the abundance of *Roseburia* decreased significantly over time in individuals subjected to pelvic radiotherapy, supporting the notion that this genus is particularly sensitive to irradiation^36^. In our study, irradiation prevented the expansion of *Roseburia* in mice fed a diet with a higher proportion of oat bran, which may have contributed to the reduced butyrate levels observed in these animals. Moreover, our data revealed a decrease in *Roseburia* and *Butyricicoccus* and an increase in *Enterobacteriaceae* in the irradiated fiber-deprived mice. It is known that the depletion of these beneficial bacteria allows for the expansion of dysbiotic *Enterobacteriaceae* through microbiota-activated peroxisome proliferator-activated receptor gamma (PPARγ) signaling.^37^.

The colonic mucosa is protected by two mucus layers that create a barrier separating bacteria in the lumen from the epithelium^10^. Diet and gut microbiota play important roles in maintaining the normal structure and production of the mucus. A fiber-deprived diet alters the gut microbiota and increases levels of mucus-degrading bacteria, thereby significantly weakening the mucus barrier and raising the risk of infections and chronic diseases^38,39^. We recently reported that the mucus barrier in irradiated pelvic cancer survivors is either nearly absent or of poor quality, allowing bacteria to infiltrate the underlying mucosa^11^. Therefore, possible changes in Muc2 O-glycan degradation activity were measured in the various experimental groups. Mice consuming a fiber-deprived diet exhibited increased Muc2 O-glycan degradation compared to those on a diet with higher proportions of fermentable oat bran fiber. The fiber-deprived mice also showed a higher abundance of *Akkermansia* and other mucus-degrading bacteria. In contrast, mice consuming a fiber-rich oat bran diet showed an expansion of bacterial species specializing in fiber degradation, while species that specialize in mucus degradation, such as *Akkermansia*, were reduced. This shift occurs because oat bran provides abundant fermentable substrates for fiber-degrading bacteria, thereby reducing the need for microbes to rely on host-derived mucins as an energy source. The high-oat irradiated mice also had a significantly higher abundance of *Bacteroides* compared to the no-fiber irradiated mice. Some *Bacteroides* species are known to boost mucus production by enhancing the expression of mucus-related genes^40^. Dietary fibers also mechanically stimulate the intestinal mucosa to secrete mucus^41^, suggesting that the High-oat diet reduces mucus degradation and enhances mucus production, thereby improving gut barrier function. In contrast, the no-fiber diet promotes mucus degradation. One of the first steps in colonic mucus degradation is desialylation of glycans, where sialic acid is removed by sialidase enzymes^42^. In line with the previous study, our mice on a fiber-deprived diet exhibited higher desialylation levels compared to those on a fermentable oat bran diet, likely due to the greater abundance of sialic acid-catabolizing bacteria such as *Escherichia* and *Lactobacillus*^43^. Irradiation triggered increased Muc2 *O*-glycan degradation in mice on the oat bran diet, although it did not reach the levels seen in fiber-deprived animals. Along with the observation that irradiation decreases butyrate production in both fiber-rich and fiber-deprived groups, our data indicate that irradiation shifts the gut microenvironment in a manner that favors Muc2 *O*-glycan degradation over the production of short-chain fatty acids. This shift toward mucus degradation may contribute to the persistent loss of mucus barrier protection observed in irradiated pelvic cancer survivors and warrants further investigation.

Neutrophils are key components of the innate immune system that guard the host mucosa against invading pathogens, and neutrophil elastase is part of the host’s defense against gram-negative bacteria in particular^15^. Mice fed the oat bran-supplemented diets produced fecal neutrophil elastase in a concentration-dependent manner. In contrast, mice fed the oat bran-deprived diets had undetectable or nearly undetectable levels of fecal neutrophil elastase. Since we have previously shown that male C57BL/6 mice fed the oat bran-supplemented diet exhibit lower levels of pro-inflammatory serum cytokines compared to those deprived of oat bran^44^, we conclude that the increase in neutrophil elastase reflects enhanced bactericidal activity rather than increased mucosal inflammation. A possible mechanism behind the stimulation of bactericidal activity in fiber-consuming animals is the enhanced production of SCFAs, which have been shown to attract both human and murine neutrophils through G protein-coupled receptor 43 activation^45,46^. In line with this, neutrophil elastase concentrations showed positive correlations with all three major SCFAs, exhibiting a particularly strong correlation with butyrate. Butyrate is known to increase the recruitment of neutrophils to the colonic lamina propria, which may subsequently enhance antimicrobial activity through the release of neutrophil elastase^47^.

Dietary fiber consumption has a profound impact on weight in both humans and animals, influencing multiple physiological and metabolic factors. Mice on the No-fiber diet had the highest weight among all groups, likely in part due to a greater abundance of *Firmicutes* and lower levels of *Bacteroidetes* in their gut microbiota, as observed in obese C57BL/6 J mice^48^. In contrast, High-oat irradiated mice exhibited significantly lower body weight, possibly due to a higher abundance of the *Christensenellaceae R7 group*, which is linked to lean individuals and reduced weight gain^49^.

We have previously shown that pelvic radiotherapy leads to permanently smaller crypts in pelvic cancer survivors, while other studies have demonstrated that irradiation results in crypt shortening in C57BL/6 mice^11,50^. Therefore, we measured the average intestinal crypt depth across different treatment groups to assess the impact of dietary fiber and irradiation on normal mucosal architecture. Our findings revealed that the amount of fermentable oat bran fiber had a significant impact on crypt depth, which increased progressively with higher proportions of oat bran fiber. Similar findings were reported in a study where rats on a fiber-rich diet showed increased colonic crypt depth than those on an elemental diet^51^. Mice fed a fiber-deprived diet exhibited crypts that were less than half the depth of those in mice fed a high-oat bran fiber diet. Furthermore, crypt depth correlated positively with increasing levels of SCFAs and inversely with Muc2 degradation. Lastly, the detrimental effect of irradiation was more pronounced in the No-fiber irradiated group compared to the High-oat irradiated group, as evidenced by significantly reduced crypt depth—an indicator of intestinal tissue damage. These results further support the hypothesis that modifying mucus dynamics and mucosal barrier function through dietary fiber interventions may offer radioprotection.

This study has some limitations. Mice are known to be relatively resistant to radiation compared to humans, particularly in terms of intestinal injury, which may result in an underestimation of the severity of radiation-induced damage observed in clinical settings. Additionally, many symptoms of radiation-induced intestinal injury observed in humans, such as diarrhea, fecal urgency, or incontinence, are either absent or difficult to measure in mice. This limits our ability to predict the effects of dietary interventions or other treatments on symptoms experienced by individuals exposed to radiation. While the fiber-rich oat bran diet proved to be highly effective in reducing radiation-induced toxicity in this study, the specific bioactive component(s) responsible for the various beneficial effects remain unknown, necessitating further mechanistic investigations. Another limitation of this study is the exclusion of female mice, due to the significant risk of exposing the lower reproductive tract to radiation. Such exposure could lead to hormonal disruptions, potentially confounding the interpretation of our results. Investigating the effects of irradiation in female mice would be an important direction for future studies.

In conclusion, the amount and type of dietary fiber consumed in relation to radiation exposure play a key role in several mechanisms that protect the mucosal barrier following radiotherapy. Our findings highlight the critical role of fermentable dietary fiber intake in enhancing mucosal resilience to radiation-induced damage. These results suggest that fermentable dietary fiber could help mitigate radiation-induced toxicity in individuals undergoing pelvic radiotherapy, emphasizing the importance of developing evidence-based dietary guidelines for cancer patients receiving this treatment. Based on the evidence presented in this study, there is a strong rationale for conducting well-designed randomized controlled trials to evaluate the effects of different types and quantities of dietary fiber on radiation-induced toxicity and overall treatment outcomes in individuals undergoing pelvic radiotherapy.

## Methods

### Ethics statement

All the animal experiments were performed with the approval of the Gothenburg Committee of the Swedish Animal Welfare Agency (Ethics permit no 1458-2018).

### Mice

Male C57BL/6 mice were purchased from Charles River Laboratories (Sulzfeld, Germany) and were used in all the experiments. Male mice were chosen to minimize variability, since the placement of the radiation field over the colorectum may also irradiate the lower organs of the reproductive system in females. Mice were housed in groups of five on wood chip bedding in individually ventilated Greenline cages (GM500, Techniplast, Varese, Italy) and were allowed at least 2 weeks to acclimate before the start of the experiments. During this acclimation period, mice had ad libitum access to water and standard chow (Teklad Global 16% Protein Rodent Diet, Teklad Diets, Madison, WI). Prior to the initiation of the experiments, enrichment items such as shredded paper, toilet paper rolls, and wood sticks were removed. The dietary intervention began when the mice were 9 weeks old, an age at which the mouse gastrointestinal system has reached both structural and functional maturity^52,53^. All the animals were housed at a constant temperature (20 °C) with 42% relative humidity. A 12-h light/dark cycle was maintained with unlimited access to food and water.

### Experimental design

**Fig. 9:**
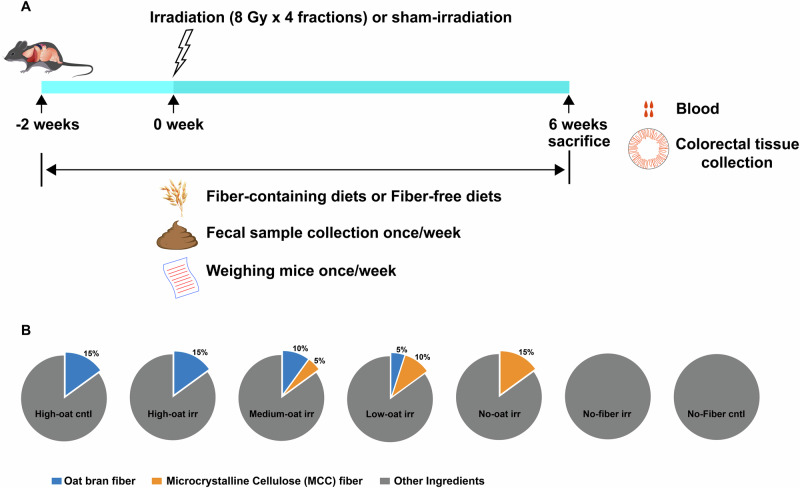
Experimental design. Seventy mice were divided into seven groups, with ten mice in each group, and were assigned to five different diets starting 2 weeks before irradiation and continuing until 6 weeks after irradiation (**A**). Body weights and fecal samples were collected weekly. The compositions of the diets are shown in (**B**). The diets contained either 15% total fiber, with counterbalanced proportions of bioprocessed oat bran fiber and/or microcrystalline cellulose fiber, or 0% fiber. The detailed composition of the diets is provided in Table 1 and Supplementary Table 2.

Seventy mice were divided into seven groups (ten per group). Five out of seven groups were irradiated (irr) and fed different diets starting 2 weeks before irradiation until 6 weeks post-irradiation (Fig. 9A). The diets contained either 15% fiber or 0% fiber. The diets that contained 15% fiber had varying proportions of readily fermentable bioprocessed oat bran (oat) fiber and/or poorly fermentable microcrystalline cellulose (MCC) fiber, combined in a counterbalanced manner. To ensure all diets were isocaloric, we adjusted the formulations with purified corn starch. The irradiated groups were high-oat irr (15% bioprocessed oat bran fiber), medium-oat irr (10% bioprocessed oat bran fiber + 5% MCC fiber), low-oat irr (5% bioprocessed oat bran fiber + 10% MCC fiber), no-oat irr (15% MCC fiber) and no-fiber irr (0% Fiber) (Fig. 9B). The dietary compositions and the total dietary fiber contents of these diets are listed in Table 1, and the composition of the basal mixture is listed in Supplementary Table 2. Two non-irradiated groups were fed the same diet as the high-oat irr and no-fiber irr groups, served as their respective controls (high-oat cntl and no-fiber cntl). The oat bran was kindly provided by Glucanova AB (Lund, Sweden), and the bioprocessed liquid oat bran was prepared according to the European patent # 2996492^54^. MCC (Avicel PH-101) was purchased from FMC International (Cork, Ireland), and the basal mixture diet (TD.160816) was custom-made by Envigo Teklad Diets (Madison, Wisconsin, USA). The purified corn starch (Cargill’s C*Gel 03401) was kindly provided by Caldic Ingredients Sweden AB (Malmö, Sweden).

**Table 1:** Dietary composition and dietary fiber content for each group in the study

	Dietary groups						
Ingredient (%)	High-oat cntl	High-oat irr	Medium-oat irr	Low-oat irr	No-oat irr	No-fiber irr	No-fiber cntl
Oat bran (fiber content %)	28.8 (15)	28.8 (15)	19.2 (10)	9.6 (5)	0	0	0
Microcrystalline cellulose (MCC)	0	0	5	10	15	0	0
Corn starch	4.7	4.7	9.3	13.9	18.5	33.5	33.5
Basal mixture	66.5	66.5	66.5	66.5	66.5	66.5	66.5
**Total weight (g)**	**100**	**100**	**100**	**100**	**100**	**100**	**100**

^a^Oat bran contains 52% fiber, of which 28% is beta-glucan. The composition of the basal mixture is given in Supplementary Table 2.

The values in bold indicate the sum of all the ingredients (oat bran, MCC, corn starch, and basal mixture) in grams.

### Irradiation procedure

Mice were anesthetized with isoflurane (Isoba® vet, MSD Animal Health, UK) and placed in a silicone mold. A 3 × 3 cm² radiation field from a linear accelerator (TrueBeam; Varian Medical Systems Inc., Charlottesville, Virginia, USA) targeted 1.5 cm of the colorectum. Four fractions of 8 Gy were delivered with 6 MV photon energy at 5.9 Gy/min, with 12-h intervals between fractions. A 5 mm tissue-equivalent bolus was used to cover the irradiated part of the body to ensure even distribution of irradiation throughout the underlying tissue. The dose variation within the target volume was ±5%, and the procedure took less than 5 min. Control animals were anesthetized under the linear accelerator but not irradiated.

Pelvic radiotherapy in cancer patients typically involves daily fractions of ~2 Gy over multiple weeks or 5 Gy fractions administered daily for 5 consecutive days. Delivering four fractions of 8 Gy, spaced 12 h apart, produced mucosal pathophysiology in mice very similar to that observed in pelvic cancer patients, including crypt cell loss, mucosal degeneration, and fibrosis, without affecting the general health of the mice^55,56^.

### Sample collection

At 6 weeks post-irradiation, mice were anesthetized with isoflurane (Isoba® vet, MSD Animal Health, UK), after which colorectal tissues were excised and fixed in methanol-Carnoy’s solution. The mice were then euthanized by decapitation while still under anesthesia. Fecal samples were collected weekly from each mouse throughout the eight-week experiment and stored at −80 °C for later use. Fecal samples were collected a few hours before the dietary regimens began (−2 weeks time point) and a few hours before the irradiation procedure (0 weeks point).

### Histological analysis

Histology was performed with Verhoeff-Van Gieson staining. Tissue sections (4-μm) were deparaffinized, rehydrated, stained with Verhoeff’s solution for 15 min, and differentiated with 2% ferric chloride. The sections were then treated with 5% sodium thiosulfate and counterstained with Van Gieson’s solution for 20 s, followed by dehydration through an ethanol series, clearing with xylene, and coverslipping with Pertex (Histolab AB, Askim, Sweden).

### Colon crypt depth measurement

The colonic crypt depth was measured using a Leica DM6000B microscope (Leica Microsystems AB, Wetzlar, Germany) equipped with a semi-automated stereology system (Stereo Investigator 6; MBF Bioscience, Williston, Vermont, USA). The depth of all the crypts in each section were measured using the Stereo Investigator's line tool and the average crypt depth was calculated for each mouse.

### Fecal microbiota analyses

Fecal samples were analyzed for microbial diversity at five different time points: 2 and 0 weeks before irradiation and 1, 3, and 6 weeks after irradiation. Bacterial DNA was extracted from fecal samples following the QIAamp DNA stool mini kit (QIAGEN AB, Sollentuna, Sweden), except that the bead beating step was included using glass beads to increase the DNA yield. The fecal microbiota composition was profiled by sequencing the V3–V4 region of the 16S rRNA gene in an Illumina MiSeq system using the primers 341 F (5′-*TCGTCGGCAGCGTCAGATGTGTATAAGAGACAG*CCTACGGGNGGCWGCAG-3′) and 785 R (5′-*GTCTCGTGGGCTCGGAGATGTGTATAAGAGACAG*GACTACHVGGGTATCT AATCC-3′) adopted from Klindworth et al.^57^, designed for dual indexing. The sequences in italics are overhang adapters, followed by primers targeting the V3–V4 region of the 16S rRNA gene. Samples were PCR amplified using 1 × Kappa HiFi HotStart Readymix (KAPA Biosystems, USA) and purified with AmPure XP magnetic beads (Beckman Coulter, USA). Single multiplexing was performed using an 8-bp index, added to both the forward and reverse primers during a second PCR. These primer sequences are listed in Supplementary Table 3. The samples were amplified again using 1 × Kappa HiFi HotStart Readymix, purified with AmPure XP magnetic beads and quantified using the Qubit dsDNA high-sensitivity assay kit. After the second PCR, the amplicon size was determined using Agilent 2200 Tapestation with the DNA screenTape analysis kit (Agilent Technologies Sweden AB, Kista, Sweden) and was found to be around 600 bp. Purified PCR products were diluted to 4 ng/μl and pooled in equal amounts. The pooled samples were then sent to SciLifeLab (Science for Life Laboratory, Stockholm, Sweden) for sequencing the 16S rRNA gene amplicons with the Illumina MiSeq system (RTA version 1.18.54; MCS version 2.5.0.5), 2 × 300 sequencing setup, with the MiSeq reagent kit ver. 3 (Illumina Corp., San Diego, CA, USA). The Bcl to FastQ conversion was performed using bcl2fastq_v2.19.1.403 from the CASAVA software suite. The quality of the run was monitored internally using PhiX control v3 (10% spike-in) (Illumina). MiSeq reads were quality checked with the FastQC ver. 0.11.5 software^58^. Raw sequencing data were processed with QIIME 2^59^. Cutadapt was used to remove the sequencing primers from both the forward and reverse reads^60^. If either the forward or reverse primer could not be detected (allowing for 10% mismatches for the primer search), the read pair was discarded. The resulting trimmed reads were imported into QIIME 2 and subsequently demultiplexed. The quality of the paired reads was visually assessed with QIIME 2, and the sequence trimming and truncation base position values were determined. Exact sequence variants (ESVs) were derived for each sequencing run with the DADA2 plugin^61,62^. The resulting merged ESVs were clustered into 97% OTUs using the vsearch plugin with default settings^63^. Taxonomy assignment was performed using the mapped OTUs to the SILVA (v132) SSU rRNA reference sequence database^64,65^. The SILVA reference set used here contains only the V3–V4 amplicon region bounded by our sequencing primers. The generated OTU table was imported into the PhyloSeq package to calculate the alpha diversity based on the Shannon and Simpson methods for the individual group^66^. The dissimilarities in gut microbiota composition between different groups (β-diversity) were assessed with the Bray-Curtis dissimilarity index and were plotted using principal coordinates analysis (PCoA). The result for the diversities was plotted in the R suite software. Operational taxonomic units (OTUs) having a *q* value (adjusted *p* value) ≤0.001 were filtered, and the heat map was generated using the Qlucore Omics Explorer software (Qlucore AB, Lund, Sweden). The Linear discriminant analysis effect size (LEfSe) tool was used to identify differentially abundant bacterial taxa in the high-oat irr and no-fiber irr groups at 6 weeks post-irradiation, with significance set at an LDA score >3.5^67^.

### Short-chain fatty acids (SCFAs) analysis by gas-liquid chromatography

To determine the concentration of various SCFAs, fecal samples collected six weeks post-irradiation were analyzed using a modified gas-liquid chromatography (GLC) method by Zhao et al.^68^. Samples were thawed and homogenized with water at 4 m/s (Fastprep FP120; Thermo Savant, France). The suspension was adjusted to pH 2–3 using 10 M HCl, then homogenized for 30 s and left at room temperature for 10 min. The mixture was centrifuged for 20 min at 5000 rpm (Mikro 22 R; Hettich GmbH, Germany). The supernatant (180 μl) was transferred to a glass vial for gas chromatography, followed by the addition of 20 μl of an internal standard (7.9 mM 2-ethylbutyrate in 12% v/v formic acid). Analysis was performed using a Perkin-Elmer autosystem gas chromatography with a flame ionization detector (FID) and autosampler (Perkin-Elmer, USA). Helium was the carrier gas at a pressure of 30 kPa (AGA Gas AB, Sweden). A 1 μl sample was injected for the analysis. SCFA peaks were identified based on their retention times using a standard SCFA solution. Concentrations were calculated from the peak area ratios relative to the internal standard and corresponding calibration curves, and are expressed in μmol/g feces. In total, seven SCFAs—acetate, propionate, butyrate, valerate, isobutyrate, isovalerate, and isocaproate—were identified and quantified in the fecal samples of mice. SCFA richness was quantified for each mouse by counting the number of SCFAs detected out of the seven listed above.

### Mouse mucus Muc2 degrading activity analysis by LC-MS/MS

The mucus-degrading activities of enzymes released by gut bacteria were evaluated in fecal samples from different groups. The irradiated groups were analyzed at five time points: 2 and 0 weeks before irradiation, and 1, 3, and 6 weeks after. Control groups were analyzed at two time points: 2 weeks before and 6 weeks after irradiation. Fecal samples were incubated with pre-prepared mouse mucus Muc2 dot blots for 4 h at 37 °C. After washing, the retained Muc2 *O*-glycans were analyzed using liquid chromatography-electrospray ionization tandem mass spectrometry (LC-ESI-MS/MS). The detailed experimental procedure is outlined below.

### Mucus Muc2 dot blot preparation

Mucus (300–500 μl in wet volume) was scraped from the small intestine and colon of three male C57BL/6 mice maintained on the standard chow diet described previously. These were additional mice used specifically for Muc2 blot preparation. Mucosal scrapings were extracted with guanidinium chloride in the presence of a protease inhibitor, followed by reduction and alkylation, as described previously^69^. Solubilized mouse Muc2 (20 μl/well) was transferred to PVDF membranes (Merck Chemicals & Life Science AB, Solna, Sweden) using dot blotting, then stained with Alcian blue and destained with methanol. Excised dots were stored at −20 °C until use.

### Muc2 *O*-glycans elimination and detection by LC-ESI-MS/MS

Fecal samples from different mouse groups were suspended in PBS (30 mg/ml, w/v) and incubated with mice Muc2 dot blots for 4 h at 37 °C. Samples heated at 95 °C for 15 min served as negative controls, while blots without fecal extract served as positive controls. O-glycans on the blots were released by reductive beta-elimination, resuspended in water, and analyzed by LC-ESI-MS/MS^70^.

Oligosaccharides were separated on a 10 cm × 250 µm column packed with 5 µm porous graphite particles (Thermo Scientific, Waltham, WA, USA) and eluted with a linear gradient of 0–40% acetonitrile in 10 mM ammonium bicarbonate over 40 min at a flow rate of 10 μl/min. They were detected using an LTQ linear ion trap mass spectrometer (Thermo Scientific) in negative ion mode with an electrospray voltage of 3.5 kV, a capillary voltage of −33.0 V, and a capillary temperature of 300 °C. Compressed air was used as the sheath gas. Full scan (m/z 380–2000 two microscan, maximum 100 ms, target value of 30,000) was performed, followed by data dependent MS^2^ scans (two microscans, maximum 100 ms, target value of 10,000) with normalized collision energy of 35%. The threshold for MS^2^ was set to 300 counts. Data acquisition and processing were conducted with Xcalibur software 2.0.7 (Thermo Scientific). Glycans were identified from their MS/MS spectra by manual annotation.

The dominant 23 *O*-glycans were selected for comparison between samples (Supplementary Fig. 4), and their area under the curve (AUC) was calculated using the Progenesis QI software (Nonlinear Dynamics, Waters Corp., Milford, MA, USA). The AUC of each structure was normalized to the total AUC of 23 *O*-glycans and expressed as a percentage. The baseline for comparison was set at 2 weeks before irradiation, as all mice were on a standard chow diet and not irradiated. Percentages of Muc2 *O*-glycan degradation at later time points (0 week before irradiation, and 1, 3, and 6 weeks after irradiation) reflected changes due to diet and/or irradiation.

### Fecal neutrophil elastase detection

The concentrations of neutrophil elastase in the fecal samples were determined at four different time points (1 week before irradiation, and 1, 3, and 6 weeks after irradiation). This was done using the PMN-Elastase ELISA kit according to the manufacturer’s instructions (Immundiagnostik, Bensheim, Germany). Optical density was measured at 450 nm using an EMax microplate reader (Molecular Devices Inc., San Jose, CA, USA), with the results expressed in ng/ml.

### Statistical analysis

All statistical analyses were conducted using GraphPad Prism (v8.0, Dotmatics, Boston, MA, USA). One-way ANOVA with Tukey’s post hoc test was used for normally distributed data, while the Kruskal–Wallis method was applied for non-normally distributed data to compare differences between more than two groups. For normally distributed data, a two-tailed *t*-test was used to compare irradiated and control groups with the same diet; otherwise, a Mann–Whitney test was used. The number of animals (*n*) and statistical methods for each experiment are described in detail in the respective figure legends. Statistically significant differences are indicated by asterisks: *p ≤ 0.05, **p ≤ 0.01, ***p ≤ 0.001, and ****p ≤ 0.0001.

## Supplementary information

Supplementary information

Author Checklist – Full

LC-ESI-MS_MS spectra of selected structures

## Data Availability

The datasets generated and/or analyzed during the current study are available in the following repositories: The 16S rRNA sequencing data have been deposited in the NCBI Sequence Read Archive (SRA) database with BioProject identification no PRJNA910312 (Accession no SRX18547975–SRX18548184). Raw mass spectrometry glycomic data is deposited on the GlycoPost website: https://glycopost.glycosmos.org/entry/GPST000247.

## Abbreviations

SCFA: Short-chain fatty acids
Muc2: Mucin-2
MCC: Microcrystalline cellulose
Irr: Irradiated
Cntl: Control
ESV: Exact sequence variants
PCoA: Principal coordinates analysis
OTU: Operational taxonomic unit
LEfSe: Linear discriminant analysis effect size
LDA: Linear discriminant analysis
GLC: Gas-liquid chromatography
AUC: Area under curve
Neu5Ac: N-acetylneuraminic acid
CAZymes: Carbohydrate-active enzymes
PUL: Polysaccharide utilization loci
Gal: Galactose
GlcNAc: N-acetylglucosamine
GalNAc: N-acetylgalactosamine
PPARγ: Peroxisome proliferator-activated receptor gamma
