# Supplementary material for: Oat bran fiber protects against radiation-induced disruption of gut barrier dynamics and mucosal damage
Source: NPJ Biofilms Microbiomes. 2025 Jul 4;11:128. doi: 10.1038/s41522-025-00759-x (PMC12227613; doi:10.1038/s41522-025-00759-x)
Supplement: Supplementary file 1 — Supplementary information [file 41522_2025_759_MOESM1_ESM.pdf]

Supplementary Figure 1. Changes in relative bacterial abundances over time within each group at the OTU level.

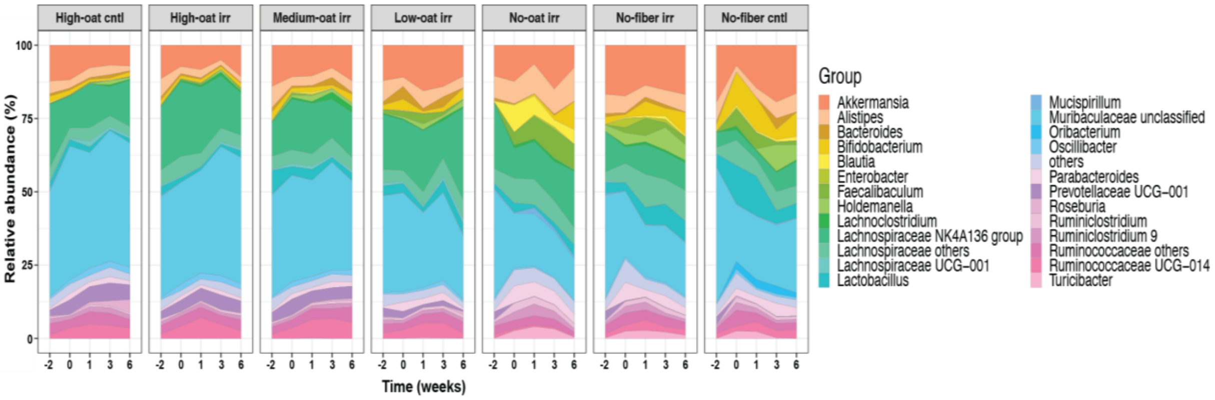

Stack area plot showing the fecal microbial community dynamics and average relative abundances of OTUs in the different groups at 2 and 0 weeks before irradiation, and at 1, 3, and 6 weeks after irradiation; n = 6 mice/group at each time point.

Supplementary Figure 2. Shannon's and Simpson's alpha diversity index.

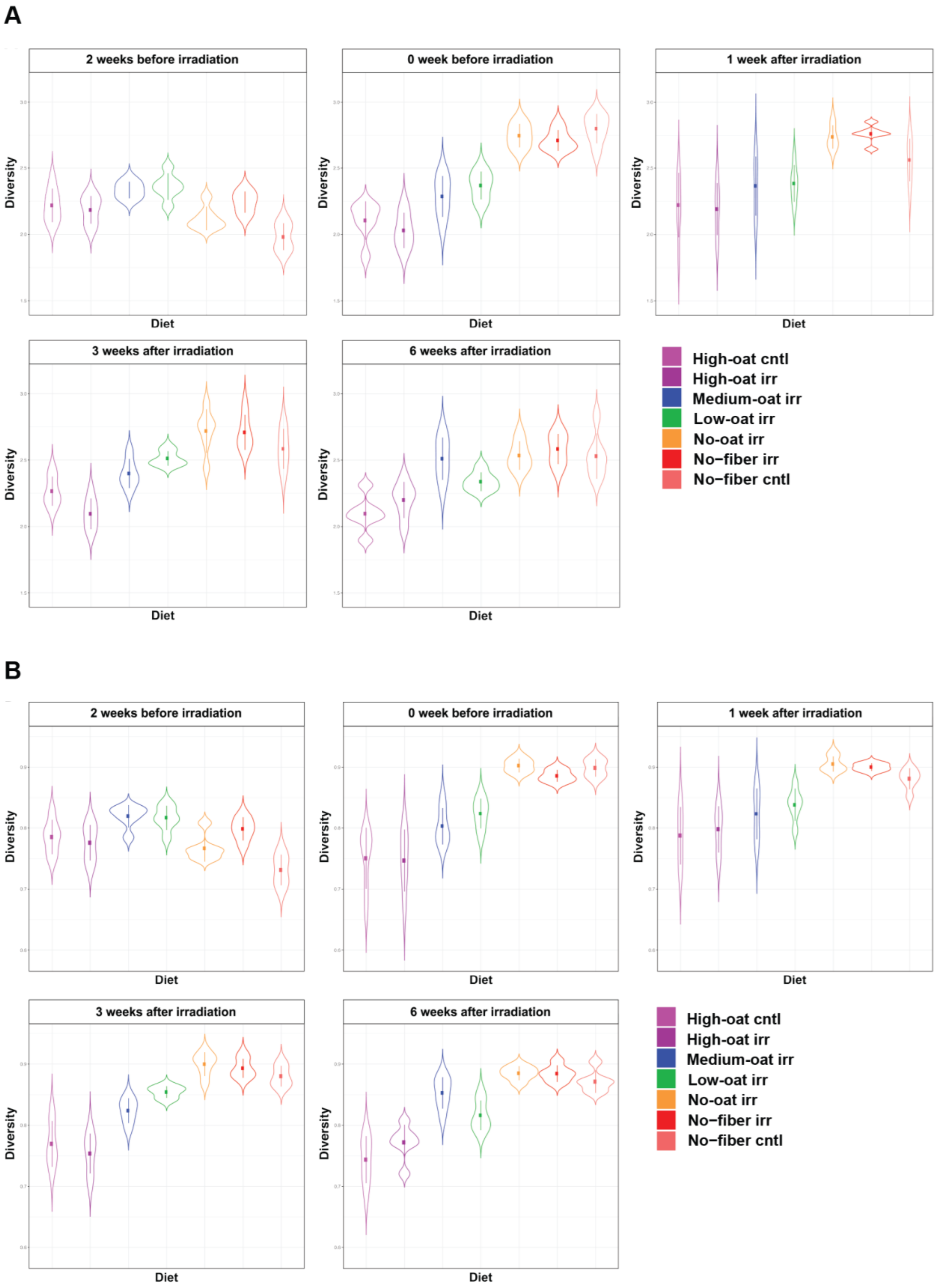

The Shannon (**A**) and Simpson (**B**) alpha-diversity indices for the different groups were plotted at 2 and 0 weeks before irradiation, and at 1, 3, and 6 weeks after irradiation; n = 6 mice/group at each time point.

Supplementary Figure 3. Short-chain fatty acid concentrations in the feces of mice at 6 weeks after irradiation, as measured using gas-liquid chromatography.

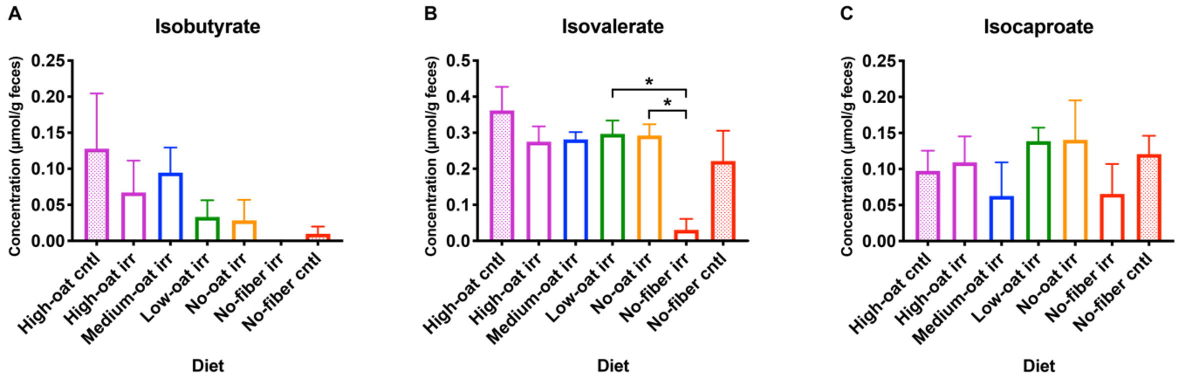

Short-chain fatty acids (SCFAs), such as isobutyrate (**A**), isovalerate (**B**), and isocaproate (**C**) were measured using gas-liquid chromatography; n = 6 mice/group, except that n = 5 mice/group were used for the High-oat cntl group. $*p \leq 0.05$.

Supplementary Figure 4. LC-MS chromatogram of mouse colonic mucin O-glycans.

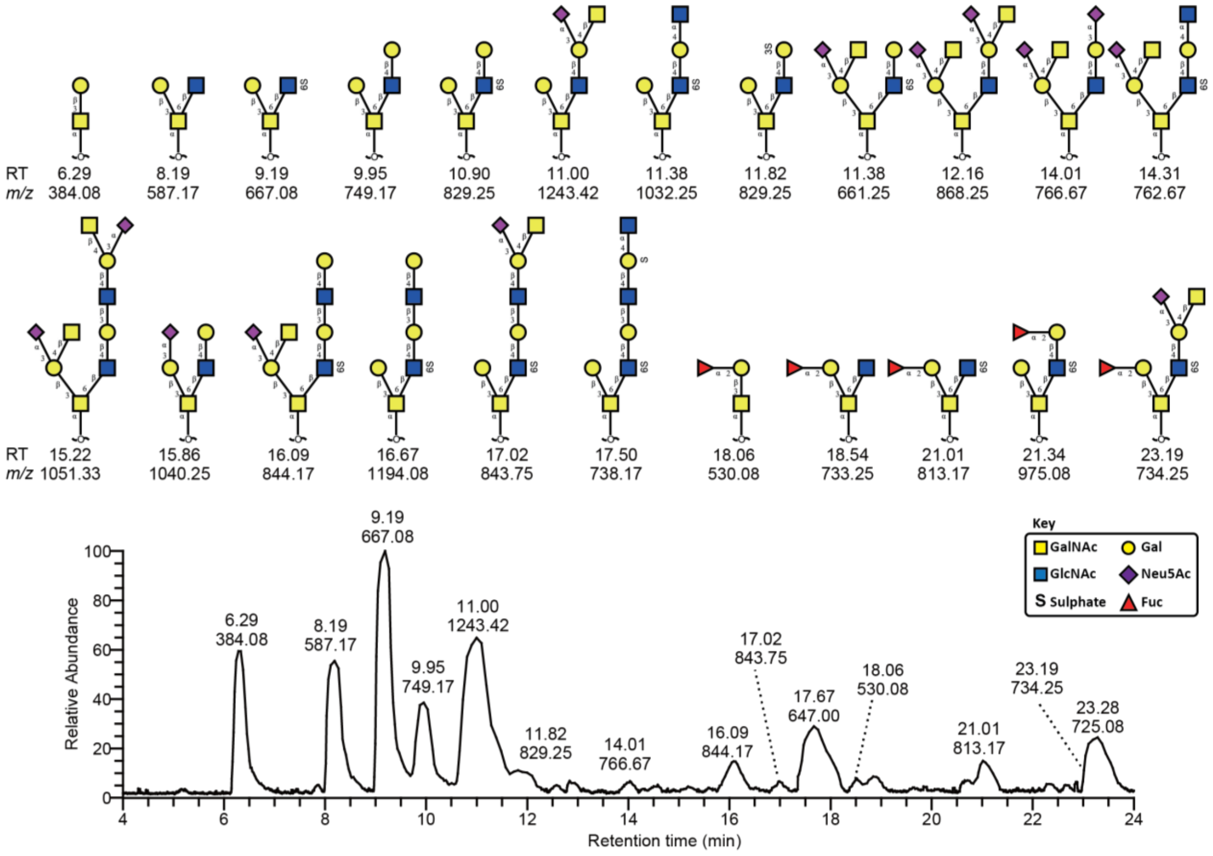

O-glycans (n = 23), including sialylated, fucosylated, Sda-containing, sulfated, and/or neutral structures, were selected for comparison between the samples. RT, retention time (in minutes); m/z, mass to charge ratio.

Supplementary Figure 5. Mouse Muc2 desialylation over time in the irradiated groups of mice fed the different diets.

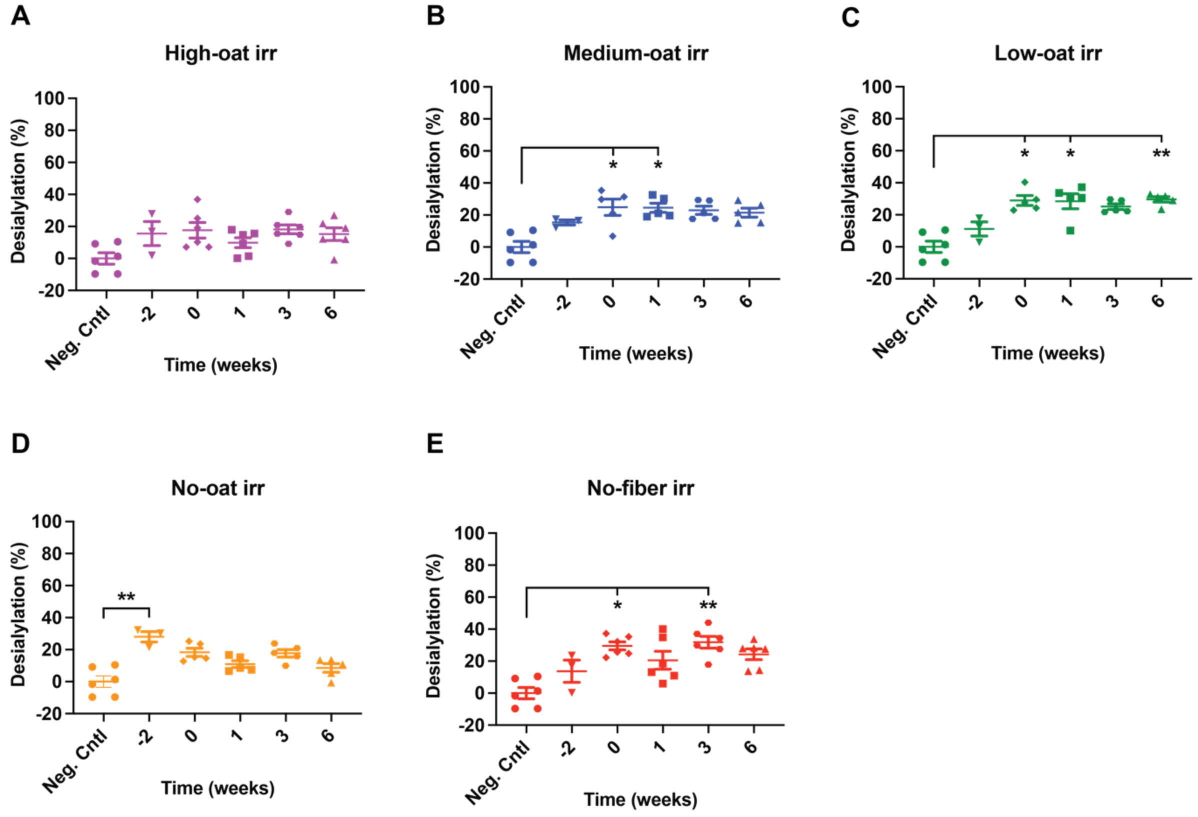

The percentages of sialic acid residues removed from the Muc2 O-glycans (desialylation) were quantified at different time-points for the irradiated groups using LC-ESI-MS/MS. The percentage of desialylation was calculated by quantifying the degradation of ten sialic acid containing structures (Neu5Ac) (structures shown in Figure S4). Desialylation was analyzed in the irradiated groups; High-oat irr (**A**), Medium-oat irr (**B**), Low-oat irr (**C**), No-oat irr (**D**), and No-fiber irr (**E**), n = 5 mice/group, except that n = 6 mice/group were used for the High-oat cntl and No-fiber cntl groups. *$p \leq 0.05$, **$p \leq 0.01$.

Supplementary Figure 6. Body weights compared between groups at different time points.

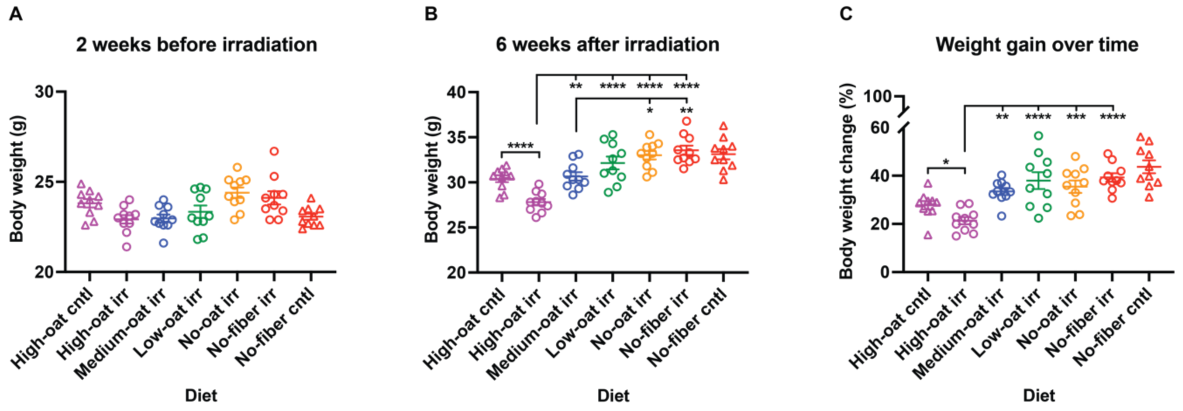

Body weights of mice from different groups measured at 2 weeks before irradiation (**A**) and at 6 weeks after irradiation (**B**). The percentage of body weight gained over time, i.e., from 2 weeks before irradiation to 6 weeks after irradiation, was calculated for each animal (**C**), n = 10 mice/group; *$p \leq 0.05$, **$p \leq 0.01$, ***$p \leq 0.001$, ****$p \leq 0.0001$.

Supplementary Table 1. Summary of alpha and beta diversity metrics by week and diet type

Week -2

Diversity measure	Diversity measure type	Comparison Group 1	vs Comparison Group 2	p-value	q-value
Bray-Curtis	Beta	All	All	0,0590	N/A
		High-oat cntl	High-oat irr	0,9780	0,9780
			Low-oat irr	0,4600	0,6038
			Medium-oat irr	0,0810	0,2430
			No-fiber cntl	0,0070	0,0368
			No-fiber irr	0,2340	0,4603
			No-oat irr	0,4970	0,6139
		High-oat irr	Low-oat irr	0,7590	0,8389
			Medium-oat irr	0,2630	0,4603
			No-fiber cntl	0,0520	0,1820
			No-fiber irr	0,3050	0,4662
			No-oat irr	0,8790	0,9230
		Low-oat irr	Medium-oat irr	0,1880	0,4387
			No-fiber cntl	0,0030	0,0315
			No-fiber irr	0,3270	0,4662
			No-oat irr	0,5550	0,6475
		Medium-oat irr	No-fiber cntl	0,0030	0,0315
			No-fiber irr	0,3330	0,4662
			No-oat irr	0,1000	0,2625
		No-fiber cntl	No-fiber irr	0,0180	0,0756
			No-oat irr	0,0050	0,0350
		No-fiber irr	No-oat irr	0,2490	0,4603
Shannon Index	Alpha	All	All	0,0003	N/A
		High-oat cntl	High-oat irr	0,5218	0,5768
			Low-oat irr	0,0782	0,1173
			Medium-oat irr	0,1093	0,1530
			No-fiber cntl	0,0104	0,0312
			No-fiber irr	0,7488	0,7488
			No-oat irr	0,1495	0,1963
		High-oat irr	Low-oat irr	0,0163	0,0428
			Medium-oat irr	0,0104	0,0312
			No-fiber cntl	0,0250	0,0583
			No-fiber irr	0,3367	0,4159
			No-oat irr	0,4233	0,4939
		Low-oat irr	Medium-oat irr	0,7488	0,7488
			No-fiber cntl	0,0039	0,0276
			No-fiber irr	0,0374	0,0713

			No-oat irr	0,0065	0,0312
		Medium-oat irr	No-fiber cntl	0,0039	0,0276
			No-fiber irr	0,0374	0,0713
			No-oat irr	0,0104	0,0312
		No-fiber cntl	No-fiber irr	0,0039	0,0276
			No-oat irr	0,0782	0,1173
		No-fiber irr	No-oat irr	0,0547	0,0957
**Simpson Index**	**Alpha**	All	All	0,0003	N/A
		High-oat cntl	High-oat irr	0,7488	0,7862
			Low-oat irr	0,0547	0,0820
			Medium-oat irr	0,0374	0,0713
			No-fiber cntl	0,0163	0,0381
			No-fiber irr	0,3367	0,4159
			No-oat irr	0,4233	0,4939
		High-oat irr	Low-oat irr	0,0163	0,0381
			Medium-oat irr	0,0163	0,0381
			No-fiber cntl	0,0547	0,0820
			No-fiber irr	0,1495	0,1963
			No-oat irr	0,8728	0,8728
		Low-oat irr	Medium-oat irr	0,7488	0,7862
			No-fiber cntl	0,0039	0,0272
			No-fiber irr	0,1495	0,1963
			No-oat irr	0,0065	0,0272
		Medium-oat irr	No-fiber cntl	0,0039	0,0272
			No-fiber irr	0,0547	0,0820
			No-oat irr	0,0065	0,0272
		No-fiber cntl	No-fiber irr	0,0039	0,0272
			No-oat irr	0,0163	0,0381
		No-fiber irr	No-oat irr	0,0250	0,0524

Week 0

Diversity measure	Diversity measure type	Comparison Group 1	vs Comparison Group 2	p-value	q-value
Bray-Curtis	Beta	All	All	0,0010	N/A
		High-oat cntl	High-oat irr	0,0890	0,1017
			Low-oat irr	0,0040	0,0060
			Medium-oat irr	0,0540	0,0667
			No-fiber cntl	0,0020	0,0060
			No-fiber irr	0,0030	0,0060
			No-oat irr	0,0060	0,0079
		High-oat irr	Low-oat irr	0,1230	0,1292
			Medium-oat irr	0,4480	0,4480
			No-fiber cntl	0,0030	0,0060
			No-fiber irr	0,0040	0,0060
			No-oat irr	0,0010	0,0060
		Low-oat irr	Medium-oat irr	0,0920	0,1017
			No-fiber cntl	0,0050	0,0070
			No-fiber irr	0,0020	0,0060
			No-oat irr	0,0030	0,0060
		Medium-oat irr	No-fiber cntl	0,0020	0,0060
			No-fiber irr	0,0010	0,0060
			No-oat irr	0,0020	0,0060
		No-fiber cntl	No-fiber irr	0,0040	0,0060
			No-oat irr	0,0020	0,0060
		No-fiber irr	No-oat irr	0,0040	0,0060
Shannon Index	Alpha	All	All	0,0000	N/A
		High-oat cntl	High-oat irr	0,2623	0,3061
			Low-oat irr	0,0039	0,0059
			Medium-oat irr	0,0782	0,1026
			No-fiber cntl	0,0039	0,0059
			No-fiber irr	0,0039	0,0059
			No-oat irr	0,0039	0,0059
		High-oat irr	Low-oat irr	0,0039	0,0059
			Medium-oat irr	0,0163	0,0228
			No-fiber cntl	0,0039	0,0059
			No-fiber irr	0,0039	0,0059
			No-oat irr	0,0039	0,0059
		Low-oat irr	Medium-oat irr	0,4233	0,4233
			No-fiber cntl	0,0039	0,0059
			No-fiber irr	0,0039	0,0059

			No-oat irr	0,0039	0,0059
		Medium-oat irr	No-fiber cntl	0,0039	0,0059
			No-fiber irr	0,0039	0,0059
			No-oat irr	0,0039	0,0059
		No-fiber cntl	No-fiber irr	0,1495	0,1847
			No-oat irr	0,4233	0,4233
		No-fiber irr	No-oat irr	0,4233	0,4233
**Simpson Index**	**Alpha**	**All**	**All**	**0,0000**	**N/A**
		High-oat cntl	High-oat irr	0,7488	0,7488
			Low-oat irr	0,0065	0,0105
			Medium-oat irr	0,0782	0,0966
			No-fiber cntl	0,0039	0,0069
			No-fiber irr	0,0039	0,0069
			No-oat irr	0,0039	0,0069
		High-oat irr	Low-oat irr	0,0104	0,0156
			Medium-oat irr	0,0547	0,0717
			No-fiber cntl	0,0039	0,0069
			No-fiber irr	0,0039	0,0069
			No-oat irr	0,0039	0,0069
		Low-oat irr	Medium-oat irr	0,3367	0,3721
			No-fiber cntl	0,0039	0,0069
			No-fiber irr	0,0039	0,0069
			No-oat irr	0,0039	0,0069
		Medium-oat irr	No-fiber cntl	0,0039	0,0069
			No-fiber irr	0,0039	0,0069
			No-oat irr	0,0039	0,0069
		No-fiber cntl	No-fiber irr	0,1093	0,1275
			No-oat irr	0,4233	0,4445
		No-fiber irr	No-oat irr	0,0374	0,0523

Week 1

Diversity measure	Diversity measure type	Comparison Group 1	vs Comparison Group 2	p-value	q-value
Bray-Curtis	Beta	All	All	0,0010	N/A
		High-oat cntl	High-oat irr	0,1880	0,1974
			Low-oat irr	0,0030	0,0063
			Medium-oat irr	0,1450	0,1603
			No-fiber cntl	0,0020	0,0060
			No-fiber irr	0,0020	0,0060
			No-oat irr	0,0020	0,0060
		High-oat irr	Low-oat irr	0,0070	0,0086
			Medium-oat irr	0,2320	0,2320
			No-fiber cntl	0,0020	0,0060
			No-fiber irr	0,0050	0,0066
			No-oat irr	0,0020	0,0060
		Low-oat irr	Medium-oat irr	0,0280	0,0327
			No-fiber cntl	0,0040	0,0065
			No-fiber irr	0,0040	0,0065
			No-oat irr	0,0040	0,0065
		Medium-oat irr	No-fiber cntl	0,0020	0,0060
			No-fiber irr	0,0050	0,0066
			No-oat irr	0,0020	0,0060
		No-fiber cntl	No-fiber irr	0,0050	0,0066
			No-oat irr	0,0030	0,0063
		No-fiber irr	No-oat irr	0,0030	0,0063
Shannon Index	Alpha	All	All	0,0001	N/A
		High-oat cntl	High-oat irr	0,8728	0,9164
			Low-oat irr	0,2623	0,3241
			Medium-oat irr	0,4233	0,4939
			No-fiber cntl	0,0163	0,0311
			No-fiber irr	0,0039	0,0104
			No-oat irr	0,0039	0,0104
		High-oat irr	Low-oat irr	0,1093	0,1530
			Medium-oat irr	0,2002	0,2627
			No-fiber cntl	0,0163	0,0311
			No-fiber irr	0,0039	0,0104
			No-oat irr	0,0039	0,0104
		Low-oat irr	Medium-oat irr	1,0000	1,0000
			No-fiber cntl	0,0547	0,0957
			No-fiber irr	0,0039	0,0104

			No-oat irr	0,0039	0,0104
		Medium-oat irr	No-fiber cntl	0,1093	0,1530
			No-fiber irr	0,0039	0,0104
			No-oat irr	0,0039	0,0104
		No-fiber cntl	No-fiber irr	0,0104	0,0243
			No-oat irr	0,0782	0,1263
		No-fiber irr	No-oat irr	0,5218	0,5768
Simpson Index	Alpha	All	All	0,0000	N/A
		High-oat cntl	High-oat irr	0,6310	0,6974
			Low-oat irr	0,0782	0,1026
			Medium-oat irr	0,1495	0,1847
			No-fiber cntl	0,0039	0,0083
			No-fiber irr	0,0039	0,0083
			No-oat irr	0,0039	0,0083
		High-oat irr	Low-oat irr	0,0782	0,1026
			Medium-oat irr	0,2623	0,3061
			No-fiber cntl	0,0039	0,0083
			No-fiber irr	0,0039	0,0083
			No-oat irr	0,0039	0,0083
		Low-oat irr	Medium-oat irr	0,7488	0,7488
			No-fiber cntl	0,0104	0,0199
			No-fiber irr	0,0039	0,0083
			No-oat irr	0,0039	0,0083
		Medium-oat irr	No-fiber cntl	0,0163	0,0285
			No-fiber irr	0,0039	0,0083
			No-oat irr	0,0039	0,0083
		No-fiber cntl	No-fiber irr	0,0250	0,0375
			No-oat irr	0,0250	0,0375
		No-fiber irr	No-oat irr	0,7488	0,7488

Week 3

Diversity measure	Diversity measure type	Comparison Group 1	vs Comparison Group 2	p-value	q-value
Bray-Curtis	Beta	All	All	0,0010	N/A
		High-oat cntl	High-oat irr	0,1050	0,1103
			Low-oat irr	0,0020	0,0053
			Medium-oat irr	0,0080	0,0093
			No-fiber cntl	0,0030	0,0053
			No-fiber irr	0,0030	0,0053
			No-oat irr	0,0020	0,0053
		High-oat irr	Low-oat irr	0,0010	0,0053
			Medium-oat irr	0,0270	0,0298
			No-fiber cntl	0,0050	0,0066
			No-fiber irr	0,0020	0,0053
			No-oat irr	0,0050	0,0066
		Low-oat irr	Medium-oat irr	0,0050	0,0066
			No-fiber cntl	0,0030	0,0053
			No-fiber irr	0,0070	0,0086
			No-oat irr	0,0020	0,0053
		Medium-oat irr	No-fiber cntl	0,0020	0,0053
			No-fiber irr	0,0010	0,0053
			No-oat irr	0,0030	0,0053
		No-fiber cntl	No-fiber irr	0,2230	0,2230
			No-oat irr	0,0040	0,0065
		No-fiber irr	No-oat irr	0,0030	0,0053
Shannon Index	Alpha	All	All	0,0000	N/A
		High-oat cntl	High-oat irr	0,0374	0,0523
			Low-oat irr	0,0039	0,0118
			Medium-oat irr	0,0547	0,0675
			No-fiber cntl	0,0104	0,0182
			No-fiber irr	0,0039	0,0118
			No-oat irr	0,0039	0,0118
		High-oat irr	Low-oat irr	0,0039	0,0118
			Medium-oat irr	0,0065	0,0136
			No-fiber cntl	0,0039	0,0118
			No-fiber irr	0,0039	0,0118
			No-oat irr	0,0039	0,0118
		Low-oat irr	Medium-oat irr	0,0374	0,0523
			No-fiber cntl	0,3367	0,3535
			No-fiber irr	0,0065	0,0136

			No-oat irr	0,0547	0,0675
		Medium-oat irr	No-fiber cntl	0,0374	0,0523
			No-fiber irr	0,0065	0,0136
			No-oat irr	0,0104	0,0182
		No-fiber cntl	No-fiber irr	0,1495	0,1653
			No-oat irr	0,1495	0,1653
		No-fiber irr	No-oat irr	0,7488	0,7488
Simpson Index	Alpha	All	All	0,0000	N/A
		High-oat cntl	High-oat irr	0,6310	0,6310
			Low-oat irr	0,0039	0,0064
			Medium-oat irr	0,0163	0,0201
			No-fiber cntl	0,0039	0,0064
			No-fiber irr	0,0039	0,0064
			No-oat irr	0,0039	0,0064
		High-oat irr	Low-oat irr	0,0039	0,0064
			Medium-oat irr	0,0065	0,0091
			No-fiber cntl	0,0039	0,0064
			No-fiber irr	0,0039	0,0064
			No-oat irr	0,0039	0,0064
		Low-oat irr	Medium-oat irr	0,0104	0,0137
			No-fiber cntl	0,0065	0,0091
			No-fiber irr	0,0039	0,0064
			No-oat irr	0,0039	0,0064
		Medium-oat irr	No-fiber cntl	0,0039	0,0064
			No-fiber irr	0,0039	0,0064
			No-oat irr	0,0039	0,0064
		No-fiber cntl	No-fiber irr	0,1495	0,1653
			No-oat irr	0,0782	0,0912
		No-fiber irr	No-oat irr	0,6310	0,6310

Week 6

Diversity measure	Diversity measure type	Comparison Group 1	vs Comparison Group 2	p-value	q-value
Bray-Curtis	Beta	All	All	0,0010	N/A
		High-oat cntl	High-oat irr	0,2820	0,2820
			Low-oat irr	0,0110	0,0136
			Medium-oat irr	0,0340	0,0376
			No-fiber cntl	0,0020	0,0063
			No-fiber irr	0,0060	0,0090
			No-oat irr	0,0020	0,0063
		High-oat irr	Low-oat irr	0,0100	0,0131
			Medium-oat irr	0,0030	0,0063
			No-fiber cntl	0,0040	0,0070
			No-fiber irr	0,0030	0,0063
			No-oat irr	0,0040	0,0070
		Low-oat irr	Medium-oat irr	0,0140	0,0163
			No-fiber cntl	0,0050	0,0081
			No-fiber irr	0,0080	0,0112
			No-oat irr	0,0030	0,0063
		Medium-oat irr	No-fiber cntl	0,0020	0,0063
			No-fiber irr	0,0010	0,0063
			No-oat irr	0,0020	0,0063
		No-fiber cntl	No-fiber irr	0,1460	0,1533
			No-oat irr	0,0020	0,0063
		No-fiber irr	No-oat irr	0,0030	0,0063
Shannon Index	Alpha	All	All	0,0001	N/A
		High-oat cntl	High-oat irr	0,2623	0,3443
			Low-oat irr	0,0104	0,0199
			Medium-oat irr	0,0065	0,0136
			No-fiber cntl	0,0039	0,0136
			No-fiber irr	0,0039	0,0136
			No-oat irr	0,0039	0,0136
		High-oat irr	Low-oat irr	0,0374	0,0604
			Medium-oat irr	0,0065	0,0136
			No-fiber cntl	0,0065	0,0136
			No-fiber irr	0,0039	0,0136
			No-oat irr	0,0039	0,0136
		Low-oat irr	Medium-oat irr	0,0782	0,1173
			No-fiber cntl	0,0163	0,0285
			No-fiber irr	0,0065	0,0136

			No-oat irr	0,0065	0,0136
		Medium-oat irr	No-fiber cntl	1,0000	1,0000
			No-fiber irr	0,2623	0,3443
			No-oat irr	0,7488	0,7862
		No-fiber cntl	No-fiber irr	0,4233	0,5229
			No-oat irr	0,7488	0,7862
		No-fiber irr	No-oat irr	0,5218	0,6088
Simpson Index	Alpha	All	All	0,0000	N/A
		High-oat cntl	High-oat irr	0,2623	0,2754
			Low-oat irr	0,0039	0,0069
			Medium-oat irr	0,0039	0,0069
			No-fiber cntl	0,0039	0,0069
			No-fiber irr	0,0039	0,0069
			No-oat irr	0,0039	0,0069
		High-oat irr	Low-oat irr	0,0163	0,0263
			Medium-oat irr	0,0039	0,0069
			No-fiber cntl	0,0039	0,0069
			No-fiber irr	0,0039	0,0069
			No-oat irr	0,0039	0,0069
		Low-oat irr	Medium-oat irr	0,0782	0,1026
			No-fiber cntl	0,0039	0,0069
			No-fiber irr	0,0039	0,0069
			No-oat irr	0,0039	0,0069
		Medium-oat irr	No-fiber cntl	0,2002	0,2213
			No-fiber irr	0,0250	0,0350
			No-oat irr	0,0250	0,0350
		No-fiber cntl	No-fiber irr	0,1093	0,1350
			No-oat irr	0,1495	0,1745
		No-fiber irr	No-oat irr	1,0000	1,0000

Supplementary Table 2. Composition of the basal mixture

**Basal mixture** *(g/100g in dwb*)*	
Casein	13.3
DL-Methionine	0.2
Corn starch	25.0
Maltodextrin	8.7
Sucrose	10.6
Olive oil	4.7
Vitamin mixture	1.0
Choline bitartrate	0.2
TBHQ[#]	0.001
Mineral mixture	1.3
Calcium phosphate	1.1
Calcium carbonate	0.4
**Total weight (g)**	**66.5**

*Dry-weight basis. [#]Tertiary butylhydroquinone

Supplementary Table 3. Outer PCR (PCR2) primer design

Forward Primer ID	Forward Primer Sequence	Reverse Primer ID	Reverse Primer Sequence
FP1	AATGATACGGCGACCACCGAGATCTACAC GGAGTGGGTCGTCGGCAGCGTC	RP1	CAAGCAGAAGACGGCATACGAGAT TGGTGGTGGTCTCGTGGGCTCGG
FP2	AATGATACGGCGACCACCGAGATCTACAC AGCGTTTATCGTCGGCAGCGTC	RP2	CAAGCAGAAGACGGCATACGAGAT CCTAAGTAGTCTCGTGGGCTCGG
FP3	AATGATACGGCGACCACCGAGATCTACAC TCTACTAATCGTCGGCAGCGTC	RP3	CAAGCAGAAGACGGCATACGAGAT AATAATGAGTCTCGTGGGCTCGG
FP4	AATGATACGGCGACCACCGAGATCTACAC TAGTCAGCTCGTCGGCAGCGTC	RP4	CAAGCAGAAGACGGCATACGAGAT TCGTTTATGTCTCGTGGGCTCGG
FP5	AATGATACGGCGACCACCGAGATCTACAC CAATTAAATCGTCGGCAGCGTC	RP5	CAAGCAGAAGACGGCATACGAGAT TTCCTGTTGTCTCGTGGGCTCGG
FP6	AATGATACGGCGACCACCGAGATCTACAC TATATCAATCGTCGGCAGCGTC	RP6	CAAGCAGAAGACGGCATACGAGAT TAATTTACGTCTCGTGGGCTCGG
FP7	AATGATACGGCGACCACCGAGATCTACAC GCTGAAATTCGTCGGCAGCGTC	RP7	CAAGCAGAAGACGGCATACGAGAT CATGAGATGTCTCGTGGGCTCGG
FP8	AATGATACGGCGACCACCGAGATCTACAC ATGATTAATCGTCGGCAGCGTC	RP8	CAAGCAGAAGACGGCATACGAGAT AAATCTTCGTCTCGTGGGCTCGG
FP9	AATGATACGGCGACCACCGAGATCTACAC GTAGCAATTCGTCGGCAGCGTC	RP9	CAAGCAGAAGACGGCATACGAGAT GACTACATGTCTCGTGGGCTCGG
FP10	AATGATACGGCGACCACCGAGATCTACAC AATTCATATCGTCGGCAGCGTC	RP10	CAAGCAGAAGACGGCATACGAGAT TGGACCTTGTCTCGTGGGCTCGG
FP11	AATGATACGGCGACCACCGAGATCTACAC GAGCATGCTCGTCGGCAGCGTC		
FP12	AATGATACGGCGACCACCGAGATCTACAC CAATAGCCTCGTCGGCAGCGTC		

Black – Handle, Red – Index/Barcode & Green – Adapter
