# Supplementary material for: Oat bran fiber protects against radiation-induced disruption of gut barrier dynamics and mucosal damage
Source: NPJ Biofilms Microbiomes. 2025 Jul 4;11:128. doi: 10.1038/s41522-025-00759-x (PMC12227613; doi:10.1038/s41522-025-00759-x)
Supplement: Supplementary file 3 — LC-ESI-MS_MS spectra of selected structures [file 41522_2025_759_MOESM3_ESM.pdf]

MS2 Spectra

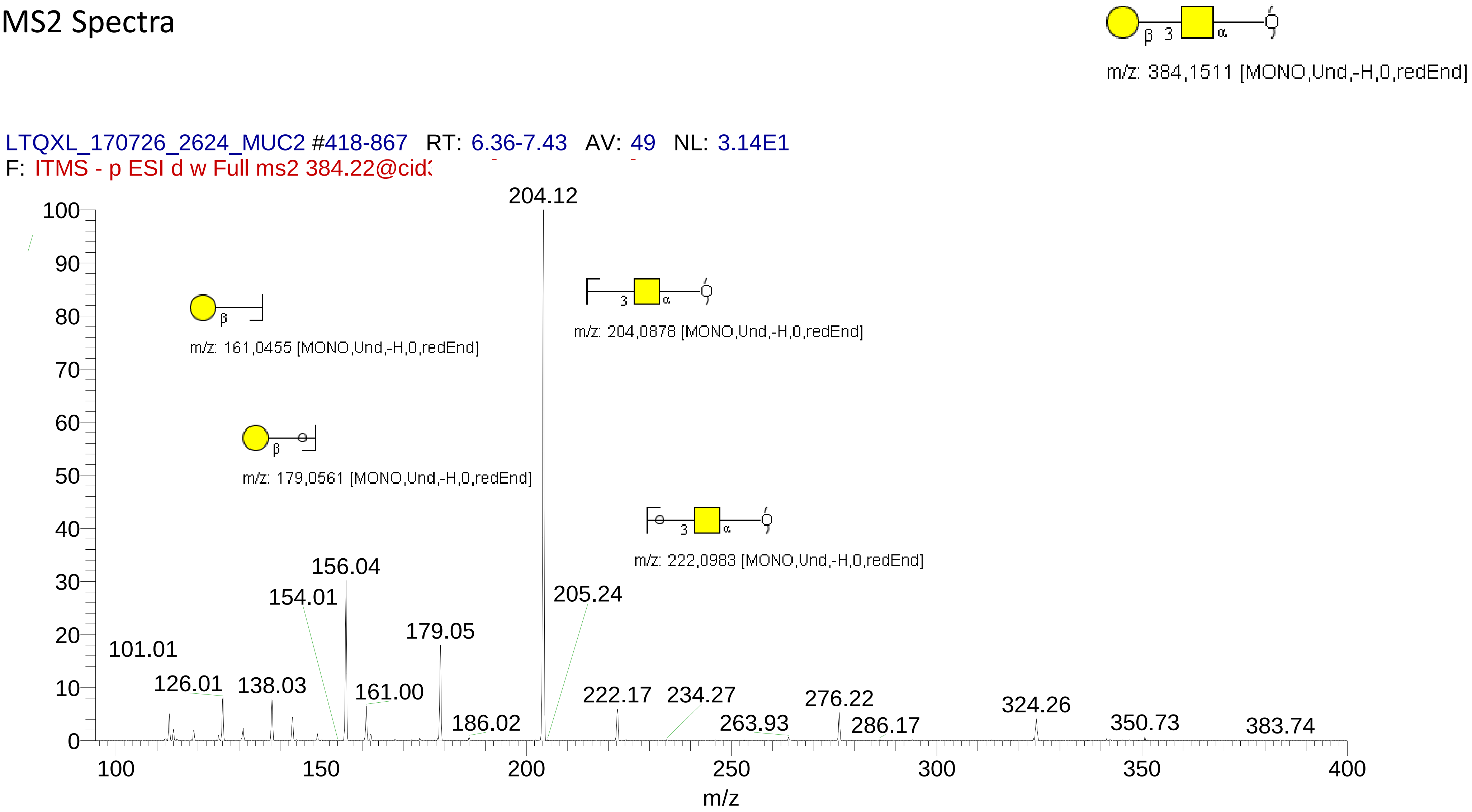

β 3 α
m/z: 384,1511 [MONO,Und,-H,0,redEnd]
LTQXL_170726_2624_MUC2 #418-867 RT: 6.36-7.43 AV: 49 NL: 3.14E1
F: ITMS - p ESI d w Full ms2 384.22@cid
204.12
β
m/z: 161,0455 [MONO,Und,-H,0,redEnd]
3 α
m/z: 204,0878 [MONO,Und,-H,0,redEnd]
β
m/z: 179,0561 [MONO,Und,-H,0,redEnd]
3 α
m/z: 222,0983 [MONO,Und,-H,0,redEnd]
156.04
154.01
205.24
179.05
101.01
126.01
138.03
161.00
186.02
222.17
234.27
263.93
276.22
286.17
324.26
350.73
383.74
100
90
80
70
60
50
40
30
20
10
0
100
150
200
250
300
350
400
m/z

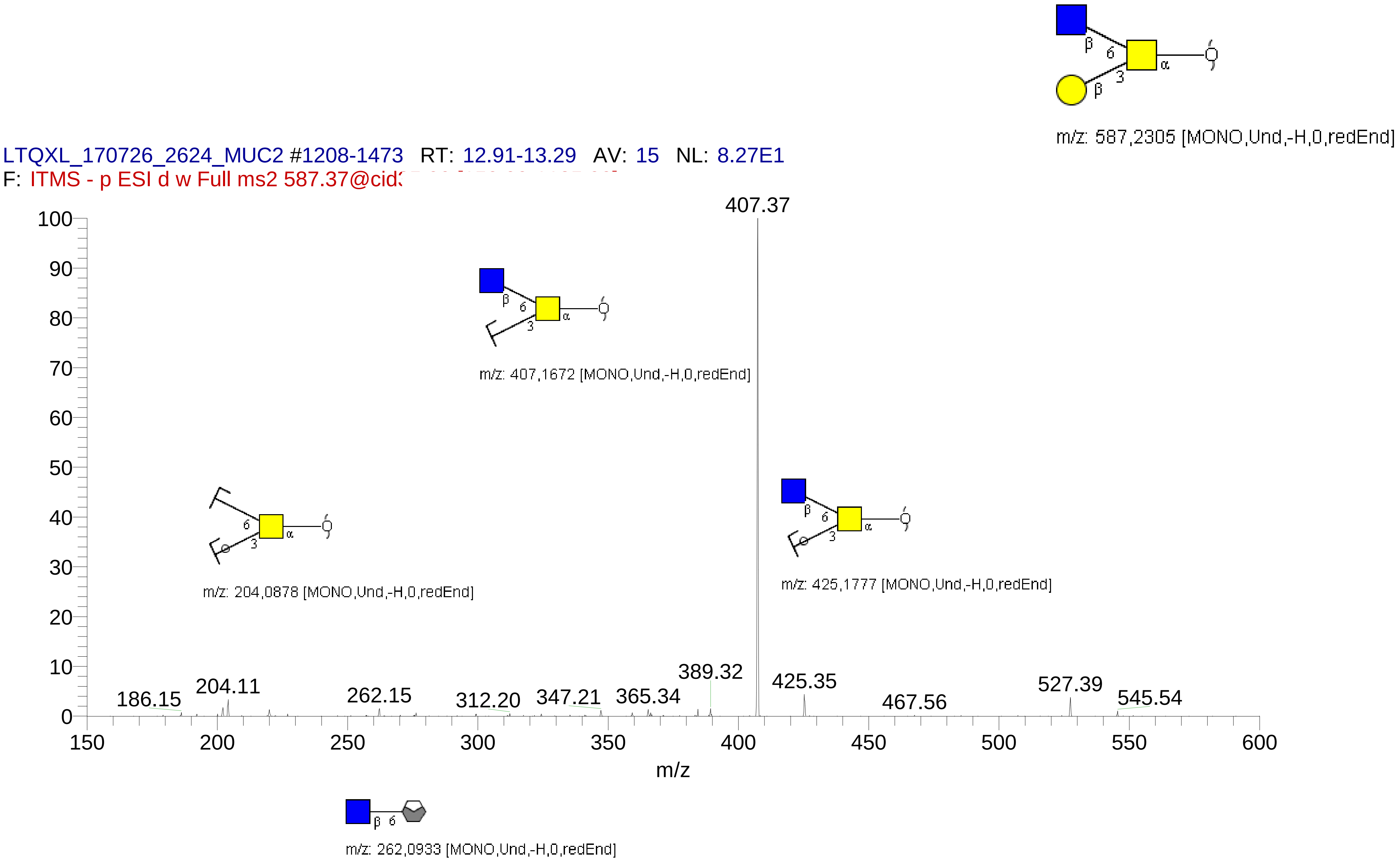
m/z: 587,2305 [MONO,Und,-H,0,redEnd]
LTQXL_170726_2624_MUC2 #1208-1473 RT: 12.91-13.29 AV: 15 NL: 8.27E1
F: ITMS - p ESI d w Full ms2 587.37@cid3
407.37
m/z: 407,1672 [MONO,Und,-H,0,redEnd]
m/z: 204,0878 [MONO,Und,-H,0,redEnd]
m/z: 425,1777 [MONO,Und,-H,0,redEnd]
186.15
204.11
262.15
312.20
347.21
365.34
389.32
425.35
467.56
527.39
545.54
m/z
m/z: 262,0933 [MONO,Und,-H,0,redEnd]

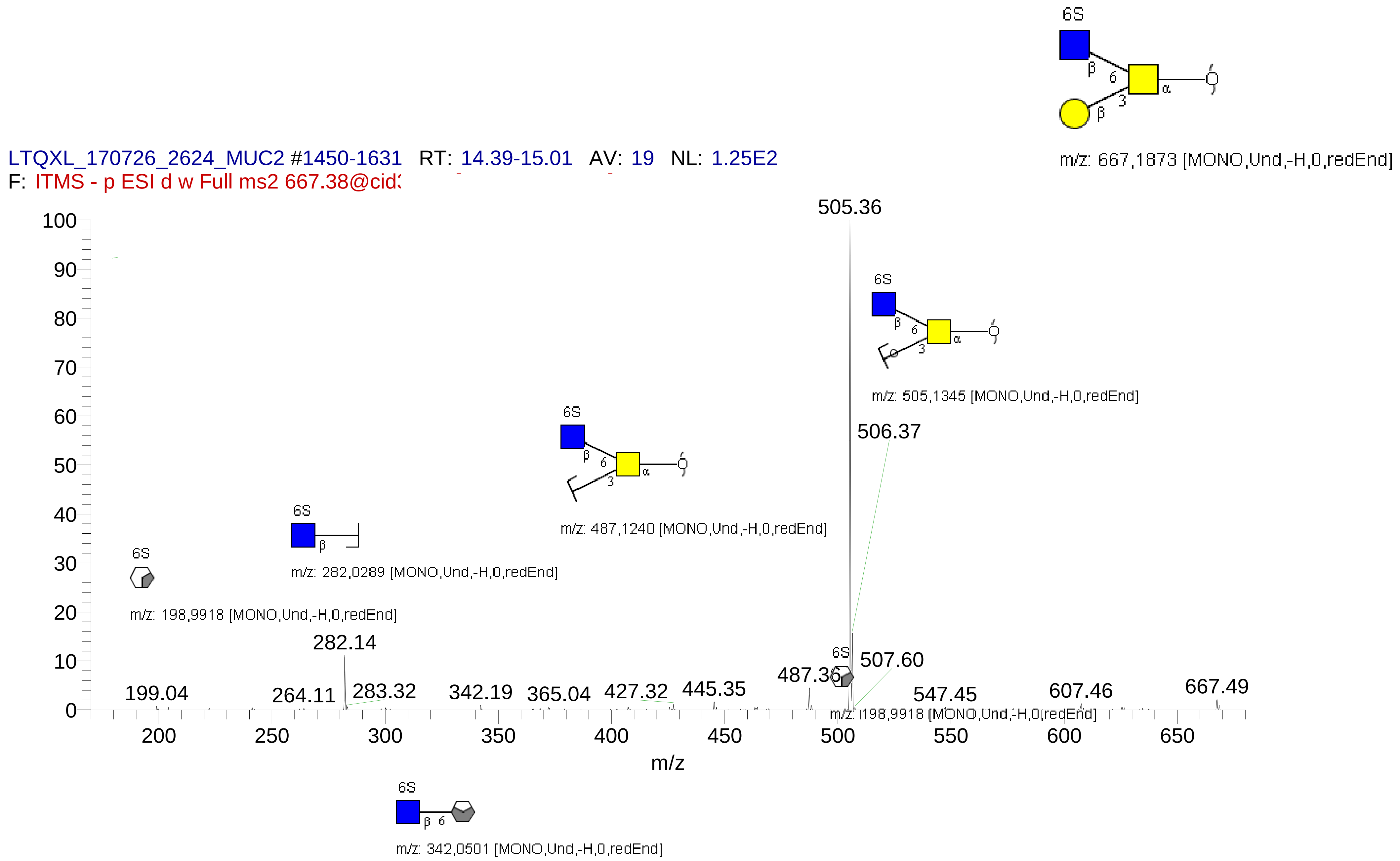

LTQXL_170726_2624_MUC2 #1450-1631 RT: 14.39-15.01 AV: 19 NL: 1.25E2
F: ITMS - p ESI d w Full ms2 667.38@cid
6S
m/z: 667,1873 [MONO,Und,-H,0,redEnd]
505.36
m/z: 505,1345 [MONO,Und,-H,0,redEnd]
506.37
m/z: 487,1240 [MONO,Und,-H,0,redEnd]
m/z: 282,0289 [MONO,Und,-H,0,redEnd]
m/z: 198,9918 [MONO,Und,-H,0,redEnd]
282.14
199.04
264.11
283.32
342.19
365.04
427.32
445.35
487.36
507.60
547.45
607.46
667.49
m/z: 198,9918 [MONO,Und,-H,0,redEnd]
m/z
m/z: 342,0501 [MONO,Und,-H,0,redEnd]

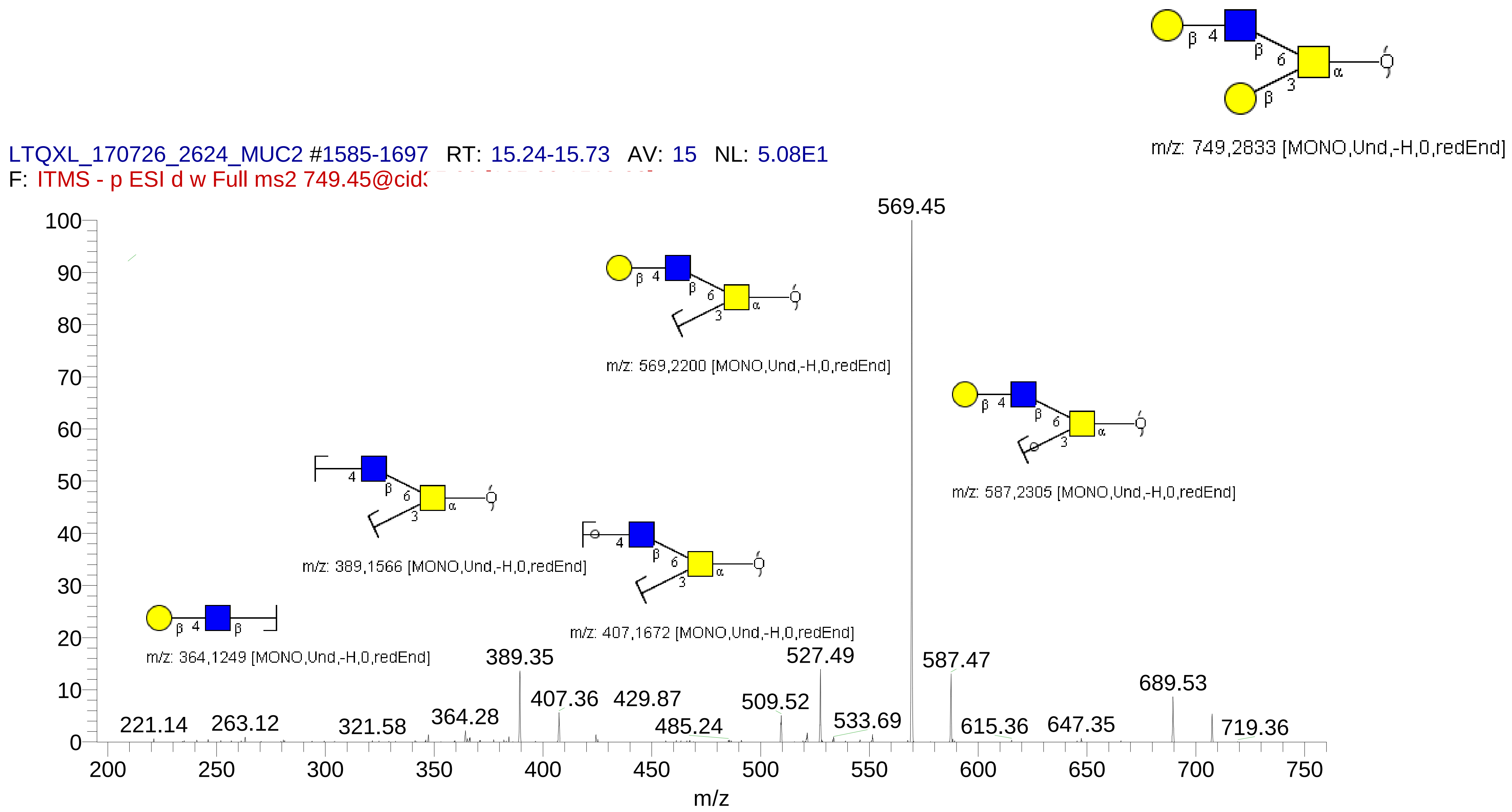

m/z: 749,2833 [MONO,Und,-H,0,redEnd]
LTQXL_170726_2624_MUC2 #1585-1697 RT: 15.24-15.73 AV: 15 NL: 5.08E1
F: ITMS - p ESI d w Full ms2 749.45@cid
569.45
m/z: 569,2200 [MONO,Und,-H,0,redEnd]
m/z: 587,2305 [MONO,Und,-H,0,redEnd]
m/z: 389,1566 [MONO,Und,-H,0,redEnd]
m/z: 407,1672 [MONO,Und,-H,0,redEnd]
m/z: 364,1249 [MONO,Und,-H,0,redEnd]
389.35
527.49
587.47
689.53
407.36
429.87
509.52
533.69
221.14
263.12
321.58
364.28
485.24
615.36
647.35
719.36
m/z

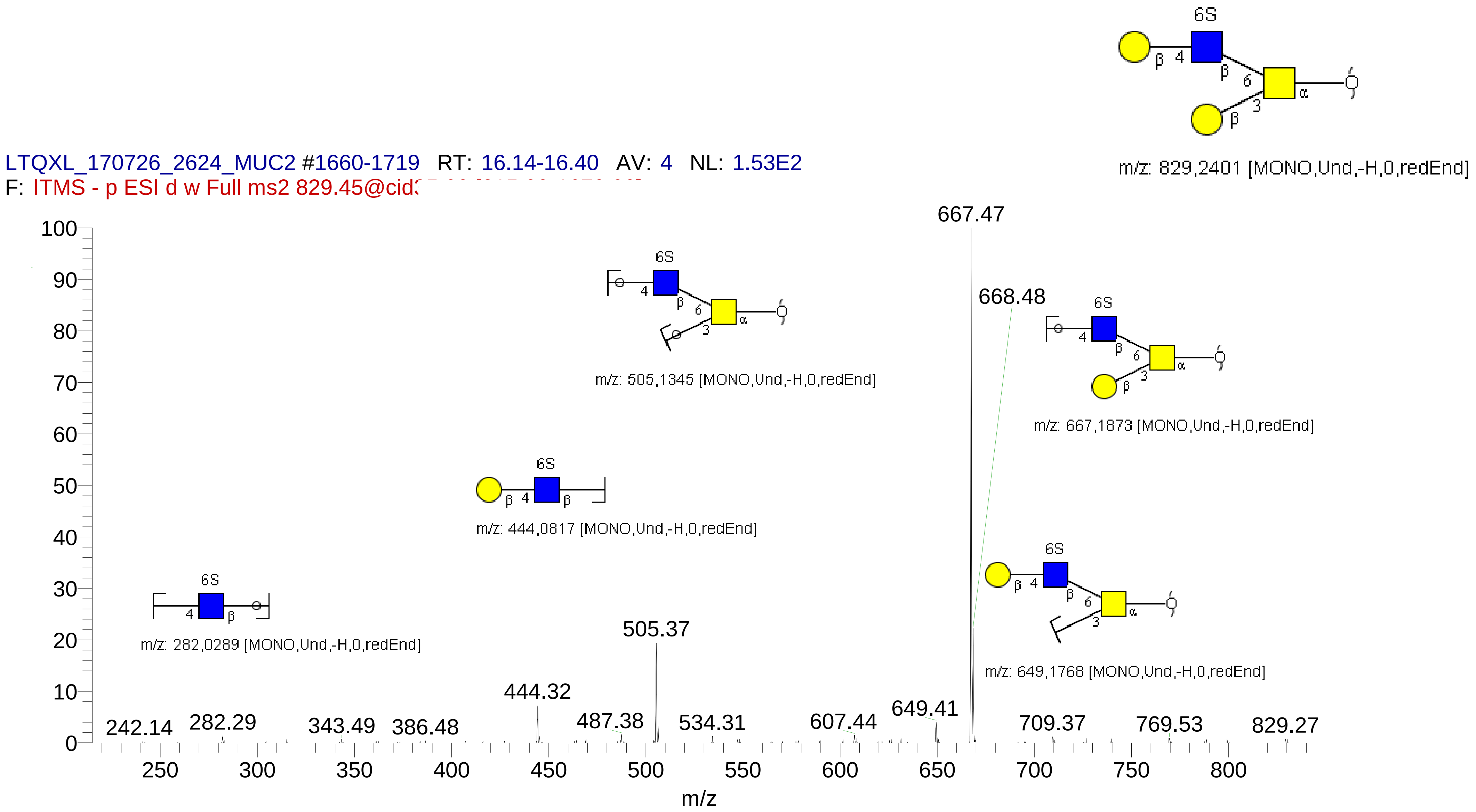

LTQXL_170726_2624_MUC2 #1660-1719 RT: 16.14-16.40 AV: 4 NL: 1.53E2
F: ITMS - p ESI d w Full ms2 829.45@cid3
6S
m/z: 829,2401 [MONO,Und,-H,0,redEnd]
667.47
668.48
m/z: 505,1345 [MONO,Und,-H,0,redEnd]
m/z: 667,1873 [MONO,Und,-H,0,redEnd]
m/z: 444,0817 [MONO,Und,-H,0,redEnd]
m/z: 649,1768 [MONO,Und,-H,0,redEnd]
m/z: 282,0289 [MONO,Und,-H,0,redEnd]
505.37
444.32
242.14
282.29
343.49
386.48
487.38
534.31
607.44
649.41
709.37
769.53
829.27
m/z

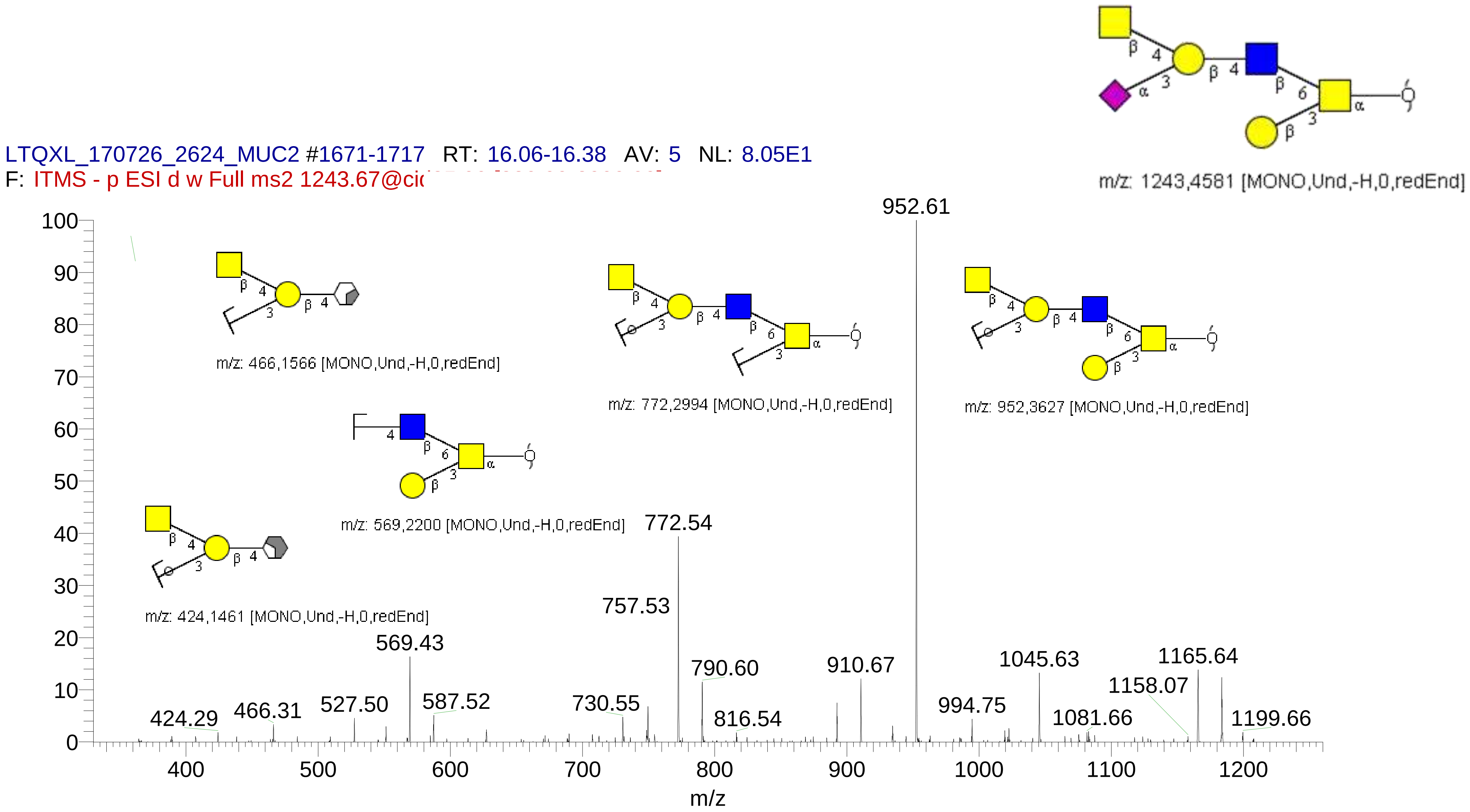

LTQXL_170726_2624_MUC2 #1671-1717 RT: 16.06-16.38 AV: 5 NL: 8.05E1
F: ITMS - p ESI d w Full ms2 1243.67@cid
m/z: 1243,4581 [MONO,Und,-H,0,redEnd]
m/z: 466,1566 [MONO,Und,-H,0,redEnd]
m/z: 772,2994 [MONO,Und,-H,0,redEnd]
m/z: 952,3627 [MONO,Und,-H,0,redEnd]
m/z: 569,2200 [MONO,Und,-H,0,redEnd]
m/z: 424,1461 [MONO,Und,-H,0,redEnd]
952.61
772.54
757.53
569.43
790.60
910.67
1045.63
1165.64
1158.07
994.75
1081.66
1199.66
816.54
730.55
587.52
527.50
466.31
424.29
m/z

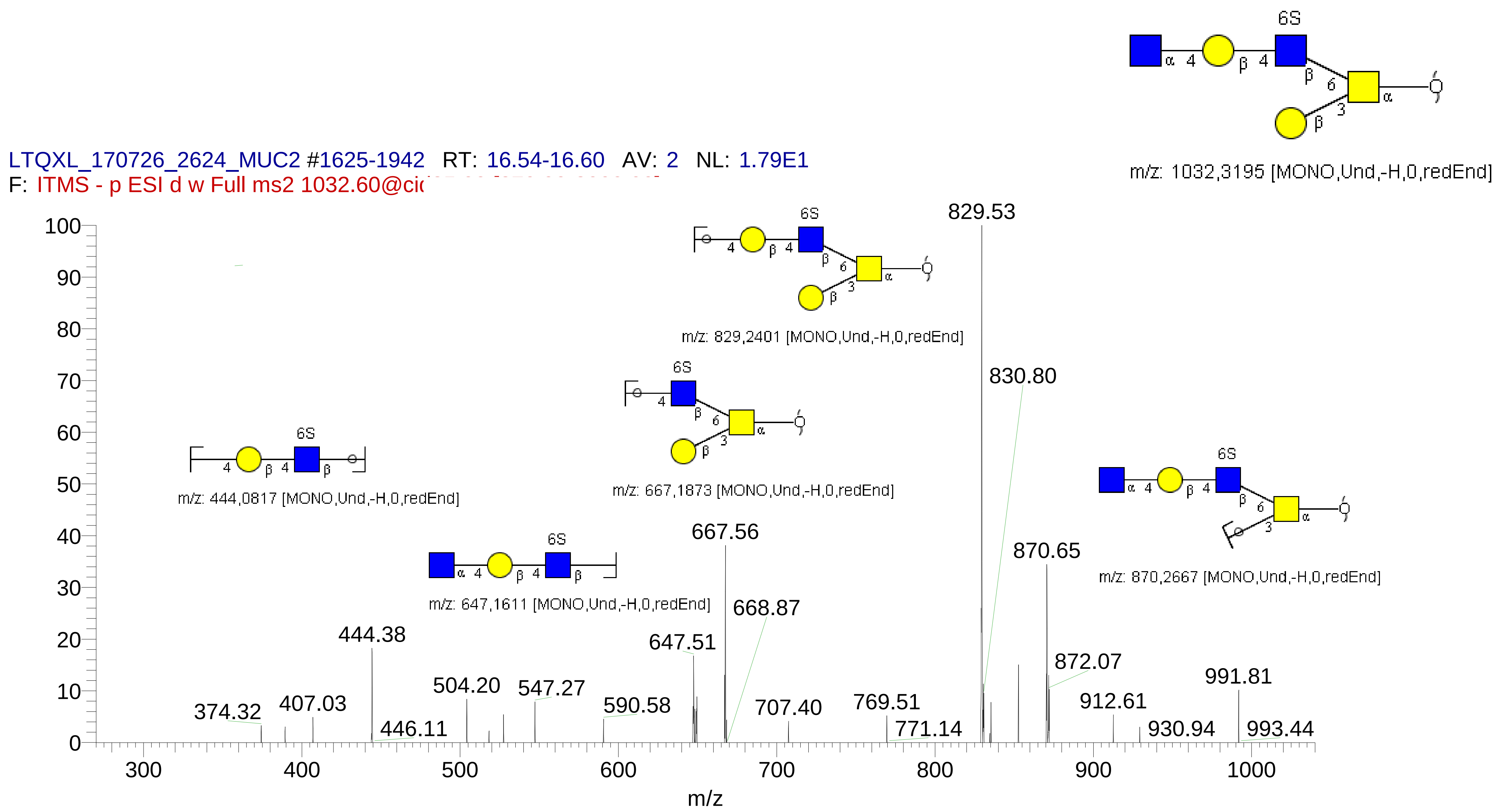
6S
m/z: 1032,3195 [MONO,Und,-H,0,redEnd]
LTQXL_170726_2624_MUC2 #1625-1942 RT: 16.54-16.60 AV: 2 NL: 1.79E1
F: ITMS - p ESI d w Full ms2 1032.60@cid
829.53
m/z: 829,2401 [MONO,Und,-H,0,redEnd]
830.80
m/z: 667,1873 [MONO,Und,-H,0,redEnd]
m/z: 444,0817 [MONO,Und,-H,0,redEnd]
m/z: 870,2667 [MONO,Und,-H,0,redEnd]
667.56
870.65
668.87
m/z: 647,1611 [MONO,Und,-H,0,redEnd]
444.38
647.51
872.07
991.81
504.20
547.27
912.61
374.32
407.03
590.58
707.40
769.51
446.11
771.14
930.94
993.44
m/z

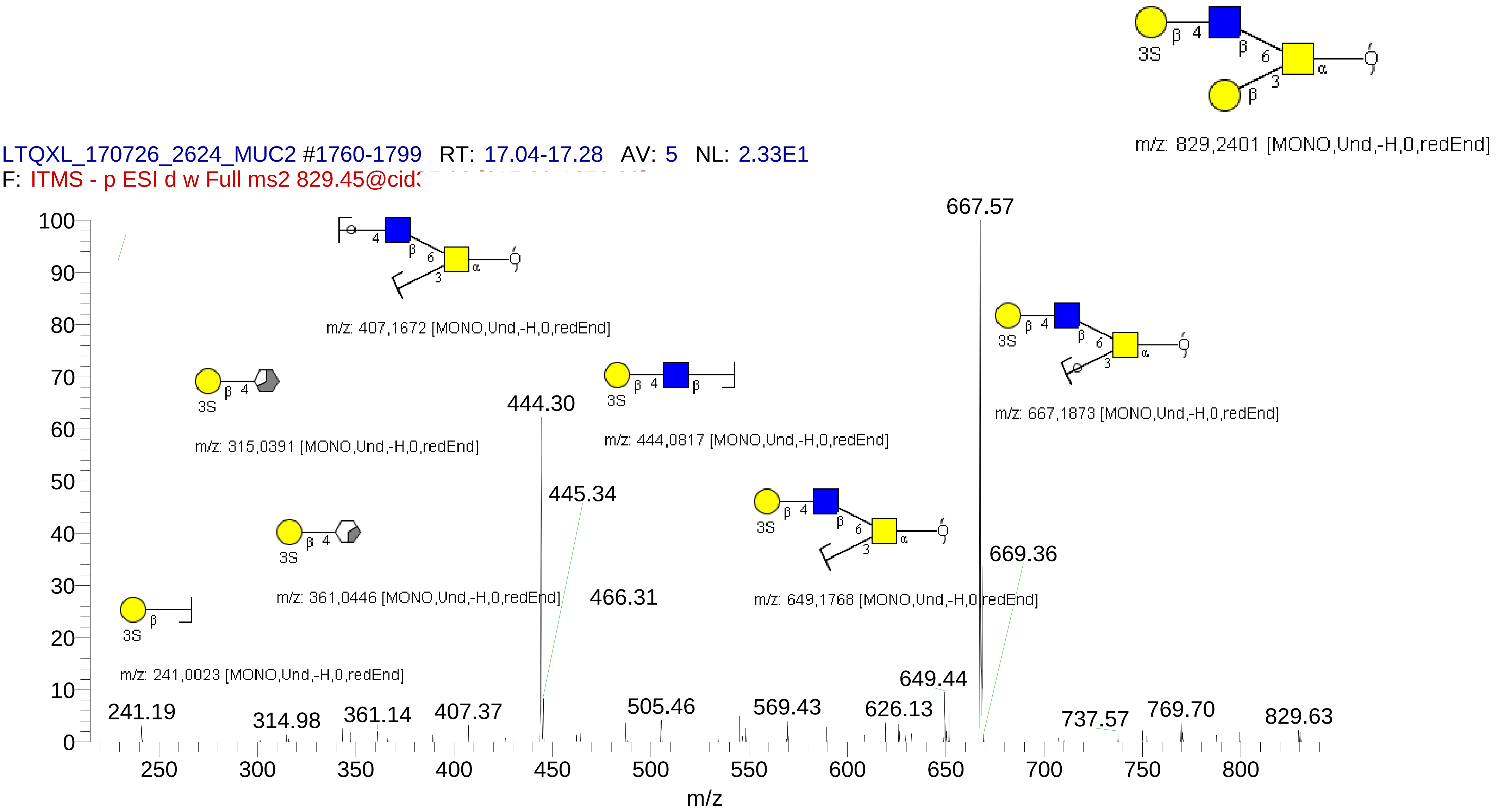

m/z: 829,2401 [MONO,Und,-H,0,redEnd]
LTQXL_170726_2624_MUC2 #1760-1799 RT: 17.04-17.28 AV: 5 NL: 2.33E1
F: ITMS - p ESI d w Full ms2 829.45@cid3
m/z: 407,1672 [MONO,Und,-H,0,redEnd]
m/z: 315,0391 [MONO,Und,-H,0,redEnd]
m/z: 444,0817 [MONO,Und,-H,0,redEnd]
m/z: 667,1873 [MONO,Und,-H,0,redEnd]
m/z: 361,0446 [MONO,Und,-H,0,redEnd]
m/z: 649,1768 [MONO,Und,-H,0,redEnd]
m/z: 241,0023 [MONO,Und,-H,0,redEnd]
3S
667.57
444.30
445.34
669.36
466.31
649.44
241.19
314.98
361.14
407.37
505.46
569.43
626.13
737.57
769.70
829.63
m/z

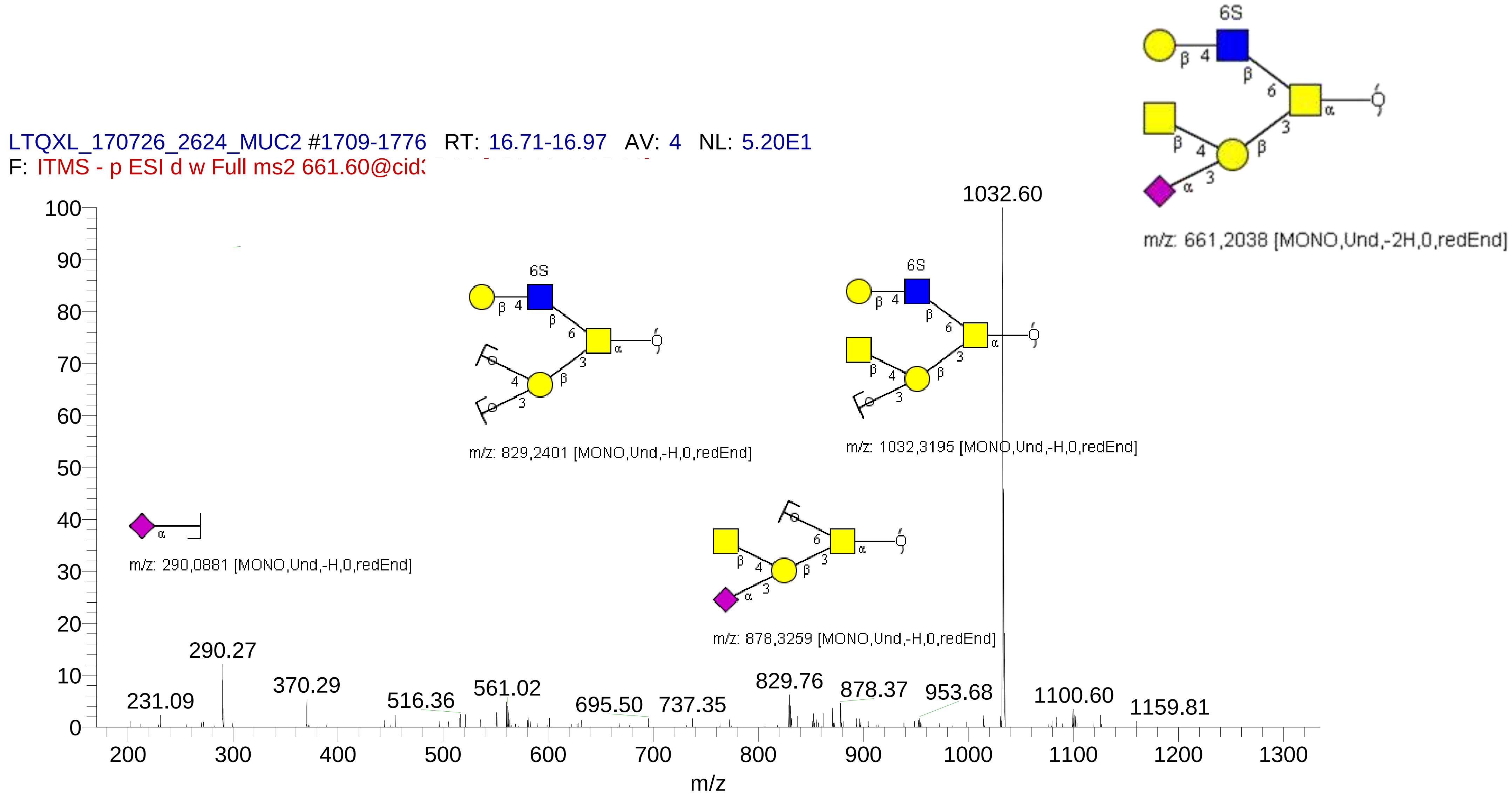

LTQXL_170726_2624_MUC2 #1709-1776 RT: 16.71-16.97 AV: 4 NL: 5.20E1
F: ITMS - p ESI d w Full ms2 661.60@cid3
m/z: 661,2038 [MONO,Und,-2H,0,redEnd]
m/z: 829,2401 [MONO,Und,-H,0,redEnd]
m/z: 1032,3195 [MONO,Und,-H,0,redEnd]
m/z: 290,0881 [MONO,Und,-H,0,redEnd]
m/z: 878,3259 [MONO,Und,-H,0,redEnd]
1032.60
290.27
231.09
370.29
516.36
561.02
695.50
737.35
829.76
878.37
953.68
1100.60
1159.81
m/z

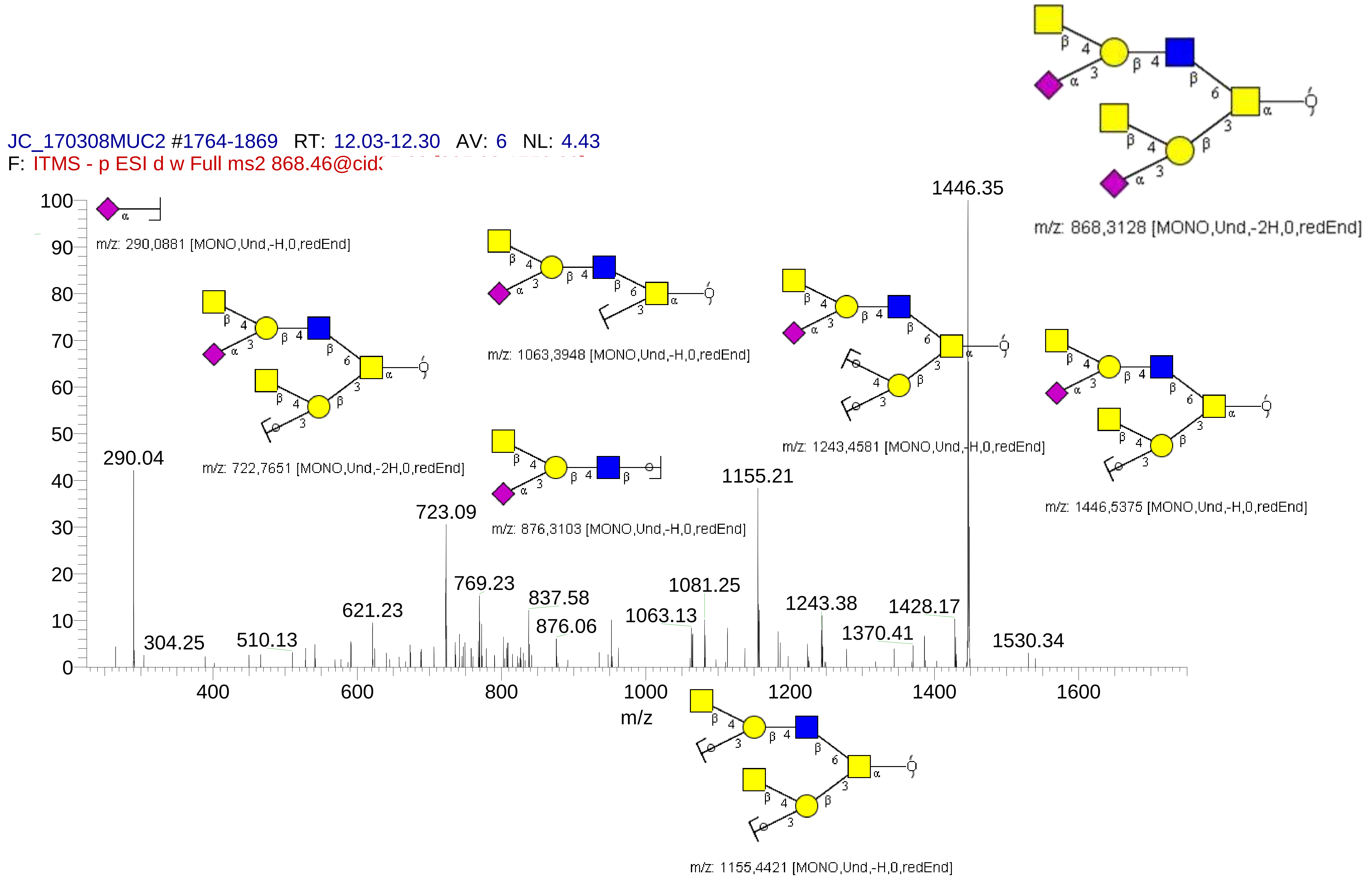

JC_170308MUC2 #1764-1869 RT: 12.03-12.30 AV: 6 NL: 4.43
F: ITMS - p ESI d w Full ms2 868.46@cid3
m/z: 868,3128 [MONO,Und,-2H,0,redEnd]
m/z: 290,0881 [MONO,Und,-H,0,redEnd]
m/z: 1063,3948 [MONO,Und,-H,0,redEnd]
m/z: 722,7651 [MONO,Und,-2H,0,redEnd]
m/z: 1243,4581 [MONO,Und,-H,0,redEnd]
m/z: 876,3103 [MONO,Und,-H,0,redEnd]
m/z: 1446,5375 [MONO,Und,-H,0,redEnd]
m/z: 1155,4421 [MONO,Und,-H,0,redEnd]
1446.35
290.04
723.09
1155.21
769.23
837.58
876.06
1081.25
1063.13
621.23
510.13
304.25
1243.38
1428.17
1370.41
1530.34
m/z

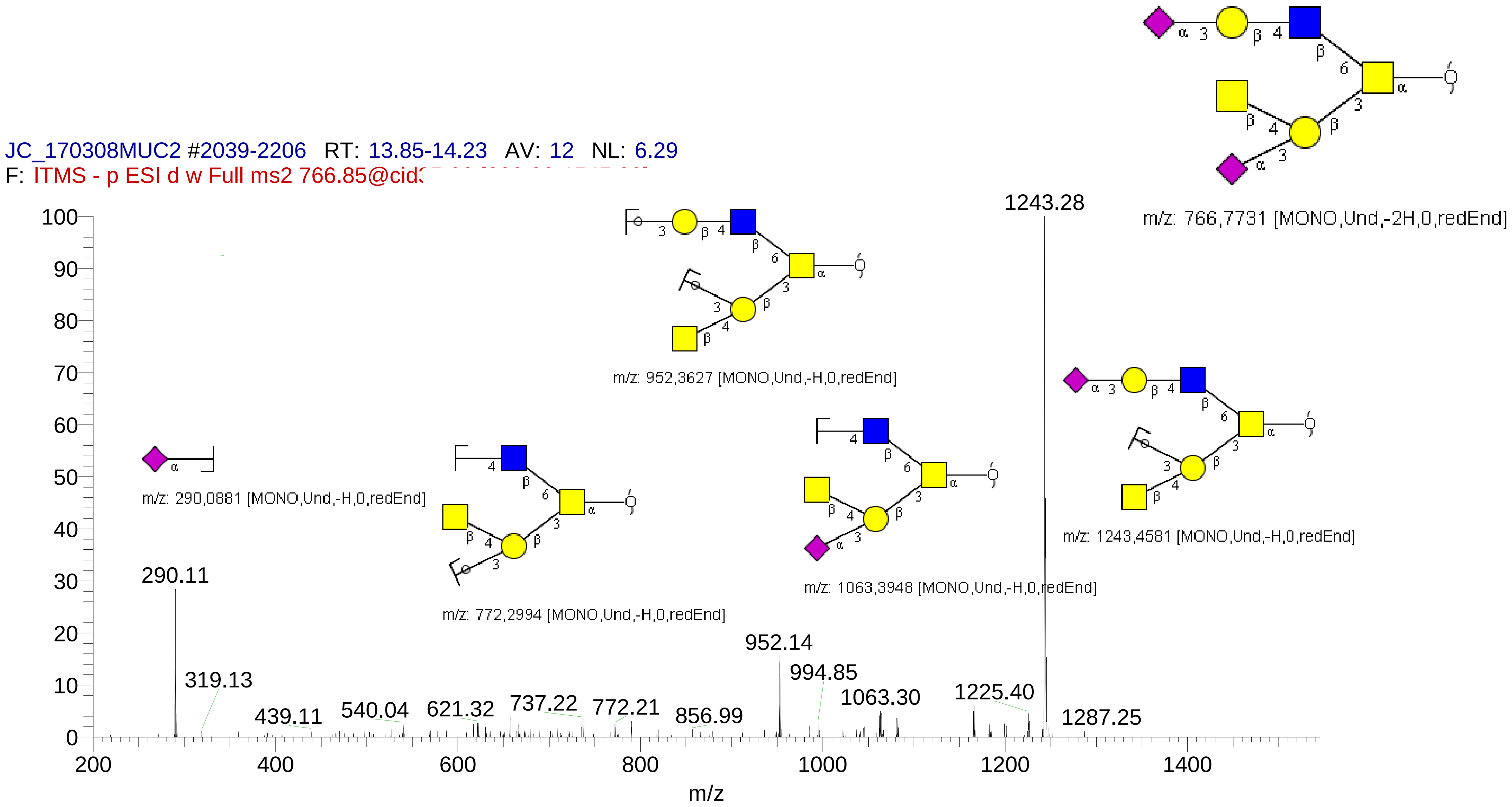

JC_170308MUC2 #2039-2206 RT: 13.85-14.23 AV: 12 NL: 6.29
F: ITMS - p ESI d w Full ms2 766.85@cid
m/z: 766,7731 [MONO,Und,-2H,0,redEnd]
m/z: 952,3627 [MONO,Und,-H,0,redEnd]
m/z: 290,0881 [MONO,Und,-H,0,redEnd]
m/z: 772,2994 [MONO,Und,-H,0,redEnd]
m/z: 1063,3948 [MONO,Und,-H,0,redEnd]
m/z: 1243,4581 [MONO,Und,-H,0,redEnd]
1243.28
290.11
319.13
439.11
540.04
621.32
737.22
772.21
856.99
952.14
994.85
1063.30
1225.40
1287.25
m/z

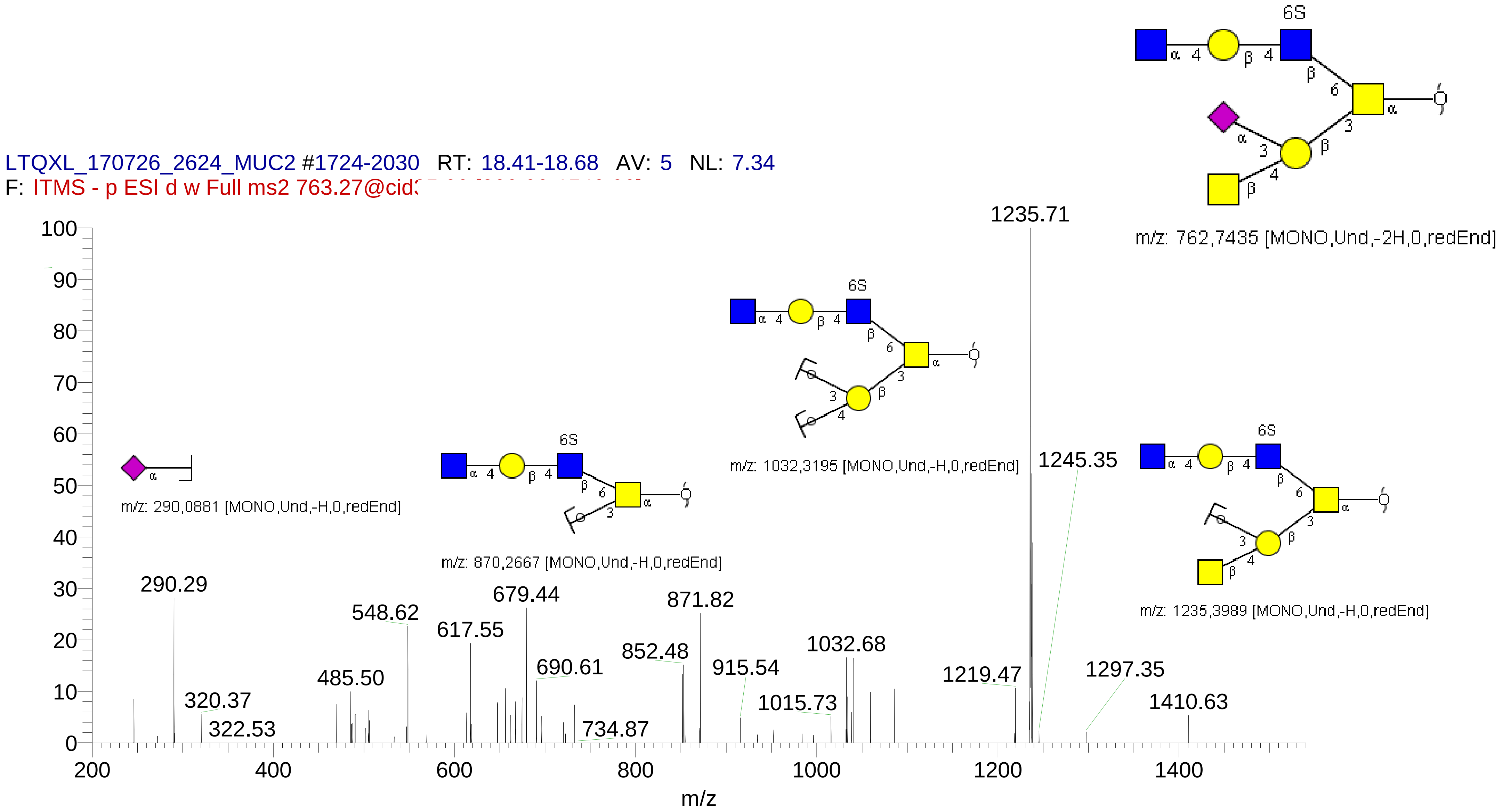
LTQXL_170726_2624_MUC2 #1724-2030 RT: 18.41-18.68 AV: 5 NL: 7.34
F: ITMS - p ESI d w Full ms2 763.27@cid
m/z: 762,7435 [MONO,Und,-2H,0,redEnd]
m/z: 290,0881 [MONO,Und,-H,0,redEnd]
m/z: 870,2667 [MONO,Und,-H,0,redEnd]
m/z: 1032,3195 [MONO,Und,-H,0,redEnd]
m/z: 1235,3989 [MONO,Und,-H,0,redEnd]
1235.71
1245.35
290.29
548.62
617.55
679.44
690.61
871.82
852.48
915.54
1032.68
1015.73
1219.47
1297.35
1410.63
485.50
320.37
322.53
734.87
m/z

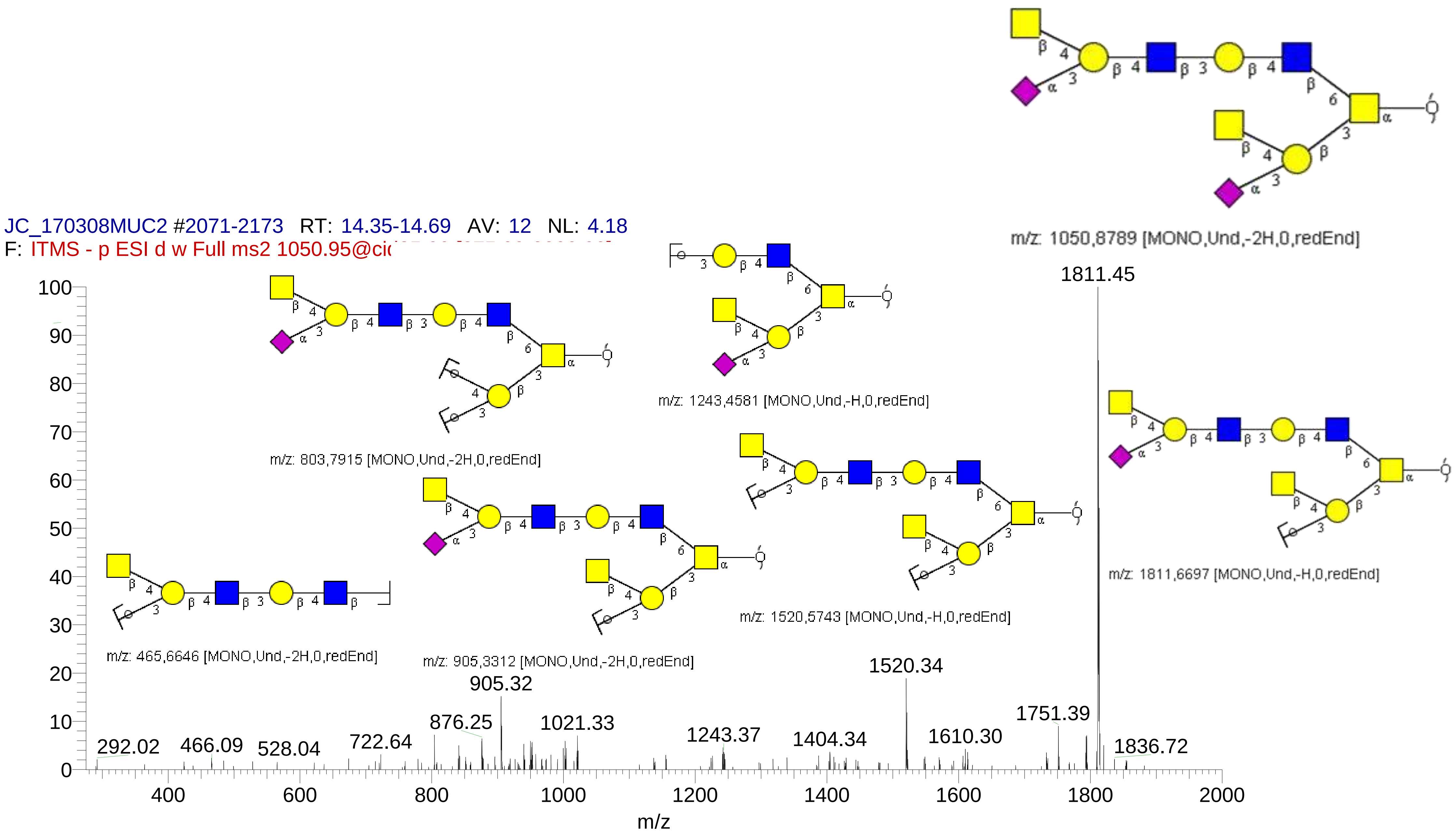

JC_170308MUC2 #2071-2173 RT: 14.35-14.69 AV: 12 NL: 4.18
F: ITMS - p ESI d w Full ms2 1050.95@cid
m/z: 1050,8789 [MONO,Und,-2H,0,redEnd]
m/z: 803,7915 [MONO,Und,-2H,0,redEnd]
m/z: 1243,4581 [MONO,Und,-H,0,redEnd]
m/z: 1811,6697 [MONO,Und,-H,0,redEnd]
m/z: 1520,5743 [MONO,Und,-H,0,redEnd]
m/z: 465,6646 [MONO,Und,-2H,0,redEnd]
m/z: 905,3312 [MONO,Und,-2H,0,redEnd]
1811.45
1520.34
905.32
876.25
1021.33
1243.37
1404.34
1610.30
1751.39
1836.72
292.02
466.09
528.04
722.64
m/z

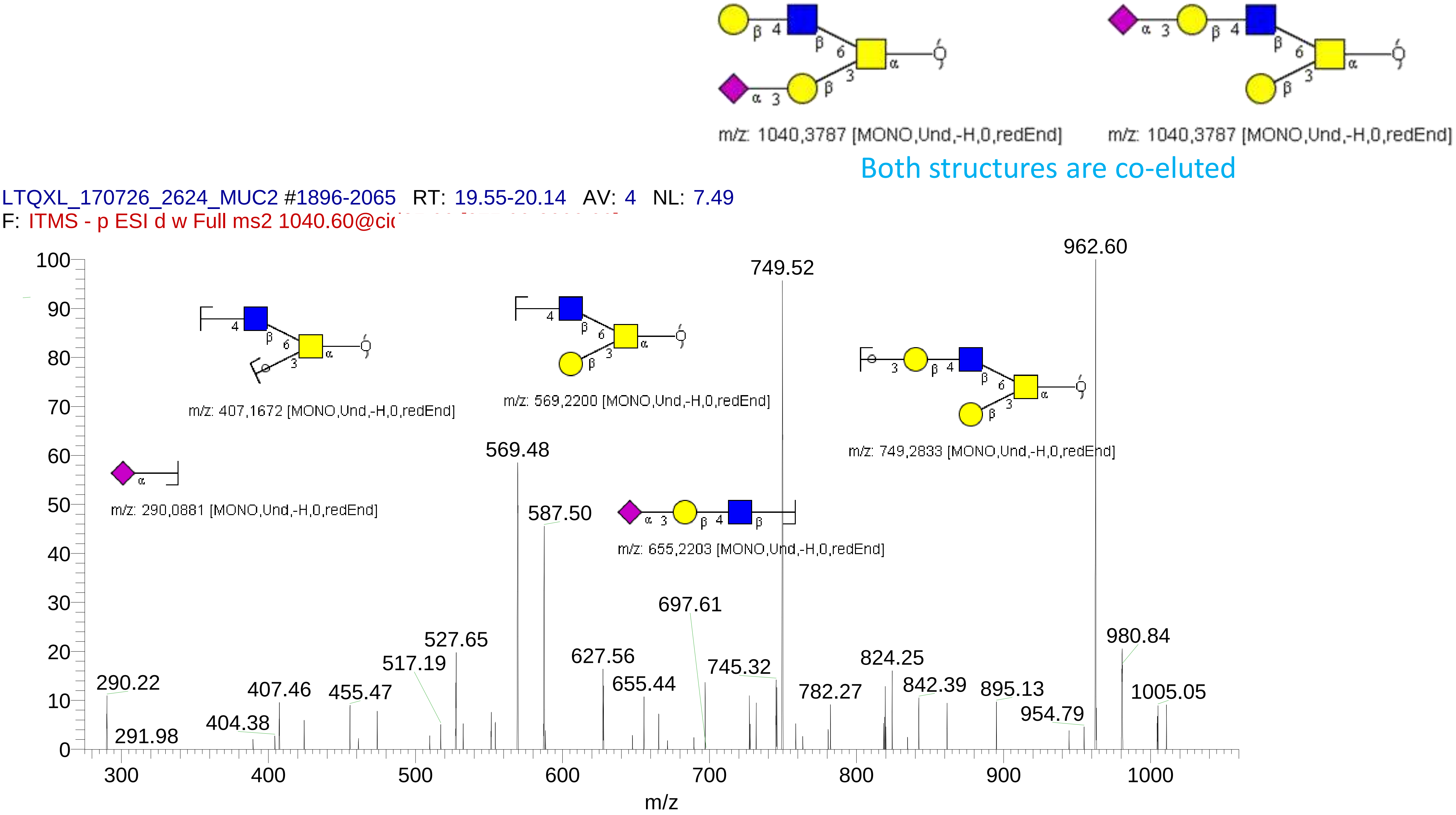
m/z: 1040,3787 [MONO,Und,-H,0,redEnd]
m/z: 1040,3787 [MONO,Und,-H,0,redEnd]
Both structures are co-eluted
LTQXL_170726_2624_MUC2 #1896-2065 RT: 19.55-20.14 AV: 4 NL: 7.49
F: ITMS - p ESI d w Full ms2 1040.60@cid
962.60
749.52
569.48
587.50
697.61
527.65
517.19
627.56
655.44
745.32
824.25
842.39
782.27
895.13
954.79
980.84
1005.05
290.22
291.98
407.46
404.38
455.47
m/z: 407,1672 [MONO,Und,-H,0,redEnd]
m/z: 569,2200 [MONO,Und,-H,0,redEnd]
m/z: 749,2833 [MONO,Und,-H,0,redEnd]
m/z: 290,0881 [MONO,Und,-H,0,redEnd]
m/z: 655,2203 [MONO,Und,-H,0,redEnd]
m/z

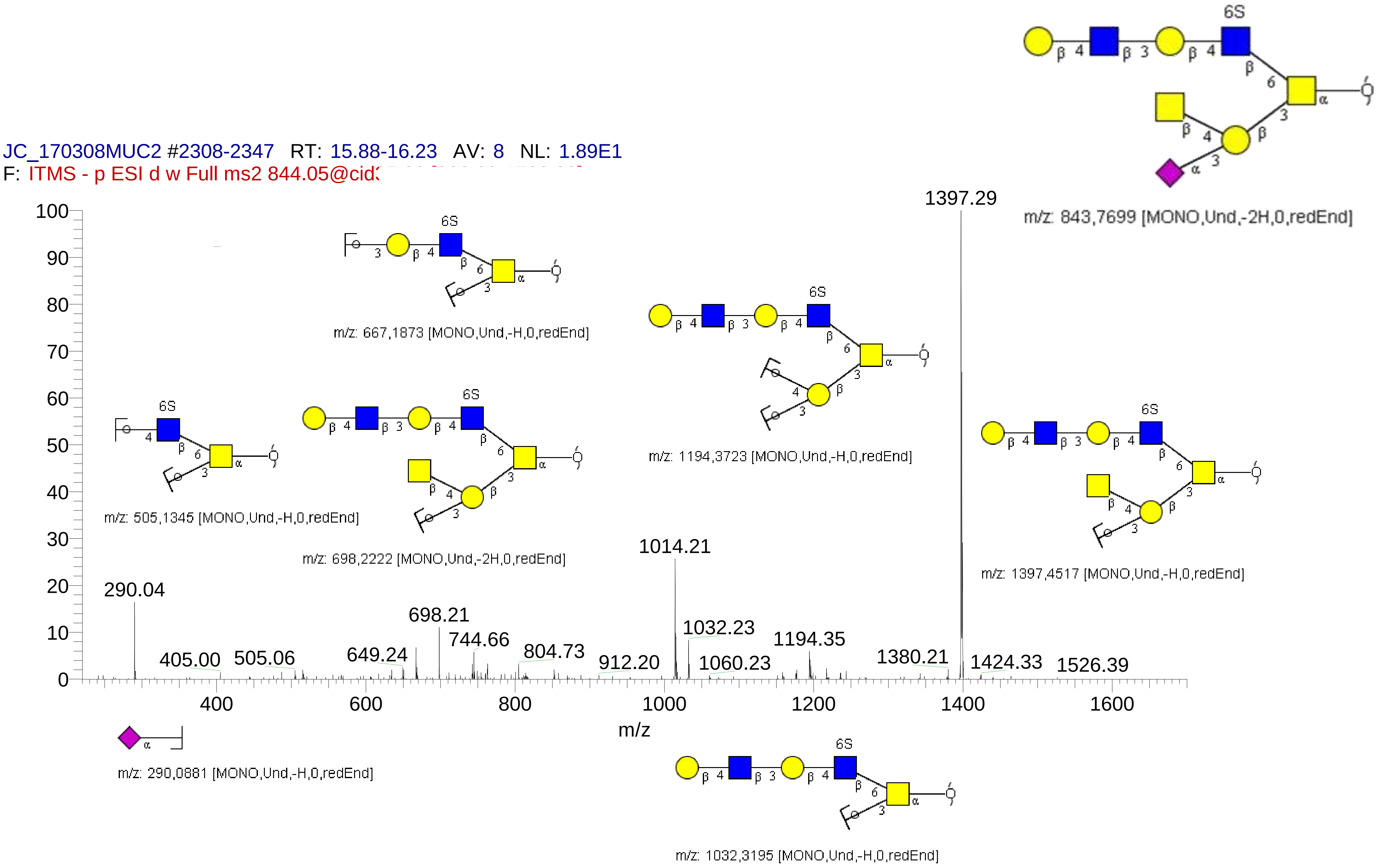

6S
m/z: 843,7699 [MONO,Und,-2H,0,redEnd]
JC_170308MUC2 #2308-2347 RT: 15.88-16.23 AV: 8 NL: 1.89E1
F: ITMS - p ESI d w Full ms2 844.05@cid3
1397.29
m/z: 667,1873 [MONO,Und,-H,0,redEnd]
m/z: 1194,3723 [MONO,Und,-H,0,redEnd]
m/z: 505,1345 [MONO,Und,-H,0,redEnd]
m/z: 698,2222 [MONO,Und,-2H,0,redEnd]
m/z: 1397,4517 [MONO,Und,-H,0,redEnd]
1014.21
290.04
698.21
744.66
804.73
649.24
405.00
505.06
912.20
1032.23
1060.23
1194.35
1380.21
1424.33
1526.39
m/z
m/z: 290,0881 [MONO,Und,-H,0,redEnd]
m/z: 1032,3195 [MONO,Und,-H,0,redEnd]

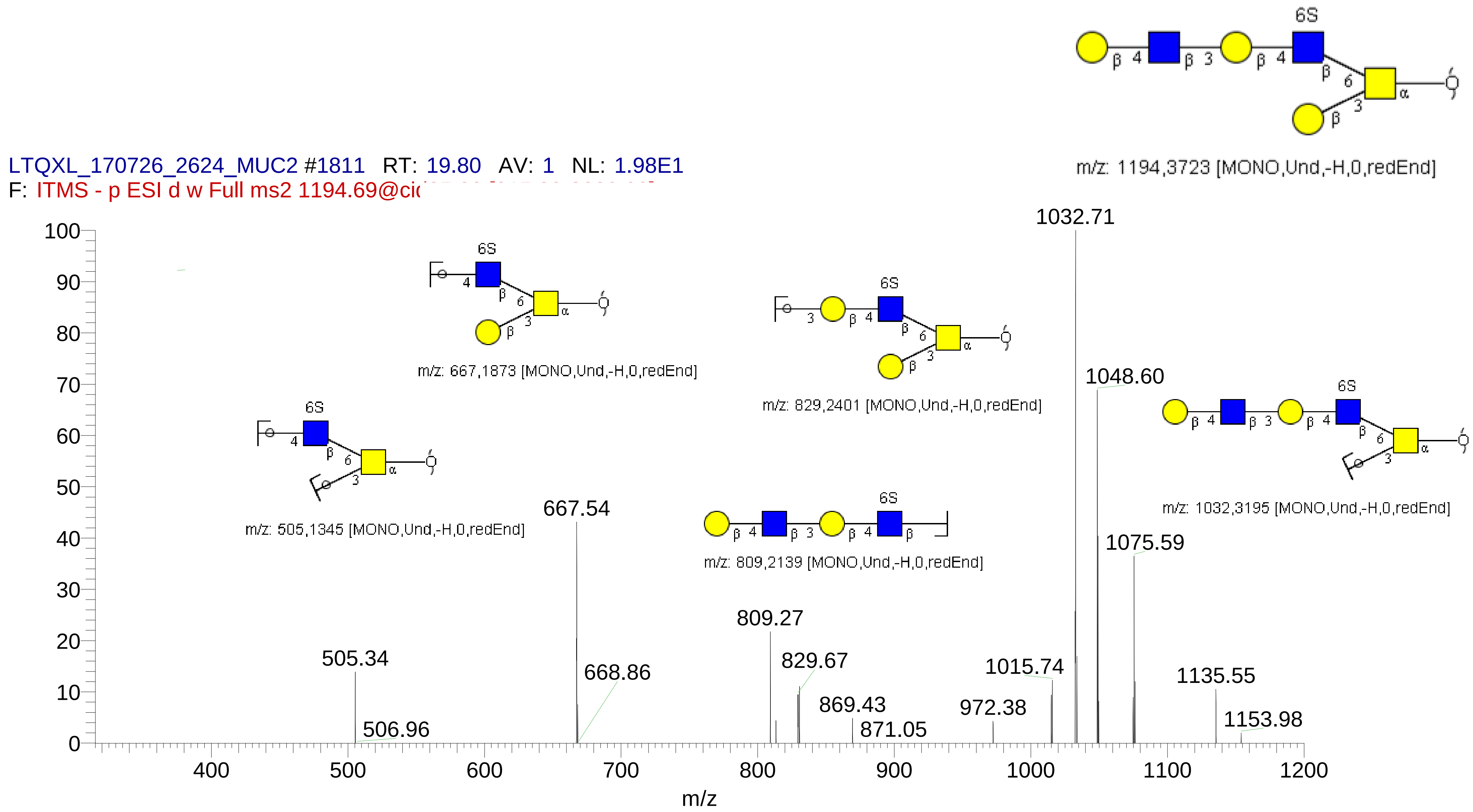

LTQXL_170726_2624_MUC2 #1811 RT: 19.80 AV: 1 NL: 1.98E1
F: ITMS - p ESI d w Full ms2 1194.69@ci
m/z: 1194,3723 [MONO,Und,-H,0,redEnd]
m/z: 667,1873 [MONO,Und,-H,0,redEnd]
m/z: 829,2401 [MONO,Und,-H,0,redEnd]
m/z: 505,1345 [MONO,Und,-H,0,redEnd]
m/z: 809,2139 [MONO,Und,-H,0,redEnd]
m/z: 1032,3195 [MONO,Und,-H,0,redEnd]
6S
1032.71
1048.60
1075.59
667.54
809.27
505.34
668.86
829.67
1015.74
1135.55
869.43
972.38
1153.98
506.96
871.05
m/z

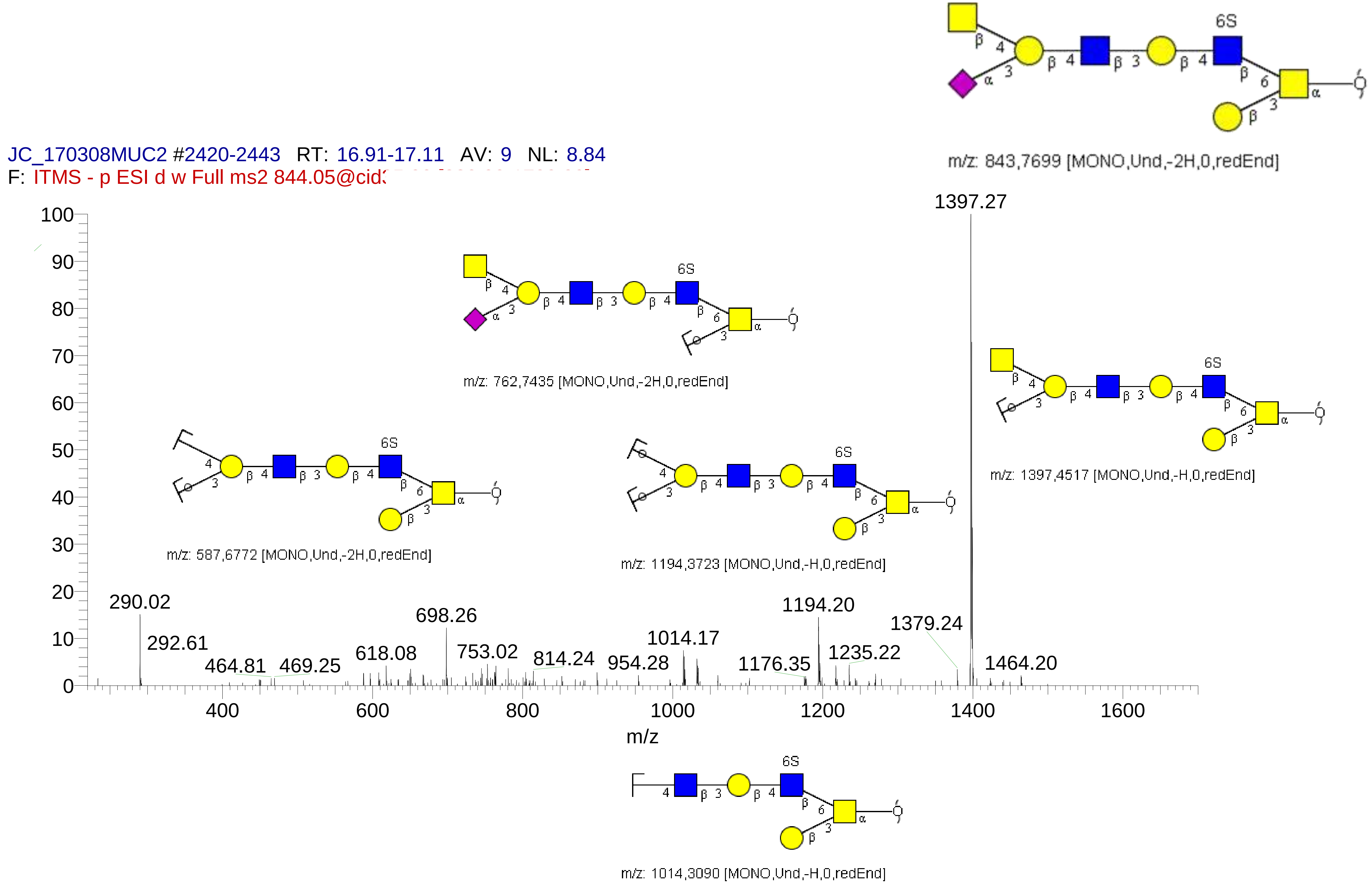

JC_170308MUC2 #2420-2443 RT: 16.91-17.11 AV: 9 NL: 8.84
F: ITMS - p ESI d w Full ms2 844.05@cid
m/z: 843,7699 [MONO,Und,-2H,0,redEnd]
m/z: 762,7435 [MONO,Und,-2H,0,redEnd]
m/z: 1397,4517 [MONO,Und,-H,0,redEnd]
m/z: 587,6772 [MONO,Und,-2H,0,redEnd]
m/z: 1194,3723 [MONO,Und,-H,0,redEnd]
m/z: 1014,3090 [MONO,Und,-H,0,redEnd]
1397.27
290.02
292.61
464.81
469.25
618.08
698.26
753.02
814.24
954.28
1014.17
1176.35
1194.20
1235.22
1379.24
1464.20
m/z

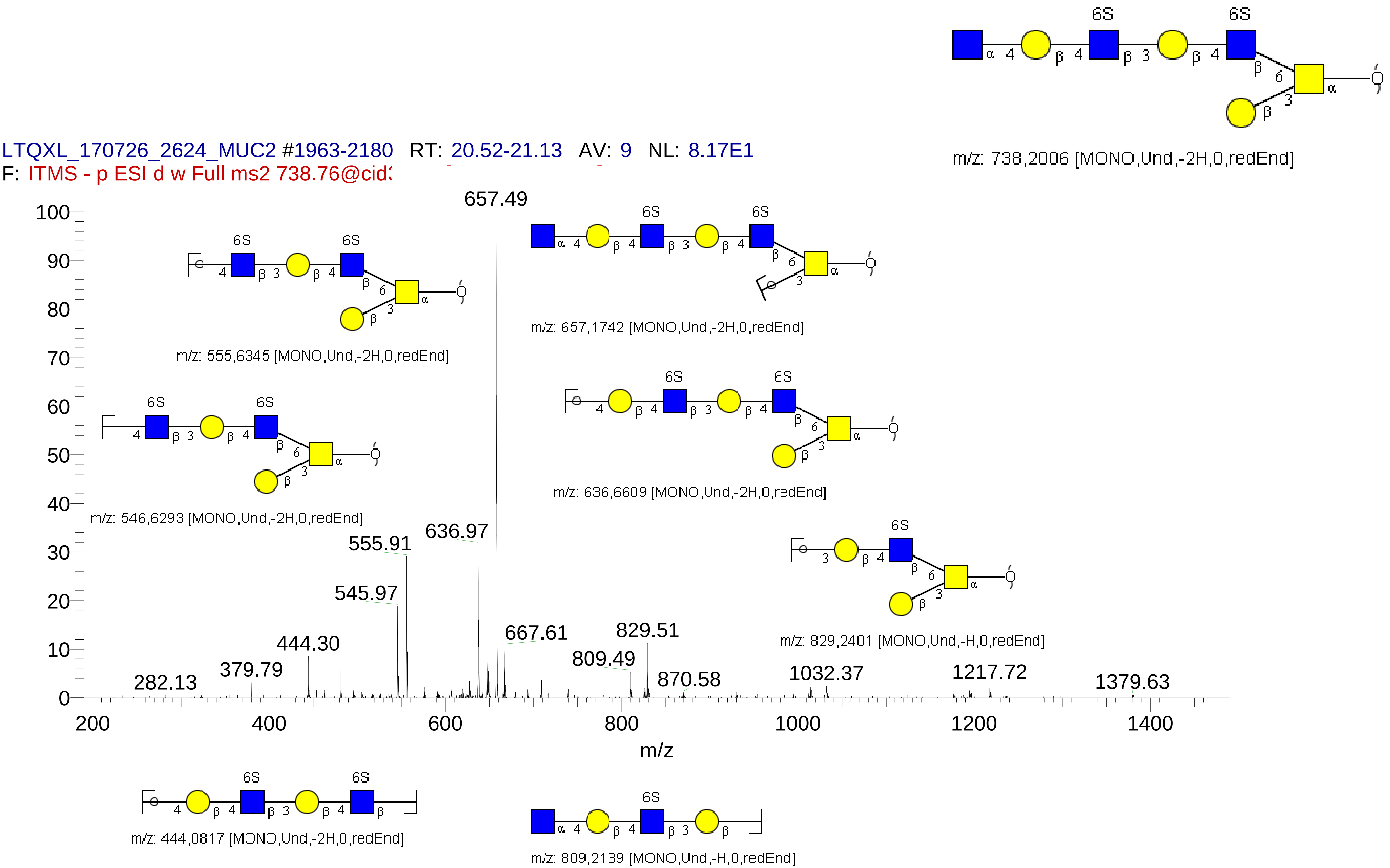
LTQXL_170726_2624_MUC2 #1963-2180 RT: 20.52-21.13 AV: 9 NL: 8.17E1
F: ITMS - p ESI d w Full ms2 738.76@cid3
m/z: 738,2006 [MONO,Und,-2H,0,redEnd]
657.49
m/z: 555,6345 [MONO,Und,-2H,0,redEnd]
m/z: 657,1742 [MONO,Und,-2H,0,redEnd]
m/z: 546,6293 [MONO,Und,-2H,0,redEnd]
m/z: 636,6609 [MONO,Und,-2H,0,redEnd]
m/z: 829,2401 [MONO,Und,-H,0,redEnd]
555.91
636.97
545.97
444.30
379.79
282.13
667.61
829.51
809.49
870.58
1032.37
1217.72
1379.63
m/z
m/z: 444,0817 [MONO,Und,-2H,0,redEnd]
m/z: 809,2139 [MONO,Und,-H,0,redEnd]

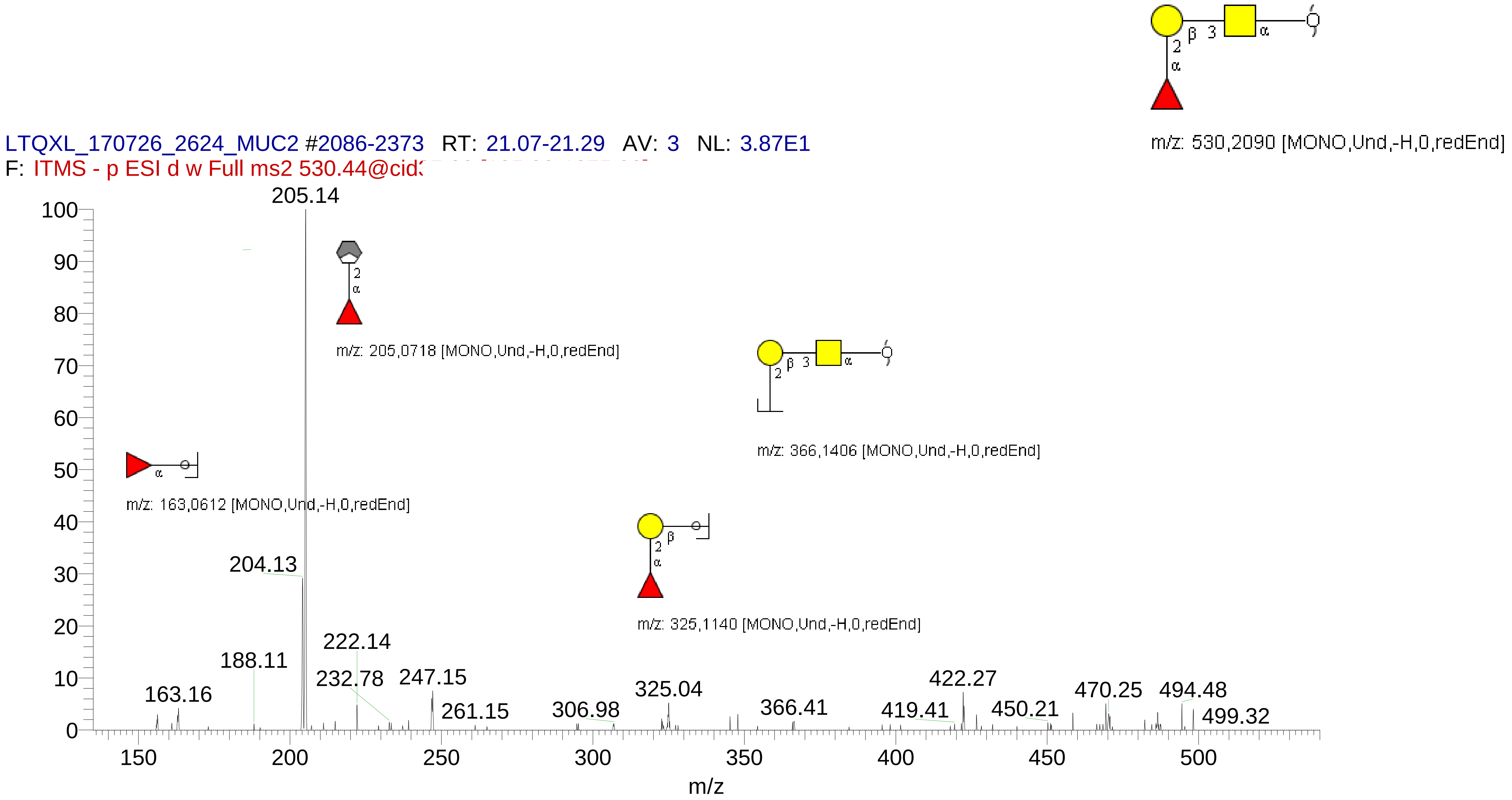

m/z: 530,2090 [MONO,Und,-H,0,redEnd]
LTQXL_170726_2624_MUC2 #2086-2373 RT: 21.07-21.29 AV: 3 NL: 3.87E1
F: ITMS - p ESI d w Full ms2 530.44@cid
205.14
m/z: 205,0718 [MONO,Und,-H,0,redEnd]
m/z: 366,1406 [MONO,Und,-H,0,redEnd]
m/z: 163,0612 [MONO,Und,-H,0,redEnd]
m/z: 325,1140 [MONO,Und,-H,0,redEnd]
204.13
222.14
188.11
232.78
247.15
163.16
261.15
306.98
325.04
366.41
419.41
422.27
450.21
470.25
494.48
499.32
100
90
80
70
60
50
40
30
20
10
0
150
200
250
300
350
400
450
500
m/z

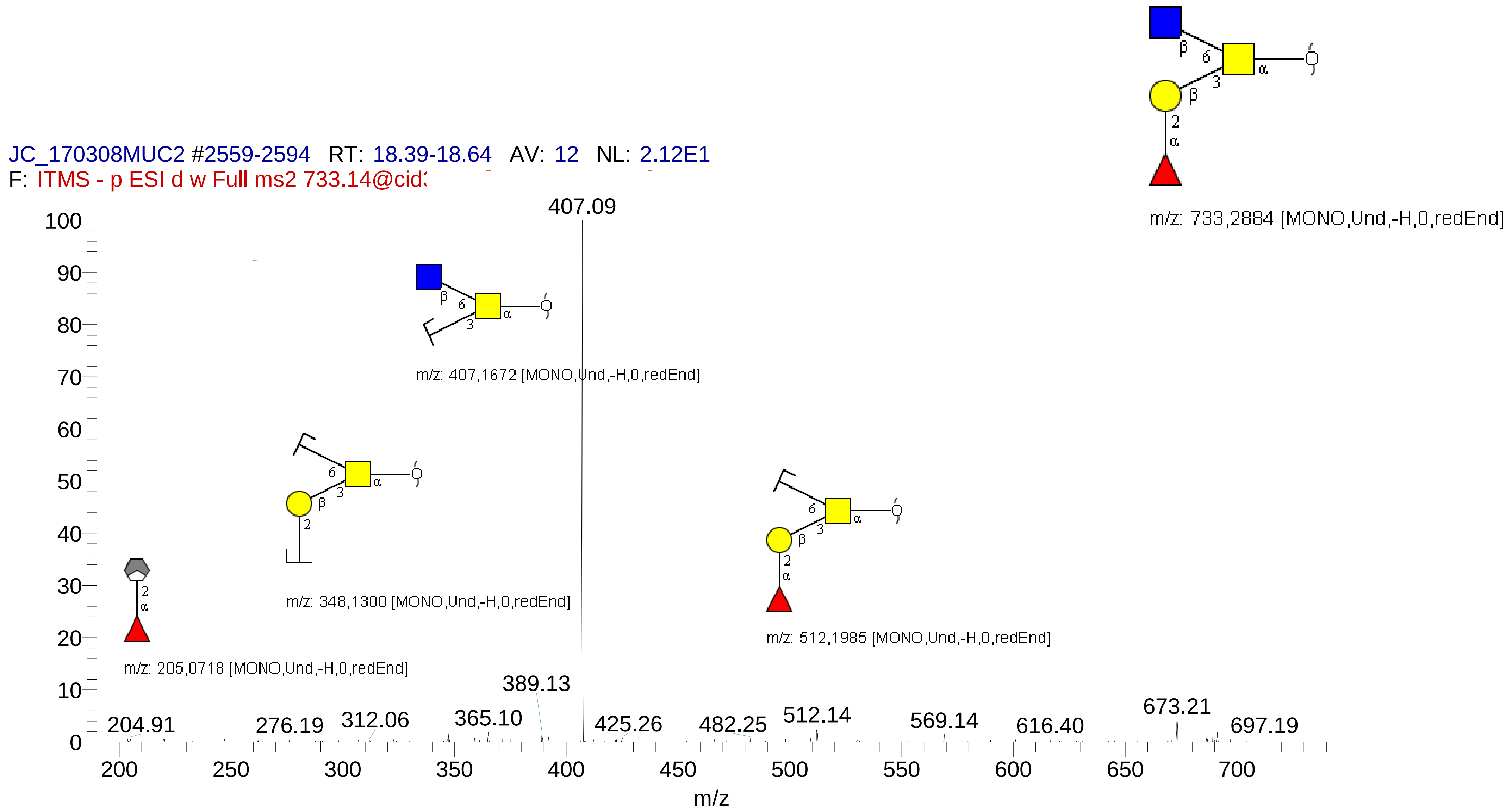
JC_170308MUC2 #2559-2594 RT: 18.39-18.64 AV: 12 NL: 2.12E1
F: ITMS - p ESI d w Full ms2 733.14@cid
m/z: 733,2884 [MONO,Und,-H,0,redEnd]
m/z: 407,1672 [MONO,Und,-H,0,redEnd]
m/z: 348,1300 [MONO,Und,-H,0,redEnd]
m/z: 512,1985 [MONO,Und,-H,0,redEnd]
m/z: 205,0718 [MONO,Und,-H,0,redEnd]
407.09
389.13
204.91
276.19
312.06
365.10
425.26
482.25
512.14
569.14
616.40
673.21
697.19
m/z

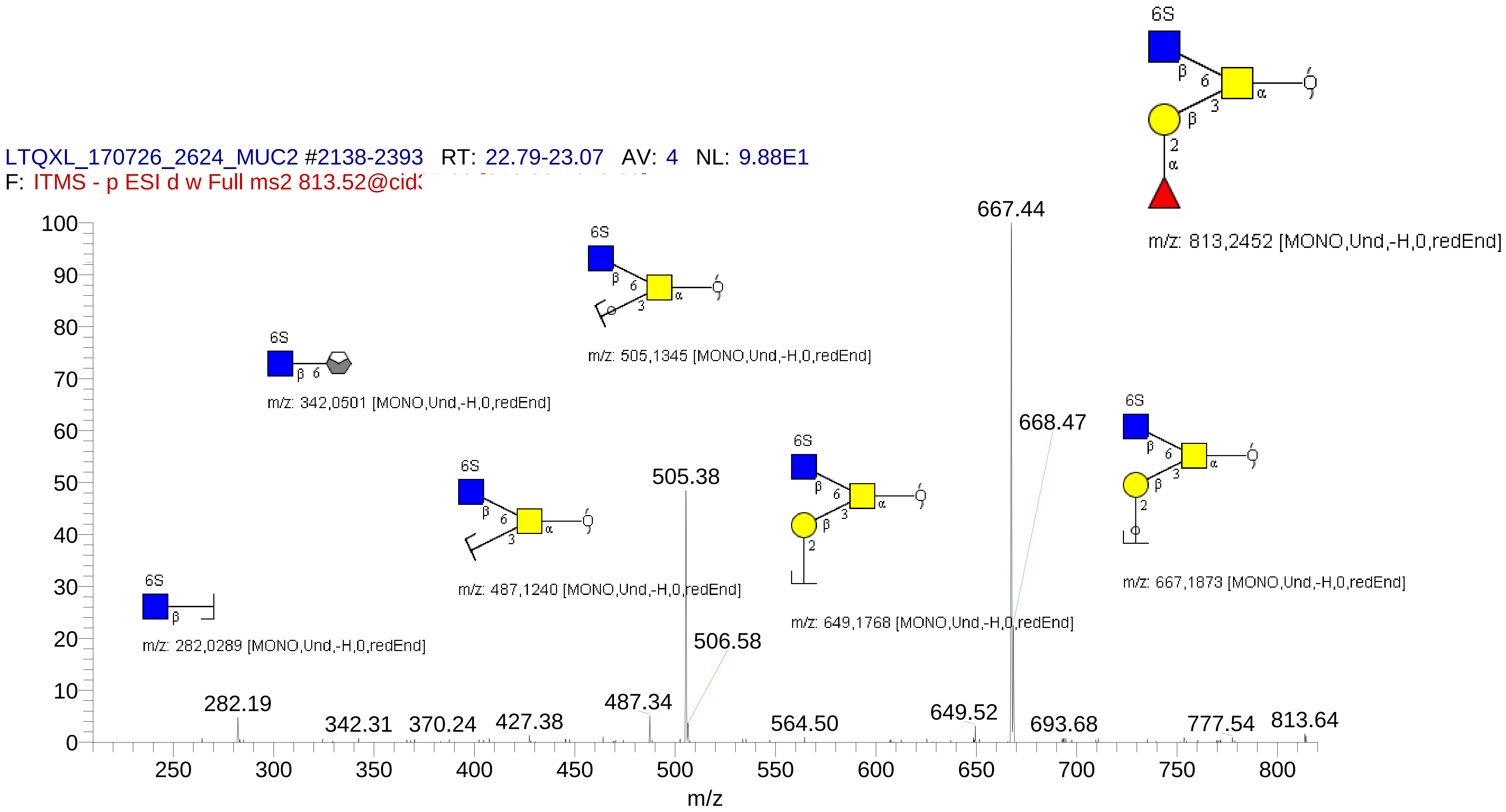
LTQXL_170726_2624_MUC2 #2138-2393 RT: 22.79-23.07 AV: 4 NL: 9.88E1
F: ITMS - p ESI d w Full ms2 813.52@cid3
6S
m/z: 813,2452 [MONO,Und,-H,0,redEnd]
667.44
m/z: 505,1345 [MONO,Und,-H,0,redEnd]
m/z: 342,0501 [MONO,Und,-H,0,redEnd]
668.47
505.38
m/z: 487,1240 [MONO,Und,-H,0,redEnd]
m/z: 667,1873 [MONO,Und,-H,0,redEnd]
m/z: 649,1768 [MONO,Und,-H,0,redEnd]
m/z: 282,0289 [MONO,Und,-H,0,redEnd]
506.58
282.19
342.31
370.24
427.38
487.34
564.50
649.52
693.68
777.54
813.64
m/z

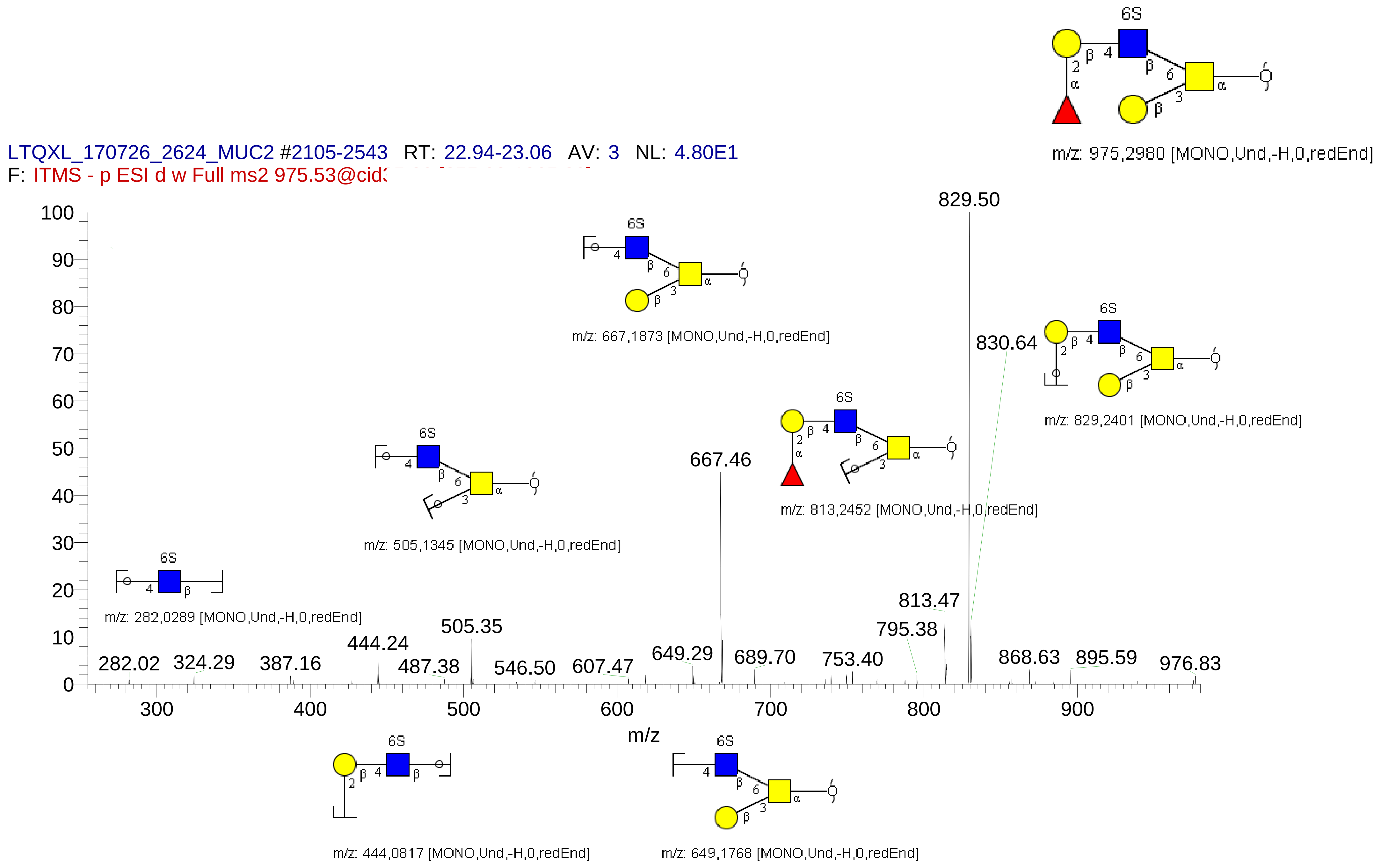

LTQXL_170726_2624_MUC2 #2105-2543 RT: 22.94-23.06 AV: 3 NL: 4.80E1
F: ITMS - p ESI d w Full ms2 975.53@cid
m/z: 975,2980 [MONO,Und,-H,0,redEnd]
m/z: 667,1873 [MONO,Und,-H,0,redEnd]
m/z: 829,2401 [MONO,Und,-H,0,redEnd]
m/z: 813,2452 [MONO,Und,-H,0,redEnd]
m/z: 505,1345 [MONO,Und,-H,0,redEnd]
m/z: 282,0289 [MONO,Und,-H,0,redEnd]
m/z: 444,0817 [MONO,Und,-H,0,redEnd]
m/z: 649,1768 [MONO,Und,-H,0,redEnd]
6S
829.50
830.64
667.46
813.47
795.38
505.35
444.24
487.38
546.50
607.47
649.29
689.70
753.40
868.63
895.59
976.83
282.02
324.29
387.16
m/z

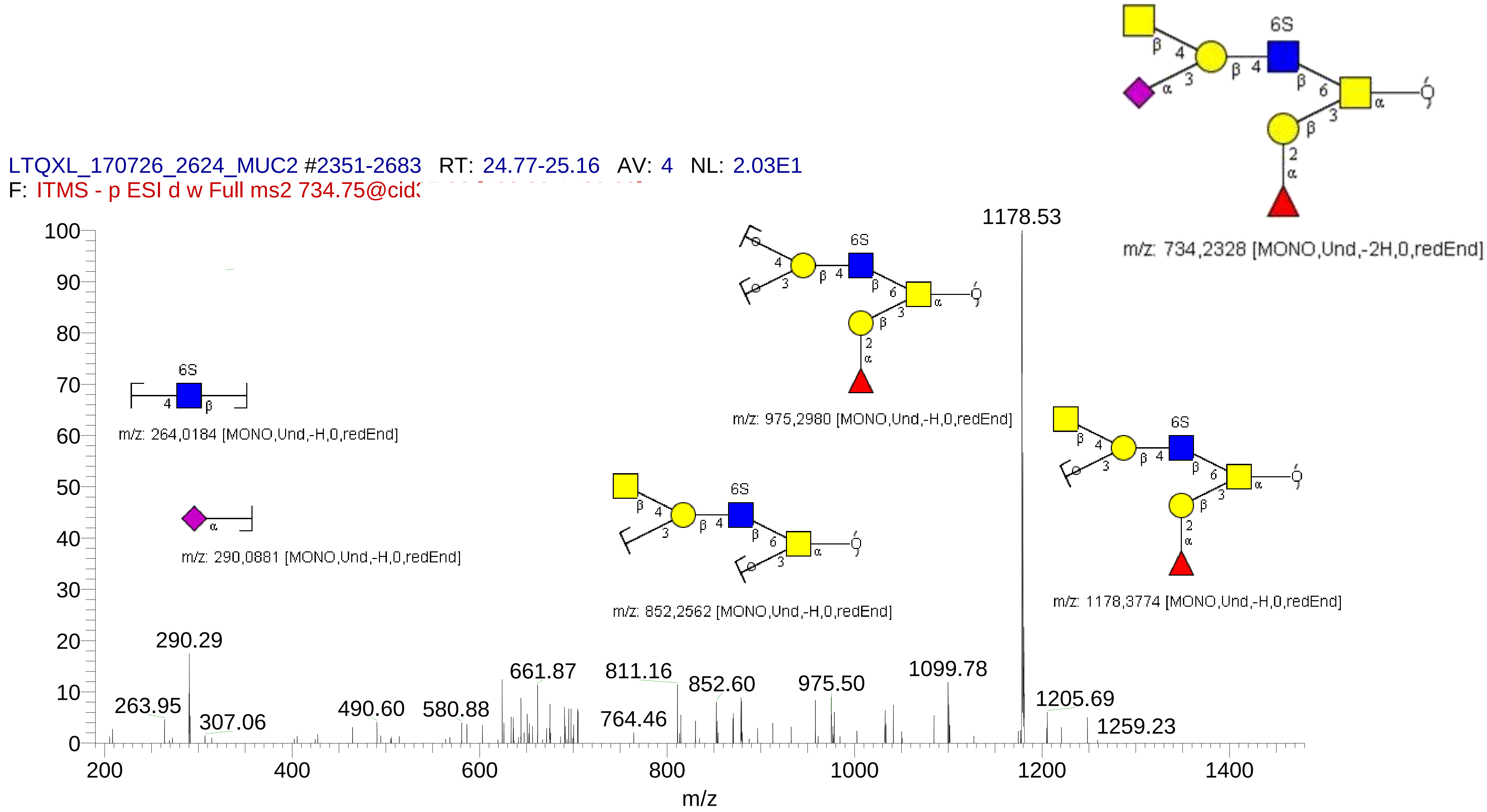

LTQXL_170726_2624_MUC2 #2351-2683 RT: 24.77-25.16 AV: 4 NL: 2.03E1
F: ITMS - p ESI d w Full ms2 734.75@cid
m/z: 734,2328 [MONO,Und,-2H,0,redEnd]
m/z: 264,0184 [MONO,Und,-H,0,redEnd]
m/z: 290,0881 [MONO,Und,-H,0,redEnd]
m/z: 975,2980 [MONO,Und,-H,0,redEnd]
m/z: 852,2562 [MONO,Und,-H,0,redEnd]
m/z: 1178,3774 [MONO,Und,-H,0,redEnd]
1178.53
290.29
263.95
307.06
490.60
580.88
661.87
811.16
764.46
852.60
975.50
1099.78
1205.69
1259.23
m/z
